# Antioxidant Interventions on Muscle Function and Mass in Aging-Related Muscle Decline: A Systematic Review of Preclinical and Clinical Evidence

**DOI:** 10.3390/antiox15091167

**Published:** 2026-09-15

**Authors:** Xin Li, Ke Zhou, Ronald Man Yeung Wong, Wing Hoi Cheung, Can Cui

**Affiliations:** 1 Musculoskeletal Research Laboratory, Department of Orthopaedics and Traumatology, The Chinese University of Hong Kong, Hong Kong SAR, China; 1155249699@link.cuhk.edu.hk (X.L.); 1155252750@link.cuhk.edu.hk (K.Z.); ronald.wong@cuhk.edu.hk (R.M.Y.W.); 2 Li Ka Shing Institute of Health Sciences, The Chinese University of Hong Kong, Hong Kong SAR, China

**Keywords:** antioxidants, sarcopenia, aging-related muscle decline, oxidative stress, mitochondrial dysfunction, aging, systematic review

## Abstract

Oxidative stress contributes to aging-related muscle decline, but antioxidant interventions may affect functional and structural outcomes differently. This systematic review synthesized clinical, animal, and cellular evidence across clinically defined sarcopenia and broader models of aging-related muscle decline. PubMed, Embase, Web of Science, and Cochrane Central Register of Controlled Trials (CENTRAL) were searched through 11 July 2026. Eligible studies tested antioxidant interventions in older adults or aging-related preclinical models and were appraised using the Cochrane Risk of Bias 2 (RoB 2) tool, a modified Systematic Review Centre for Laboratory animal Experimentation (SYRCLE) risk-of-bias tool, and a modified in vitro checklist. Forty-four publications were included: eight clinical trials, 26 animal datasets, and 18 cellular datasets, with eight publications contributing both animal and cellular evidence. Comparative clinical benefits were reported primarily for strength or power (7/8 studies), with less support for physical performance (2/6 studies assessing this domain) or body composition-related muscle quantity (2/8 studies). Selected preclinical studies likewise showed functional gains without parallel structural improvement, although effects varied by model and intervention. Preclinical findings implicated antioxidant defense, mitochondrial maintenance, inflammation, and proteostasis, but most pathway evidence was associative and direct experimental validation was limited. Clinical evidence was further constrained by small samples, short interventions, combined nutritional or exercise co-interventions, and heterogeneous outcomes. Current evidence does not support a consistent increase in muscle quantity with antioxidant interventions. The observed pattern appears context dependent and requires confirmation in adequately powered, phenotype-stratified trials assessing muscle strength, physical performance, muscle quantity, and mechanistic endpoints.

## 1. Introduction

Sarcopenia is an age-related skeletal muscle disorder defined by the concurrent presence of low muscle mass and low muscle strength, while physical performance represents an important related outcome rather than a required diagnostic component under the Asian Working Group for Sarcopenia (AWGS) 2025 consensus [1]. Its global prevalence is approximately 10% [2], although estimates vary by population and diagnostic criteria. The AWGS provides regionally relevant assessment guidance [1]. Sarcopenia is associated with functional decline, systemic metabolic disturbances, and an increased risk of adverse health outcomes, including disability and mortality [2,3,4].

The pathogenesis of sarcopenia involves a multifaceted interplay of neuromuscular dysfunction, protein metabolic imbalance, and chronic inflammation [5]. A key contributor to this process is mitochondrial dysfunction, which has been implicated in skeletal muscle aging and sarcopenia [6]. Because skeletal muscle relies heavily on oxidative metabolism, excessive reactive oxygen species (ROS) production and impaired redox homeostasis may contribute to age-related muscle dysfunction [7]. In aging skeletal muscle, impaired mitochondrial quality control, including defective mitophagy, contributes to the persistence of dysfunctional mitochondria and disrupted muscle homeostasis [8]. Cellular senescence is also increasingly recognized as an important contributor to skeletal muscle aging and sarcopenia, where it may impair regenerative signaling, tissue homeostasis, and inflammatory regulation [9]. Importantly, experimental evidence indicates that aging and oxidative stress can impair excitation-contraction coupling (ECC) through alterations in membrane excitability and sarcoplasmic-reticulum calcium handling [10]. More broadly, the relative contribution of neuromuscular denervation, mitochondrial dysfunction, chronic inflammaging, nutritional deficiency, and anabolic resistance varies among individuals, indicating that sarcopenia comprises clinically and biologically heterogeneous phenotypes rather than a single uniform disorder [5].

Current management relies primarily on exercise, with nutritional interventions used as important adjuncts [1,11]. Antioxidant strategies have also attracted interest as potential adjuncts in aging-related muscle decline, with observational, clinical, and preclinical studies suggesting the possible benefits of selected vitamins and polyphenols [12,13,14,15]. Because exercise-induced ROS also act as physiological signals that contribute to mitochondrial and other training adaptations, the effects of antioxidant interventions are likely to depend on dose, timing, baseline antioxidant capacity, and exercise context rather than on indiscriminate ROS suppression [16]. Clinical findings nevertheless remain inconsistent, and their relationship to model-specific preclinical mechanisms is unclear.

Previous narrative reviews have examined flavonoids [17], microalgae-derived molecules [18], green tea polyphenols [19], and curcumin [20]. A recent meta-analysis evaluated antioxidant interventions with or without exercise in clinical populations [21], but did not integrate clinical outcomes with model-specific preclinical mechanisms. Because muscle strength and physical performance represent related but distinct functional domains, we synthesized them separately and examined whether changes in either domain occurred without parallel changes in body composition-related muscle quantity. This relationship was treated as an exploratory pattern rather than a predefined effect of antioxidant intervention. Accordingly, this review integrated clinical, animal, and cellular evidence across clinically defined sarcopenia and broader aging-related muscle decline, while distinguishing preclinical model contexts and the level of mechanistic validation.

## 2. Materials and Methods

### 2.1. Search Strategy

PubMed, Embase, Web of Science, and the Cochrane Central Register of Controlled Trials (CENTRAL) were searched on 11 July 2026. The strategy combined Medical Subject Headings with free-text terms for sarcopenia, muscle wasting, muscle loss, antioxidants, vitamins C and E, polyphenols, and flavonoids. Boolean operators were used to combine concepts and maximize retrieval sensitivity (Supplementary Table S1). No initial date restriction was applied. Reference lists of included studies and related reviews were also searched manually. Reporting followed the Preferred Reporting Items for Systematic Reviews and Meta-Analyses guidelines (PRISMA) [22]. Backward citation searching identified no additional eligible reports. This review was not prospectively registered in International Prospective Register of Systematic Reviews (PROSPERO) or another public repository.

### 2.2. Eligibility Criteria

Clinical studies were eligible if they enrolled adults with study-defined sarcopenia or a clearly defined form of aging-related muscle decline, including frailty, low muscle strength, low muscle mass, or mobility limitation. Because diagnostic definitions and age thresholds varied across the clinical literature, eligibility was based on the presence of an aging-related muscle phenotype rather than a uniform chronological-age cutoff. For interpretation of the clinical evidence, studies using explicit sarcopenia-related diagnostic or muscle-mass criteria were distinguished from those enrolling older adults with broader age-associated muscle impairment.

Animal studies were eligible if they investigated skeletal muscle outcomes in models relevant to aging-related muscle decline. These models included natural aging models, accelerated-aging or deficiency models, chemically induced aging-like models, and experimentally induced muscle-atrophy or disuse models. Chemically induced aging-like models included D-galactose exposure, whereas experimentally induced atrophy or disuse models included dexamethasone administration and hindlimb unloading. These induced models were included to examine specific oxidative, catabolic, or disuse-related mechanisms but were not considered biologically equivalent to spontaneous age-related sarcopenia.

Cellular studies included skeletal muscle cells exposed to senescence-inducing, oxidative-stress, mitochondrial-dysfunction, or atrophy-inducing conditions. Cellular models were used primarily to examine mechanistic pathways relevant to aging-related muscle decline rather than as direct models of clinically defined sarcopenia. Non-muscle cell models were eligible only when reported within an otherwise eligible study containing a skeletal muscle model and were treated as ancillary evidence rather than independent cellular datasets.

Eligible outcomes included structural outcomes, muscle function, cellular outcomes, redox markers, mitochondrial outcomes, and relevant mechanistic pathways. In this review, structural outcomes were interpreted according to their original measurement methods. Human studies reporting lean mass, appendicular skeletal muscle mass (ASM), or skeletal muscle index (SMI) were considered measures of body composition-related muscle quantity, whereas animal studies reporting muscle weight or myofiber cross-sectional area (CSA) were interpreted as tissue-level structural outcomes. Functional outcomes were interpreted as comprising two related but distinct domains: muscle strength, reflecting force-generating capacity, and physical performance, reflecting mobility-related functional capacity. Reviews, editorials, study protocols without outcome data, conference abstracts without sufficient data, and studies lacking an eligible model, intervention, or outcome were excluded. Reports that could not be retrieved were recorded separately and were not included in full-text eligibility assessment. Studies published in English with full text available were included to ensure consistent assessment of study methodology and outcome reporting. However, this criterion may introduce language-related selection bias, which is acknowledged as a limitation of the review. Accordingly, the study used clinical studies to identify human outcome signals, naturally aged animal models to assess physiological plausibility, induced animal models to examine mechanism-specific proof of concept, and cellular models primarily for pathway interrogation.

### 2.3. Study Selection and Data Extraction

Two reviewers independently selected studies and extracted data. EndNote 2025 was used to remove duplicates, followed by title and abstract screening and full-text eligibility assessment. Disagreements were resolved through discussion or consultation with a third senior reviewer. A standardized form captured the author, year, design, model, intervention type, dose, duration, and principal qualitative findings. A publication was defined as a distinct report, whereas an animal or cellular dataset represented a distinct in vivo or in vitro experimental component. Publications containing both animal and cellular experiments contributed to both dataset counts but were counted once in the publication total. Non-muscle cell experiments were treated as ancillary evidence and were not counted as independent cellular datasets.

### 2.4. Quality Assessment

Two reviewers independently assessed clinical trials with the Cochrane Risk of Bias 2 tool (RoB 2) [23]. Domains covered randomization, intervention deviations, missing outcomes, outcome measurement, and selective reporting. Each domain was rated as low risk, some concerns, or high risk. Animal studies were evaluated with the modified Systematic Review Centre for Laboratory animal Experimentation (SYRCLE) risk-of-bias tool [24], which adapts the Cochrane framework for laboratory experiments. Eight domains addressed sequence generation, baseline characteristics, housing, blinding, outcome assessment, missing data, selective reporting, and other biases. Cellular studies were appraised with a modified Toxicological data Reliability Assessment Tool (ToxRTool) checklist [25] covering model characterization, intervention specification, outcome measurement, and reporting completeness. Cellular evidence was used primarily to examine mechanisms rather than support clinical conclusions.

### 2.5. Data Synthesis

Meta-analysis was considered after studies were grouped by intervention category, outcome domain, and evidence level. Quantitative pooling was not performed because even the most comparable clinical subsets remained small and differed in intervention composition, co-interventions, participant characteristics, diagnostic criteria, outcome definitions, and measurement scales. For example, strength and physical-performance outcomes were assessed using non-interchangeable measures, while interventions within the same antioxidant category differed in compound, dose, duration, and co-intervention. We therefore used a qualitative synthesis to compare the direction and consistency of effects and mechanistic convergence across evidence levels.

For study-level counting of clinical outcomes, a study was counted as showing benefit in an outcome domain if it reported a statistically significant between-group difference in change, a group-by-time interaction, or an adjusted post-intervention between-group effect. Within-group pre–post changes alone were not counted as evidence of an intervention effect. Each study was counted no more than once within each outcome domain, regardless of the number of individual measures reported.

Antioxidant interventions were classified a priori into five primary categories for analytical assignment based on their principal hypothesized mechanism of action and the primary rationale described by the original investigators: (i) vitamins and antioxidant micronutrients; (ii) dietary polyphenols and plant-derived extracts; (iii) mitochondria-targeted or mitochondria-modulating antioxidants; (iv) nuclear factor erythroid 2-related factor 2 (Nrf2)-pathway activators and redox-responsive phytochemicals; and (v) thiol-containing or glutathione-supporting strategies. Because many antioxidants exert pleiotropic effects, each intervention was assigned to one primary category to avoid double counting. Secondary mechanisms were recorded separately and considered during mechanistic interpretation. These categories were prespecified before data synthesis and were used as a pragmatic analytical framework rather than biologically exclusive classifications. The study-level primary assignments, bases for classification, and experimentally supported secondary mechanistic actions are provided in Supplementary Table S2.

Mechanistic findings were narratively synthesized according to the number of supporting studies, evidence source, direction of reported effects, and consistency across independent studies. Evidence patterns were considered more consistent when similar outcomes were observed across multiple studies using comparable interventions or models, whereas heterogeneous findings or findings restricted to a single model were interpreted more cautiously. Mechanistic evidence was further interpreted according to the level of experimental validation. Findings based only on intervention-associated changes in pathway-related markers were classified as associative evidence, whereas evidence supported by pathway inhibition, gene silencing, or other loss-of-function approaches was classified as experimentally validated evidence. Where classification was ambiguous, assignments were discussed among the review team and finalized by consensus according to the predefined classification criteria.

## 3. Results

### 3.1. Search Results

**Figure 1:**
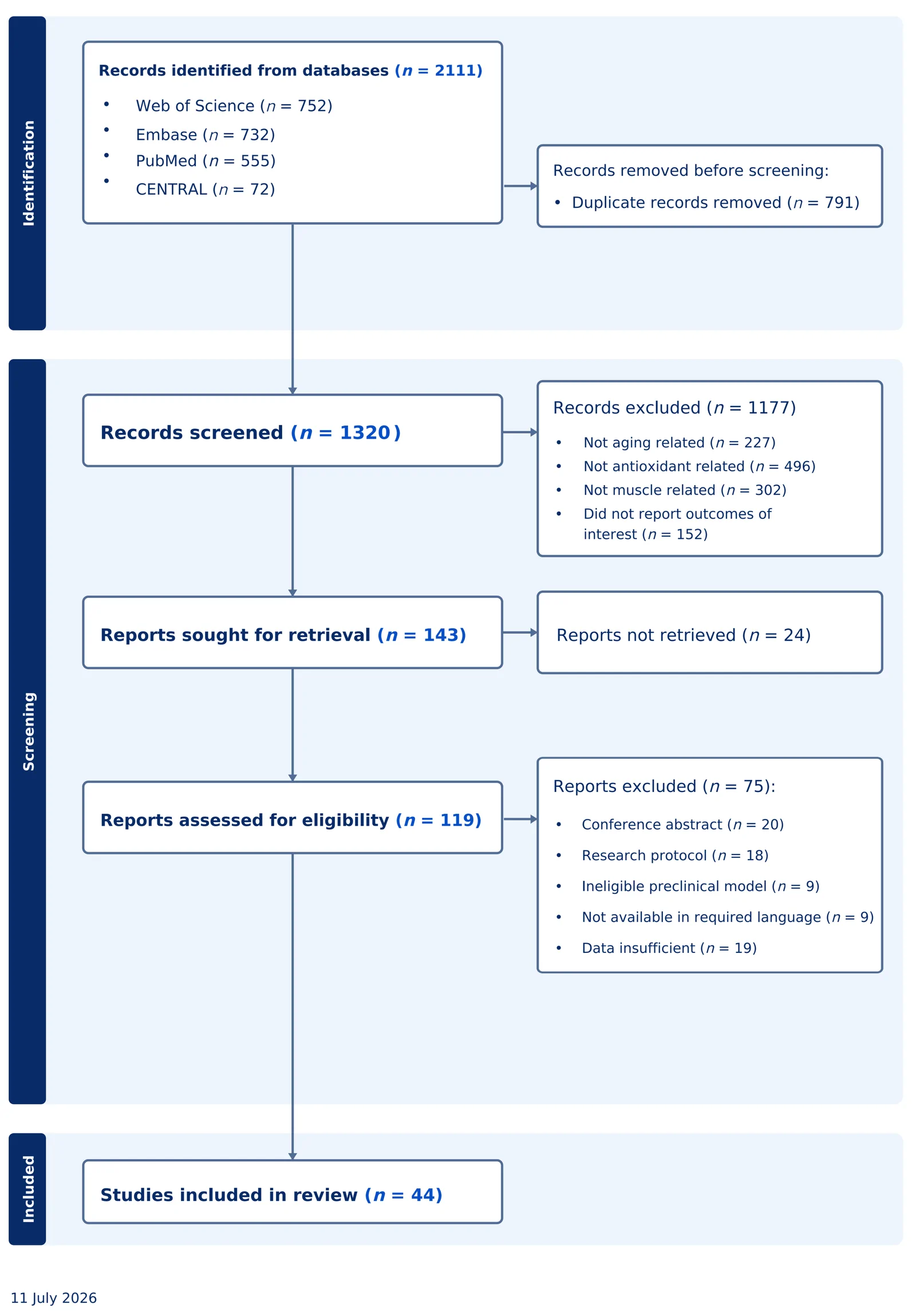
PRISMA 2020 [22] study-selection flow diagram.

The searches identified 2111 records. After deduplication, 1320 unique records remained, of which 1177 were excluded during title and abstract screening. Of 143 reports sought for retrieval, 24 could not be retrieved; 119 reports were therefore assessed for full-text eligibility. Seventy-five were excluded after full-text assessment, leaving 44 publications for inclusion. These publications contributed eight clinical trials, 26 animal datasets, and 18 cellular datasets. Eight publications contained both animal and cellular experiments and were counted once in the publication total but in both corresponding dataset categories [26,27,28,29,30,31,32,33]. Figure 1 presents the selection process.

The search identified 2111 records, of which 44 publications were included after screening and full-text eligibility assessment. Eight publications contributed both animal and cellular evidence.

### 3.2. Characteristics of Included Studies

#### 3.2.1. Characteristics of Clinical Studies

Eight clinical trials evaluated antioxidant interventions across a spectrum of sarcopenia-related and broader age-associated muscle conditions. Eight randomized controlled trials were included; seven were double-blind, whereas one was a small pilot randomized trial [34]. All used oral supplements, including vitamins, polyphenols, and multinutrient formulations. Three trials combined supplementation with exercise training [14,35,36], and one trial evaluated nutritional supplementation on top of electrical muscle stimulation applied across all study groups [37]. Interventions lasted from 4 weeks to 6 months (Table 1). Both sexes were represented, although women generally predominated. Most studies adjusted for sex without testing sex-by-treatment interactions. Only Alway et al. performed a sex-stratified analysis and found no significant difference between men and women [14]. Evidence for sex-specific responses therefore remains limited. Diagnostic criteria for sarcopenia varied across studies. Bo et al. used cutoffs consistent with AWGS 2014 [38], whereas Liu et al. applied AWGS 2019 [35]. The marine oligomeric polyphenol pilot study used sex-specific appendicular lean mass cutoffs from the Foundation for the National Institutes of Health (FNIH) Sarcopenia Project [34], while the high-flavonoid cocoa study used skeletal muscle index thresholds derived from the original European Working Group on Sarcopenia in Older People (EWGSOP) definition [39]. The remaining trials recruited older adults with low muscle mass, frailty, or mobility limitations without requiring a formal diagnosis of sarcopenia.

#### 3.2.2. Outcome Measures

Outcomes were grouped into four domains. Body composition outcomes included lean mass, appendicular skeletal muscle mass, skeletal muscle index, and body fat percentage. Lean mass, appendicular skeletal muscle mass, and skeletal muscle index were interpreted as body composition-related measures of muscle quantity, whereas body fat percentage was treated as a broader body-composition measure. These measures were not considered equivalent to tissue-level structural outcomes assessed in preclinical studies, such as muscle weight or myofiber cross-sectional area. Functional outcomes included muscle strength and physical performance measures. Muscle strength outcomes included grip strength and knee-extension force, whereas physical performance outcomes included gait speed, Timed Up and Go, and five-repetition sit-to-stand tests. Biochemical outcomes included interleukin-6 (IL-6), tumor necrosis factor-α (TNF-α), C-reactive protein, and malondialdehyde. Quality of life was assessed with instruments such as the 36-Item Short Form Health Survey (SF-36) and the EuroQol 5-Dimension (EQ-5D).

#### 3.2.3. Risk-of-Bias Assessment of Clinical Studies

Supplementary Figure S1 presents the domain-level judgments for individual clinical trials, whereas Supplementary Figure S2 summarizes the domain-level risk distribution. Seven of the eight clinical trials had a low overall risk of bias. One pilot study [34] had high risk because of missing outcomes and concerns about outcome measurement. This pilot study was therefore interpreted cautiously because of its high risk of bias and small sample size.

#### 3.2.4. Characteristics of Preclinical Studies

The preclinical evidence comprised 36 publications, representing 26 animal and 18 cellular datasets. Eight studies included both in vivo and in vitro components to support mechanistic interpretation [26,27,28,29,30,31,32,33].

Animal studies were heterogeneous in their relationship to physiological aging and were therefore interpreted according to model context. Natural aging models included conventionally aged C57BL/6 mice [27,28,41,42,43,44], Wistar rats [45,46,47], Sprague-Dawley rats [48,49,50,51], and Fischer 344 × Brown Norway F1 rats [52,53]. Some studies superimposed additional nutritional or metabolic challenges on aged animals, including antioxidant deficiency, high-sucrose feeding, or high-fat feeding [46,54,55]. Accelerated-aging or deficiency models included senescence-accelerated mouse prone 8 (SAMP8) mice [26], OXYS rats [56], and senescence marker protein-30 knockout (SMP30-KO) mice subjected to vitamin C deficiency [57]. Chemically induced aging-like models included D-galactose exposure [33,58], whereas experimentally induced muscle-atrophy or disuse models included dexamethasone administration [29,30,32] and hindlimb unloading [31]. These induced models reproduce selected oxidative, catabolic, or disuse-related features relevant to aging-related muscle decline but were not considered biologically equivalent to spontaneous age-related sarcopenia. Detailed species, age, and model characteristics are provided in Table 2.

C2C12 myoblasts were the principal cellular model. Most studies differentiated myoblasts into myotubes before inducing pathological stress. Cellular models included oxidative-stress, senescence/aging-like, and atrophy-related paradigms. Hydrogen peroxide was used to induce oxidative stress [31,59,60,61,62], whereas dexamethasone was used to model muscle-cell atrophy [29,30,32]. D-galactose was used as an aging-like or senescence-inducing stimulus [27,28,33,63], while other stressors included 2,2′-azobis(2-amidinopropane) dihydrochloride (AAPH) [64] and 7β-hydroxycholesterol [65]. One study evaluated mitochondrial respiratory responses in both myoblasts and myotubes [60], whereas another induced replicative senescence by serial passage of primary human myoblasts [66]. These cellular models were used primarily to interrogate pathways relevant to muscle aging rather than as direct equivalents of clinical sarcopenia.

Across the preclinical studies, interventions ranged from single compounds to multicomponent formulations. In animal models, monotherapies primarily utilized natural polyphenols (e.g., resveratrol, apigenin, and catechin) or essential vitamins (C and E). Several studies explored synthetic compounds designed for mitochondrial targeting, such as SkQ1 and XJB-5-131. In contrast, several investigations evaluated antioxidant blends, combining vitamins (A, E, and D) with essential trace elements (selenium and zinc) [45,46,54]. Other complex interventions included the polyphenolic mixture Taurisolo^®^ (MBMed Company, Turin, Italy) [48] and the cystine-based antioxidant (F1) [43]. Administration protocols were predominantly oral, delivered via fortified feed or gavage, with durations spanning from 4 weeks to long-term interventions of up to 10 months.

Cellular studies tested isolated compounds, including ginsenosides [59], puerarin [67], and riboflavin [63], as well as tocotrienol-rich [66] and polyphenol-rich fractions [64]. Treatments were generally applied in vitro for 24–72 h (Table 3).

#### 3.2.5. Risk-of-Bias Assessment of Animal Studies

The 26 animal datasets were assessed with the modified SYRCLE risk-of-bias tool. Supplementary Figure S3 reports per-study judgments, and Supplementary Figure S4 presents overall and selected domain-level summaries.

Overall, 21 of 26 datasets (80.8%) had unclear risk of bias, and five (19.2%) raised some concerns. None had low or high overall risk. Selective reporting, incomplete outcomes, and other biases were generally rated low risk. Sequence generation was adequate in 14 datasets. Blinding, housing, baseline comparability, and random outcome assessment were commonly unclear. Animal evidence was therefore characterized by substantial reporting uncertainty, particularly for blinding, housing, baseline comparability, and random outcome assessment, and preclinical mechanistic conclusions should be interpreted cautiously.

#### 3.2.6. Quality Assessment of Cellular Studies

**Figure 2:**
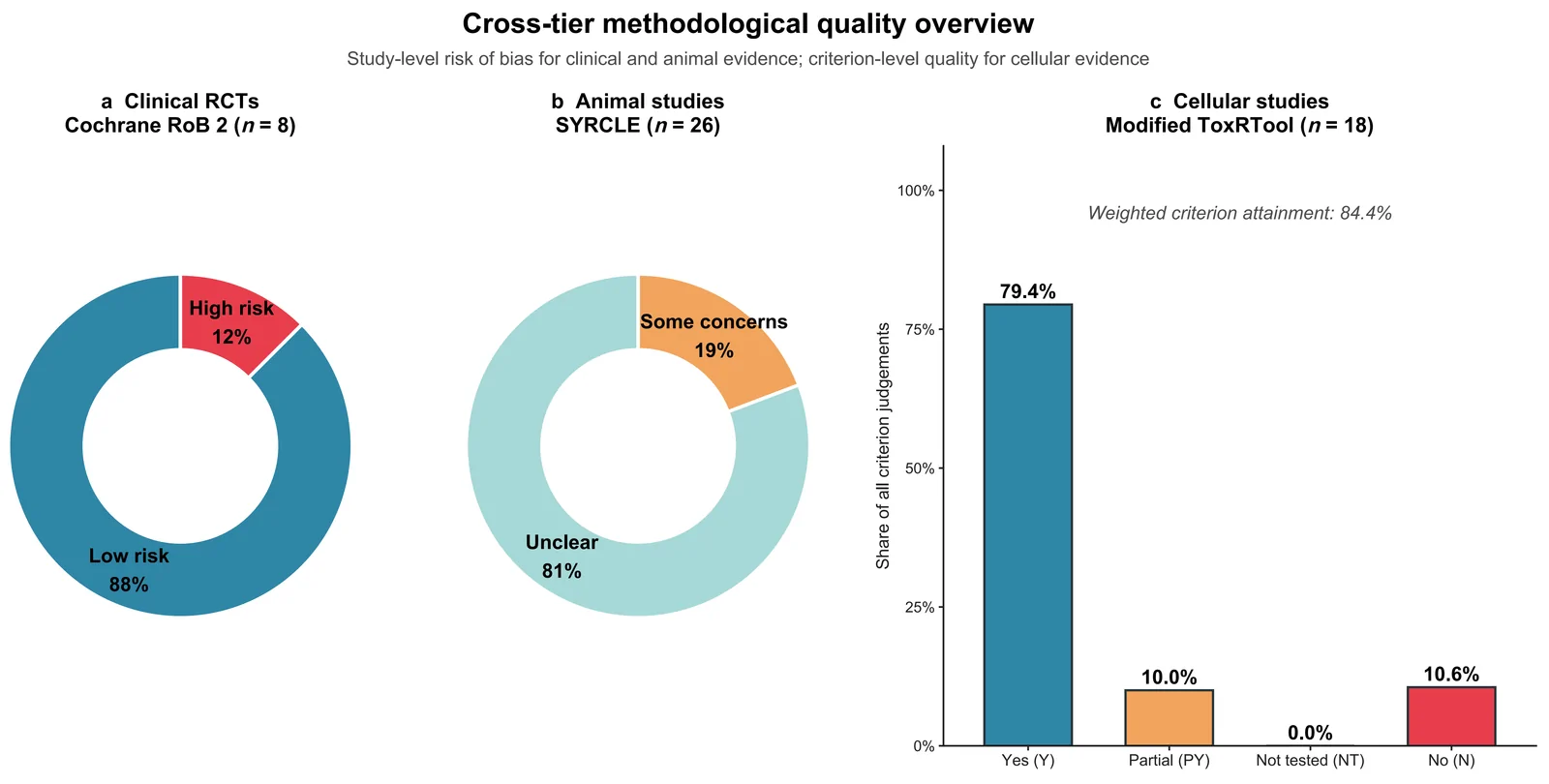
Evidence landscape and methodological quality. (**a**) Overall risk of bias in clinical trials. (**b**) Overall risk of bias in animal studies. (**c**) Overall methodological quality of cellular studies. The three panels use different appraisal frameworks and are not directly comparable. Abbreviations: RCTs, randomized controlled trials; RoB 2, Cochrane Risk of Bias 2; SYRCLE, Systematic Review Centre for Laboratory animal Experimentation; ToxRTool, Toxicological data Reliability Assessment Tool; Y, yes; PY, partly yes; NT, not tested; N, no.

The 18 cellular datasets were evaluated with a modified ToxRTool framework for in vitro antioxidant research. Supplementary Figure S5 presents the criterion-level summary, whereas Supplementary Figure S6 presents the detailed study-level judgments. The methodological quality assessment across clinical, animal, and cellular evidence tiers is summarized in Figure 2.

Of 180 assessed criteria, 143 (79.4%) were fully met, 18 (10.0%) were partly met, and 19 (10.6%) were unmet. The weighted attainment was 84.4%. The three panels represent different appraisal frameworks and should not be directly compared. The weighted attainment shown for cellular studies reflects checklist fulfillment within the modified in vitro assessment framework. Objectives, test systems, controls, outcomes, and statistical methods were usually well described. Sixteen datasets reported adequate replication. Reporting of substance identification and concentration justification showed greater variability across studies. No study reported blinded outcome assessment, which particularly affects semiquantitative analyses such as immunofluorescence. Cellular findings therefore support target engagement and proof of concept but remain hypothesis-generating for whole-organism physiology.

### 3.3. Results by Antioxidant Class

Results are presented according to the five prespecified primary categories defined in the Methods. These categories were used only for analytical assignment; secondary mechanisms were cross-referenced without double counting. Primary assignments are shown in Table 4, with the rationale for classification and supported secondary mechanisms provided in Supplementary Table S2.

**Table 4:** **Analytical Classification and Evidence Distribution of Included Antioxidant Interventions.**

Primary Category	Defining Basis	Evidence Source	Included Interventions
**Vitamins and antioxidant micronutrients**	Essential vitamins, vitamin E isoforms, and trace-element or multinutrient formulations primarily used to support antioxidant defense.	Clinical	Vitamins C/E; whey protein with vitamins D/E
		Animal	Leucine-enriched whey with antioxidants; rutin–vitamin–trace-element mixtures; whey/chamomile/vitamin formulation; vitamin C; tocotrienol-rich fraction
		Cell	Riboflavin; vitamin D metabolites; tocotrienol-rich fraction/α-tocopherol with NAC
**Dietary polyphenols and plant-derived extracts**	Polyphenols, flavonoids, phenolic acids, polysaccharides, and plant or algal extracts classified mainly by their phytochemical identity and natural origin.	Clinical	Resveratrol; marine oligomeric polyphenols; epicatechin-enriched green tea extract; flavonoid-rich cocoa; Schisandra chinensis extract; rutin/curcumin-containing formulation
		Animal	Oligonol®; Polygonatum sibiricum polysaccharide; ferulic acid; lotus leaf extract; amentoflavone; ginsenoside Ro; apigenin; resveratrol; Taurisolo®; Chlorella vulgaris; nobiletin; dark-chocolate flavanols/(−)-epicatechin; (+)-epicatechin
		Cell	Oligonol®; Polygonatum sibiricum polysaccharide; ferulic acid; quercetin 3-O-β-glucuronide; amentoflavone; ginsenoside Ro; milk thistle seed oil; polyphenol-rich plant extract; puerarin
**Mitochondria-targeted or mitochondria-modulating antioxidants**	Compounds designed to accumulate in mitochondria, modulate mitochondrial redox function, or deliver antioxidant activity to oxidatively injured muscle.	Animal	PQQ; edaravone-loaded human serum albumin nanoparticles; XJB-5-131; SkQ1
		Cell	PQQ; edaravone-loaded human serum albumin nanoparticles
**Nrf2 pathway activators and redox-responsive phytochemicals**	Phytochemicals primarily investigated through Nrf2/HO-1 or closely related redox-responsive, ROS-dependent, and mitochondrial stress pathways.	Cell	Ginsenoside Rb1; verbascoside; platycodin D; trans-cinnamaldehyde
**Thiol-containing and glutathione-supporting strategies**	Thiol-containing compounds or precursor formulations primarily designed to increase glutathione synthesis or glutathione-dependent redox capacity.	Animal	F1 cystine-based glutathione precursor

**Note**: Each intervention was assigned to one primary category for analytical assignment according to the dominant mechanistic hypothesis stated by the original authors. Pleiotropic and multicomponent interventions were classified once to avoid double counting; secondary mechanisms were recorded separately and were not counted as separate categories. Study-level rationales and experimentally supported secondary actions are reported in Supplementary Table S2. **Evidence coverage:** Eight publications contributed both animal and cellular evidence and are displayed in both evidence-source rows but counted once among the 44 unique included studies. **Abbreviations:** NAC, N-acetylcysteine; Nrf2, nuclear factor erythroid 2-related factor 2; PQQ, pyrroloquinoline quinone; ROS, reactive oxygen species.

#### 3.3.1. Vitamins and Antioxidant Micronutrients

Two clinical studies evaluated vitamin or antioxidant-micronutrient interventions, and both reported improvements in muscle strength and at least one body composition-related muscle quantity measure [35,38]. However, these benefits occurred in the context of resistance training or protein-based nutritional support, and neither study demonstrated a clear additional benefit in physical performance. In the trial of vitamins C/E combined with resistance training, handgrip strength and knee-extension strength increased by approximately 2.5 kg and 1.3 kg more, respectively, than with resistance training alone. Skeletal muscle index and arm lean mass also showed additional increases of 0.29 kg/m^2^ and 0.37 kg, respectively, whereas total lean mass, leg lean mass, and mobility-related outcomes such as the Timed Up and Go test did not show clear additional improvement [35]. A 6-month trial of whey protein combined with vitamins D/E showed a similar outcome pattern: relative skeletal muscle index increased by approximately 0.18 kg/m^2^ and handgrip strength by approximately 2.7 kg, whereas appendicular skeletal muscle mass and mobility-related outcomes did not improve consistently [38].

Animal evidence included a vitamin C deficiency model, natural aging models, and naturally aged animals exposed to additional nutritional or metabolic challenges. Among these, the vitamin C deficiency model provided the clearest structural and functional evidence. In SMP30-KO mice, vitamin C deficiency reduced gastrocnemius and soleus weights to 74–83% of control values, decreased grip strength by 62% at week 16, and reduced endurance by more than 50%; these changes were restored after vitamin C repletion [57]. By contrast, in non-deficient or standard aged models, antioxidant mixtures more often altered redox or protein-metabolic indices, with limited recovery of muscle mass. A diet containing rutin, vitamins A/E, zinc and selenium restored leucine-stimulated protein synthesis in aged rats, but did not increase the weights of major hindlimb muscles [45]. In a sucrose-feeding model, lean-mass loss increased from 5.4% to 8.1%; antioxidant supplementation increased plasma antioxidant capacity and muscle protein concentration, but did not prevent the loss of lean mass or muscle mass [46]. A tocotrienol-rich fraction administered at 60 mg/kg/day for 3 months increased antioxidant enzyme activity, reduced lipid peroxidation and inflammation, and improved histological features, without clear evidence of restored muscle mass [50].

Cellular evidence further supported the effects of this category on redox and mitochondrial endpoints. D-galactose treatment increased the proportion of senescence-associated β-galactosidase (SA-β-gal)-positive C2C12 cells from 8.7% to 76.3%, whereas riboflavin reduced this proportion to 29.4%. D-galactose reduced adenosine triphosphate (ATP) levels by approximately 25%, and riboflavin restored ATP levels, together with improvements in myotube width and redox-related markers [63].

Overall, the benefits in this category were most apparent when treatment corrected a deficiency or accompanied resistance training or protein-based nutritional support.

#### 3.3.2. Dietary Polyphenols and Plant-Derived Extracts

Dietary polyphenols and plant-derived extracts represented the largest and most heterogeneous intervention category. Among the six clinical studies of polyphenol or plant-derived interventions, five reported a significant comparative benefit in at least one muscle strength or power measure [14,36,37,39,40], and two reported a benefit in at least one physical-performance measure [34,39]. None demonstrated a significant comparative increase in body composition-related muscle quantity. When used as an exercise adjunct, resveratrol increased knee-extension peak torque by approximately 8% and average power by approximately 14%, but did not further improve lean mass compared with exercise alone [14]. Green tea extract improved right handgrip strength and right leg flexor peak torque; arm muscle mass decreased by approximately 80 g over 12 weeks in the control group, whereas a comparable decline was not observed in the intervention group. However, no clear between-group differences were found in whole-body or regional muscle mass [40]. In the initial cocoa cohort, skeletal muscle index increased within the flavanol group by approximately 0.8 kg/m^2^, but a significant between-group difference was not demonstrated. The average Timed Up and Go test time also improved within the flavanol group by approximately 0.7 s. In the separate follow-up cohort, flavanol-rich cocoa increased 6-min walking distance by approximately 35 m and improved several other mobility-related measures relative to non-flavanol cocoa, whereas handgrip findings differed between the two cohorts [39]. Schisandra extract and combined nutritional formulas containing curcumin or rutin mainly improved knee-extension strength and gait speed, with limited effects on lean mass or appendicular skeletal muscle mass [36,37].

In animal studies, polyphenol effects depended strongly on the specific compound and model used. Apigenin was one of the few interventions that improved both structural and functional outcomes in a natural aging model. Treatment at 50 mg/kg/day from 16 to 25 months of age increased hindlimb muscle weight and myofiber cross-sectional area, improved grip strength and running distance, and reduced frailty index scores [41]. In contrast, dark chocolate flavanols and (–)-epicatechin improved forelimb grip strength and inverted-screen performance, but did not change gastrocnemius weight [55]. Resveratrol studies in animals also showed that functional changes did not necessarily parallel changes in muscle mass: long-term supplementation reduced oxidative stress but did not preserve the weights of major hindlimb muscles, while another 6-month study improved ex vivo fatigue resistance, but tibialis anterior fiber cross-sectional area decreased by 8.6%, and treadmill endurance did not improve [42,44]. In 23-month-old rats, 8 weeks of (+)-epicatechin increased skeletal-muscle nicotinamide adenine dinucleotide (NAD^+^) by approximately 80%, increased NAD^+^/reduced nicotinamide adenine dinucleotide (NADH) ratio by more than 100%, increased complex I activity by approximately 100%, increased citrate synthase activity by more than 200%, and increased ATP content by approximately 90% [51]. Taurisolo^®^ improved motor performance and antioxidant capacity in aged animals, but muscle mass was not measured [48].

Structural protection was reported mainly in experimentally induced atrophy or aging-like models. Lotus leaf extract, ferulic acid, amentoflavone and ginsenoside Ro improved myotube diameter, myofiber cross-sectional area, muscle mass or motor performance in dexamethasone, D-galactose or related stress models, accompanied by changes in acyl-CoA oxidase 1 (ACOX1)/muscle RING finger 1 (MuRF1), myostatin (MSTN)-SMAD signaling or mitochondrial-inflammatory pathways [29,30,32,33]. Among these, ginsenoside Ro increased gastrocnemius fiber cross-sectional area in D-galactose-treated mice from 1209.34 ± 89.76 μm^2^ to 1686.56 ± 90.68 μm^2^, and restored running time, running distance, maximum speed and grip strength [33]. Nobiletin likewise improved hindlimb muscle mass, lean mass, myofiber size, and skeletal-muscle function in D-galactose-treated mice, accompanied by activation of protein kinase B (Akt)/mechanistic target of rapamycin (mTOR)-related protein-synthesis signaling and suppression of forkhead box O3a (FoxO3a)/muscle atrophy F-box (MAFbx)/MuRF1-related protein-degradation signaling [58]. Because this study used a chemically induced aging-like model, its findings were interpreted as protection against D-galactose-associated atrophy rather than evidence from natural aging. Cellular studies mainly provided evidence for stress resistance, myogenic differentiation and proteostasis, with ferulic acid, amentoflavone and puerarin affecting ROS, MuRF1, myotube formation or myogenic marker expression [29,32,67]. These findings reflect protection against experimentally induced catabolic or aging-like stress and were considered separately from evidence obtained in natural aging models.

Simultaneous structural and functional improvement was uncommon in natural aging models, whereas structural protection was reported more often in induced atrophy or aging-like models.

#### 3.3.3. Mitochondria-Targeted or Mitochondria-Modulating Antioxidants

No eligible clinical trial evaluated a mitochondria-targeted or mitochondria-modulating antioxidant in older adults with sarcopenia; evidence for this category was entirely preclinical. Representative interventions included pyrroloquinoline quinone (PQQ), SkQ1 and XJB-5-131. secreted protein acidic and rich in cysteine (SPARC)-guided edaravone nanoparticles were also included in this category because they involved targeted antioxidant delivery and mitochondrial redox-related outcomes, although the delivery strategy itself was not strictly mitochondria-targeted. Outcomes in this category focused on mitochondrial structure, respiratory-chain function and single-fiber contractile performance.

PQQ had both animal and cellular evidence. In aged mice, PQQ increased fast-twitch and total fiber cross-sectional area in the soleus, but did not change fiber number. Regarding physical performance outcomes, PQQ mainly improved pole test performance, whereas hanging time did not show a clear benefit [27]. In C2C12 cells, 50–100 nM PQQ reduced the 2.6-fold D-galactose-induced increase in ROS to near-control levels, and increased the fusion index from approximately 10% to approximately 40% [27]. SkQ1 mainly improved mitochondrial ultrastructure, but did not report muscle weight, grip strength, endurance or contractile force [56]. XJB-5-131 provided clearer functional data. In aged rats treated with 3 mg/kg three times per week for 4 weeks, gastrocnemius single-fiber maximal unloaded shortening velocity increased by 35% and absolute power by 58%; however, gastrocnemius fiber cross-sectional area and most muscle weights did not increase [52]. SPARC-guided edaravone nanoparticles preserved tibialis anterior and gastrocnemius weights in a 14-day hindlimb-unloading model and improved treadmill running distance; free edaravone did not show the same effect [31]. Because this study used an acute disuse model in young mice, its findings were interpreted as evidence of protection against disuse-induced atrophy rather than as evidence from physiological aging.

Existing results point more toward improvements in mitochondrial integrity, respiratory-chain function, and single-fiber contractile performance than toward increases in muscle mass. The absence of clinical data therefore represents a clear translational gap for strategies designed to target mitochondrial dysfunction.

#### 3.3.4. Nrf2 Pathway Activators and Redox-Responsive Phytochemicals

No eligible clinical intervention was primarily assigned to the Nrf2-pathway category, and no included clinical trial directly measured Nrf2 target engagement in human skeletal muscle. Evidence for this category mainly came from C2C12 cells and a small number of animal studies. In H_2_O_2_-treated C2C12 myoblasts, platycodin D reduced ROS and apoptosis and protected mitochondrial membrane potential; Nrf2 small interfering RNA (siRNA) weakened its Nrf2/heme oxygenase-1 (HO-1) activation and cytoprotective effects [61]. Verbascoside and ginsenoside Rb1 were associated with improved mitochondrial respiration, cell survival, and bioenergetic indices, together with reduced oxidative stress [59,60]. Although apigenin and Taurisolo^®^ were primarily classified as polyphenol-related interventions, they also provided cross-referenced evidence for endogenous antioxidant defense. Apigenin improved muscle structure, function and oxidative-damage markers in naturally aged mice, whereas Taurisolo^®^ improved rotarod performance and antioxidant enzyme activity in aged rats [41,48].

Current evidence is therefore preclinical and primarily supports cytoprotective and endogenous antioxidant responses.

#### 3.3.5. Thiol-Containing and Glutathione-Supporting Strategies

Evidence primarily assigned to this category came from the cystine-based formulation F1 [43]. Glutathione-related findings reported in vitamin or multinutrient studies [35,45,46] were considered as secondary cross-class mechanisms and were not reassigned to this primary category. Human evidence was indirect because glutathione-related changes were reported in multinutrient studies rather than in interventions assigned primarily to this category. In the vitamins C/E plus resistance-training trial, reduced glutathione (GSH) and the GSH/oxidized glutathione (GSSG) ratio increased while GSSG and malondialdehyde (MDA) decreased, but the contribution of glutathione-related mechanisms could not be separated from the effects of training and vitamin supplementation [35]. F1 was the main preclinical intervention in this category.

In animals, F1 showed structural protection and anti-apoptotic signaling, but functional outcomes are still lacking. As a glutathione precursor composed of L-cystine, glycine, selenomethionine and L-glutamine, F1 treatment for 6 months in aged mice increased gastrocnemius weight and myofiber cross-sectional area toward levels observed in young mice [43]. Aging reduced gastrocnemius weight by 17.3%; F1 increased relative gastrocnemius weight from 0.35 to 0.44 g per 100 g body weight, whereas the value in young mice was 0.62 g per 100 g body weight. F1 also restored the GSH/GSSG ratio, reduced 4-hydroxynonenal (4-HNE), increased mitochondrial superoxide dismutase 2 (SOD2), and reduced terminal deoxynucleotidyl transferase dUTP nick-end labeling (TUNEL)-positive muscle cells from approximately 11% to nearly 0%. This study did not report muscle strength or physical-performance outcomes. Selenium- and zinc-containing antioxidant mixtures restored leucine-stimulated protein synthesis or increased antioxidant capacity, but did not consistently prevent the loss of lean mass or muscle mass [45,46].

#### 3.3.6. Cross-Class Outcome Patterns

Among clinical studies and natural-aging or deficiency models, structural benefits, including improvements in body composition-based muscle quantity measures or tissue-level structural outcomes (such as myofiber cross-sectional area), were most evident in four contexts: (i) vitamin deficiency correction, where the intervention addressed an overt nutritional deficit [57]; (ii) exercise or nutritional co-interventions, where the antioxidant served as an adjunct to resistance training or protein supplementation [35,38]; (iii) apigenin treatment in naturally aged mice, which was the sole polyphenol demonstrating simultaneous structural and functional improvement in a natural aging model [41]; and (iv) F1 supplementation, which partially restored gastrocnemius weight and increased myofiber cross-sectional area toward young-animal levels [43]. Structural preservation was reported particularly in chemically induced aging-like or experimentally induced atrophy/disuse models, including dexamethasone, D-galactose, and hindlimb unloading paradigms, whereas evidence from natural aging models was more limited. These findings from experimentally induced models were interpreted as evidence of protection against specific catabolic, oxidative, or disuse-related processes rather than direct evidence of efficacy against age-related sarcopenia. In contrast, mitochondrial and redox-related indices were often reported to change without measurable hypertrophy, particularly among mitochondria-targeted antioxidants and several polyphenol interventions. At the study level, seven of the eight clinical studies reported a significant comparative benefit in at least one muscle strength or muscle power measure [14,35,36,37,38,39,40]. Two studies reported a benefit in at least one physical-performance measure [34,39], corresponding to two of the six studies that assessed mobility- or balance-related performance. In contrast, only two of the eight studies demonstrated a significant comparative improvement in body composition-related muscle quantity [35,38]. Accordingly, six studies reported improvement in at least one selected functional outcome without a corresponding comparative increase in body composition-related muscle quantity [14,34,36,37,39,40]. These counts indicate study-level support for at least one outcome and should not be interpreted as consistent improvement across all measures within each study.

Selected functional and structural outcomes therefore did not always change in parallel. In the clinical studies, this pattern was driven mainly by strength or power, whereas evidence for physical performance was limited. Given the limited number and heterogeneity of clinical trials, this pattern should be considered hypothesis-generating rather than an established class-wide effect of antioxidant treatment.

#### 3.3.7. Trials with Versus Without Exercise-Based Co-Interventions

Four of the eight clinical trials incorporated an exercise-based co-intervention: three combined supplementation with exercise training [14,35,36], whereas one applied electrical muscle stimulation to all study groups [37]. In these studies, the observed effects cannot be attributed solely to antioxidant supplementation. Vitamins C/E combined with resistance training produced additional gains of approximately 2.5 kg in handgrip strength and 1.3 kg in knee-extension strength relative to resistance training alone and also improved selected body composition indices, although total lean mass, leg lean mass, and physical-performance outcomes did not differ clearly between groups [35]. Resveratrol plus exercise increased knee-extension peak torque by approximately 8% and average power by approximately 14% without a parallel increase in lean mass [14]. Schisandra extract combined with low-intensity exercise improved knee-extension strength without a significant increase in muscle mass [36]. In the electrical-muscle-stimulation trial, whey protein, omega-3 fatty acids, and polyphenols increased knee-extension strength by approximately 13% relative to the carbohydrate control on top of electrical muscle stimulation (EMS), without a clear comparative improvement in muscle size [37]. Thus, three of these four studies reported strength or power-related benefits without a comparative increase in body composition-related muscle quantity [14,36,37], whereas the vitamins C/E trial reported improvements in both strength and selected body composition indices [35].

Four trials did not include an exercise-based co-intervention [34,38,39,40]. Whey protein combined with vitamins D/E increased relative skeletal muscle index and handgrip strength, although appendicular skeletal muscle mass and mobility outcomes did not improve consistently [38]. High-flavanol cocoa improved selected mobility outcomes, but between-group changes in muscle quantity and handgrip strength were inconsistent across cohorts [39]. Green tea extract improved selected strength outcomes without a clear between-group increase in whole-body or regional muscle mass [40]. The marine-polyphenol pilot study showed no clear comparative benefit for body composition, grip strength, Timed Up and Go, or chair-rise performance, although the one-leg stand test showed a marginal group-by-time interaction [34]. Separating trials according to exercise-based co-intervention clarifies the intervention context, but it does not isolate the independent contribution of antioxidant compounds from other nutritional components such as whey protein, vitamin D, or omega-3 fatty acids.

### 3.4. Molecular Mechanisms of Antioxidant Action

**Figure 3:**
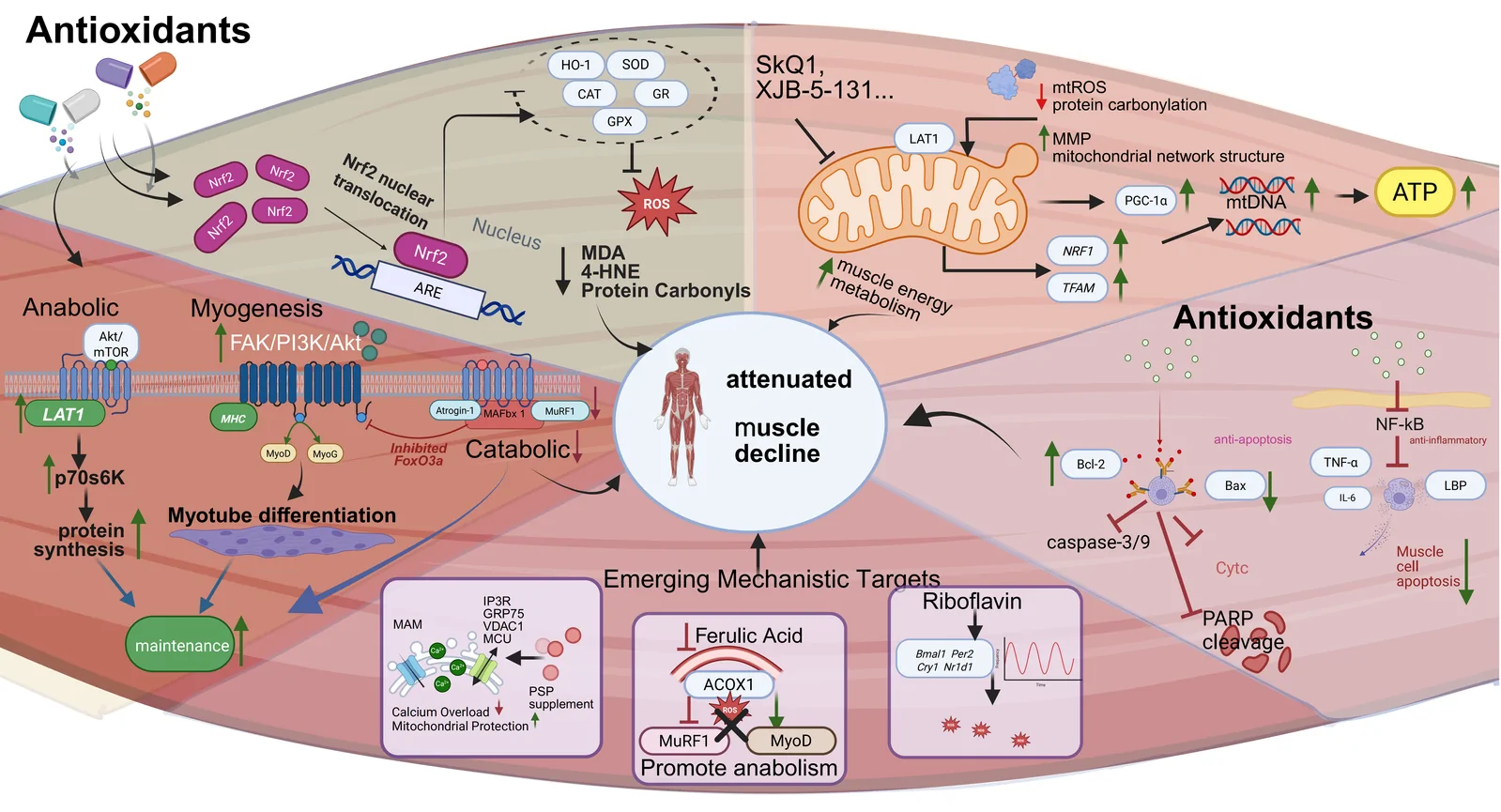
Multi-target mechanisms of antioxidants against aging-related muscle decline. Abbreviations: ROS, reactive oxygen species; Nrf2, nuclear factor erythroid 2-related factor 2; HO-1, heme oxygenase-1; PGC-1α, peroxisome proliferator-activated receptor gamma coactivator 1-alpha; NRF1, nuclear respiratory factor 1; TFAM, mitochondrial transcription factor A; NAD+/NADH, nicotinamide adenine dinucleotide redox pair; ATP, adenosine triphosphate; AKT, protein kinase B; mTOR, mechanistic target of rapamycin; FoxO, forkhead box O; MuRF1, muscle RING-finger protein-1; LC3, microtubule-associated protein 1 light chain 3; PINK1, PTEN-induced kinase 1.

Mechanistic evidence was derived mainly from animal and cellular studies. Because the included clinical trials rarely assessed redox target engagement directly in skeletal muscle, these pathways should be interpreted as preclinical mechanisms associated with antioxidant interventions rather than confirmed mechanisms of clinical efficacy. We therefore distinguished experimentally validated pathways from associations based mainly on changes in molecular or functional markers. The multi-target mechanisms through which antioxidant interventions may influence these interconnected processes are summarized in Figure 3.

#### 3.4.1. Mitochondrial Function and Bioenergetic Efficiency

Mitochondrial function represented one of the most frequently investigated mechanistic themes. XJB-5-131 increased single-fiber shortening velocity and power in aged rats without increasing muscle weight or myofiber cross-sectional area [52]. Mechanistically, this change was accompanied by increased activities of respiratory-chain complexes I, III and IV, whereas citrate synthase activity and electron transport chain (ETC) supercomplex abundance were unchanged. This pattern suggests improved mitochondrial functional efficiency rather than increased mitochondrial mass.

Other mitochondria-modulating interventions pointed in the same direction. SkQ1 mainly preserved mitochondrial ultrastructure in OXYS accelerated-aging rats, but did not assess muscle mass or functional outcomes [56]. In D-galactose-treated C2C12 cells, PQQ was associated with reduced ROS, increased mitochondrial number, an improved fusion index, and changes in peroxisome proliferator-activated receptor gamma coactivator 1-alpha (PGC-1α)/mitochondrial transcription factor A (TFAM)- and NAD^+^-related markers; however, evidence of skeletal-muscle target engagement in vivo was relatively weak [27]. Non-mitochondria-targeted antioxidants such as apigenin, riboflavin, verbascoside and (+)-epicatechin also repeatedly affected PGC-1α/nuclear respiratory factor 1 (NRF1)/TFAM, ATP, oxidative phosphorylation (OXPHOS) proteins, mitochondrial respiratory reserve or NAD^+^/sirtuin 1 (SIRT1)/PGC-1α-related metabolism [41,51,60,63]. Mitochondrial alterations therefore represented one of the most consistently reported mechanistic patterns across antioxidant categories; however, these findings were predominantly associative because none of the studies directly tested whether mitochondrial improvement was required for the observed muscle benefits.

#### 3.4.2. Endogenous Antioxidant Defense and Cell Survival

Multiple studies showed that antioxidant interventions can enhance endogenous defense systems, although pathway validation varied. Platycodin D provided experimentally validated evidence for the involvement of the Nrf2/HO-1 pathway in a cellular model. H_2_O_2_ reduced C2C12 myoblast viability and markedly increased ROS, while platycodin D reduced ROS, protected mitochondrial membrane potential and decreased apoptosis. Nrf2 siRNA weakened its Nrf2/HO-1 activation and cytoprotective effects [61]. Verbascoside was also accompanied by Nrf2/HO-1 activation and improved mitochondrial respiratory reserve, but lacked Nrf2 loss-of-function validation, making it more appropriate to interpret as associative mechanistic evidence [60]. Thus, direct experimental validation of Nrf2/HO-1 dependence was provided by the platycodin D study, whereas the findings for verbascoside and other interventions were associative because they were based on pathway-related marker changes without loss-of-function validation.

In animal studies, Taurisolo^®^, apigenin, tocotrienol-rich fraction (TRF) and F1 all improved antioxidant enzyme activity, GSH/GSSG balance, lipid peroxidation or protein carbonylation [41,43,48,50]. Among these, F1 improved GSH/GSSG, 4-HNE, mitochondrial SOD2 and TUNEL-positive muscle cells [43]. Across these studies, antioxidant interventions were commonly accompanied by enhanced endogenous defense and improved cell survival, although most studies have not shown that these pathways are required for muscle protection.

#### 3.4.3. Proteostasis, Anti-Atrophy Signaling and Myogenic Repair

Antioxidant interventions also affected protein synthesis, protein degradation and myogenic repair. A diet containing rutin, vitamins A/E, zinc and selenium restored leucine-stimulated protein synthesis in aged rats, but did not increase the weights of major hindlimb muscles [45]. Similarly, in a sucrose-feeding model, antioxidant supplementation increased muscle protein concentration and plasma antioxidant capacity, but did not prevent lean-mass or muscle-mass loss [46]. These findings indicate that improvements in protein-metabolic signaling do not necessarily translate into measurable hypertrophy.

Polyphenols and plant-derived compounds more frequently involved anti-atrophy pathways. Among these interventions, direct pathway validation was reported in two cellular studies. For oligonol combined with branched-chain amino acids, the L-type amino acid transporter 1 (LAT1) inhibitor JPH203 reversed the synergistic activation of mTOR/70-kDa ribosomal protein S6 kinase (p70S6K), while LAT1 silencing reduced branched-chain amino acids (BCAA)-related signaling [26]. In the puerarin study, inhibition of focal adhesion kinase (FAK) abolished puerarin-induced myoblast migration, whereas phosphoinositide 3-kinase (PI3K) inhibition suppressed PI3K/AKT activation and the associated enhancement of myoblast fusion and differentiation [67]. These findings support pathway dependence for specific cellular endpoints, but not for in vivo sarcopenia outcomes.

Dark chocolate flavanols and (-)-epicatechin improved functional performance and were accompanied by changes in the follistatin/myostatin ratio, myocyte enhancer factor 2A (MEF2A), forkhead box O1A (FOXO1A) and MuRF1, but did not increase gastrocnemius weight [55]. Lotus leaf extract, quercetin-3-O-glucoside (Q3G), ferulic acid and amentoflavone were associated with Akt/mTOR, Atrogin-1/MuRF1, ACOX1 and MSTN-SMAD pathways, respectively [29,30,32]. Nobiletin was also accompanied by increased Akt/mTOR-related signaling and reduced FoxO3a/MAFbx/MuRF1-related protein-degradation signaling in D-galactose-treated mice [58]. Because pathway inhibition or loss-of-function experiments were not performed, these findings were classified as associative rather than experimentally validated evidence. Except for the pathway-inhibition experiments involving oligonol and puerarin, most findings in this subsection were associative and were based on changes in Akt/mTOR, FoxO, MuRF1, ACOX1, MSTN-SMAD, or related markers.

#### 3.4.4. Inflammation, Apoptosis and Mitochondrial Quality Control

Inflammation, apoptosis and mitochondrial quality control frequently changed together with redox or mitochondrial endpoints. PQQ, ginsenoside Ro and Taurisolo^®^ reduced inflammation-related markers in animal models and were accompanied by improvements in antioxidant capacity or muscle performance [27,33,48]. In clinical studies, changes in inflammatory markers were inconsistent: vitamins C/E combined with resistance training reduced IL-6, whereas Schisandra extract improved knee-extension torque without clear changes in inflammatory or oxidative-stress markers [35,36]. Inflammation modulation may therefore contribute to some antioxidant responses, but it cannot explain all functional benefits.

Evidence related to apoptosis mainly came from animal and cellular studies. One cellular study provided direct experimental validation of redox-sensitive atrophic signaling. In calcitriol-treated C2C12 myotubes, N-acetylcysteine (NAC) and protein kinase C (PKC) inhibition attenuated ROS accumulation, mitochondrial membrane-potential loss, atrogene induction, and myotube atrophy, while c-Jun N-terminal kinase (JNK) inhibition prevented the reduction in myotube diameter [68]. These findings support a PKC–ROS–JNK-dependent mechanism for calcitriol-induced cellular atrophy, although they do not establish a protective antioxidant mechanism in aged muscle.

Platycodin D and trans-cinnamaldehyde both reduced H_2_O_2_-induced mitochondrial apoptosis-related changes [61,62]. F1 markedly reduced TUNEL-positive muscle cells in aged mice and was accompanied by changes in JNK, AMP-activated protein kinase (AMPK) and Akt-related signaling [43]. PQQ, apigenin and platycodin D also affected markers of mitochondrial quality control, including Parkin, BCL2-interacting protein 3 (BNIP3), p62 and microtubule-associated protein 1 light chain 3-II/I (LC3-II/I) [27,41,61]. These findings provided associative evidence for altered mitochondrial quality control. Because the treatment effects were not tested using autophagy- or mitophagy-specific inhibition, gene silencing, or rescue experiments, changes in Parkin, BNIP3, p62, or LC3-II/I should not be interpreted as evidence that these pathways were required for muscle protection.

#### 3.4.5. Emerging Mechanisms Beyond Canonical ROS Scavenging

Several studies proposed mechanisms beyond direct ROS scavenging. Polygonatum polysaccharide was linked to mitochondria-associated membrane (MAM) and calcium-homeostasis regulation, involving changes in calcium-regulatory proteins such as inositol 1,4,5-trisphosphate receptor (IP3R) and voltage-dependent anion channel 1 (VDAC1) [28]. Ferulic acid was associated with reduced peroxisomal ACOX1 activity and downstream redox- and atrophy-related changes. Although pharmacological ACOX1 inhibition produced similar effects, the study did not demonstrate that ACOX1 was required for the response to ferulic acid [29]. In D-galactose-treated C2C12 cells, riboflavin restored ATP, myotube width and oscillation of core clock genes, suggesting a possible link between mitochondrial function and circadian rhythm [63]. PQQ and ginsenoside Ro also suggested that the gut barrier and gut-muscle axis may participate in muscle outcomes, but current evidence remains mainly model-based or associative [27,33].

These emerging mechanisms broaden the explanatory framework for antioxidant action, but the evidence remains limited. Based on the available data, mitochondrial function, endogenous antioxidant defense, proteostasis, and cell-survival pathways were the most frequently reported mechanistic themes, although most were supported by associative rather than experimentally validated evidence.

## 4. Discussion

This synthesis suggested a possible, although non-uniform, dissociation between selected functional and structural outcomes. Antioxidant interventions were associated with improvements in selected functional outcomes even when parallel gains in body composition-related muscle quantity or tissue-level structural measures were not observed. In clinical studies, this pattern was driven mainly by strength- or power-related outcomes: seven of eight studies reported a benefit in at least one such measure, whereas support was more limited for physical performance and body composition-related muscle quantity. Selected preclinical studies showed a similar but model-dependent pattern, with functional or contractile improvements not always accompanied by increases in muscle weight or myofiber size. This distinction is important because muscle strength and physical performance are related but non-interchangeable domains, reflecting force-generating capacity and integrated task performance, respectively.

Organizing the evidence by antioxidant category further showed that antioxidants should not be treated as a homogeneous intervention class. The categories differed in evidence source, outcome pattern, and mechanistic emphasis. In the two clinical studies of vitamins and antioxidant micronutrients, improvements in strength and selected muscle quantity measures occurred in the context of resistance training or protein-based nutritional support [35,38], whereas correction of vitamin C deficiency provided the clearest preclinical evidence in this category [57]. Among the six clinical studies of polyphenol or plant-derived interventions, five reported a benefit in at least one strength or power measure and two reported a benefit in physical performance, whereas none demonstrated a comparative increase in body composition-related muscle quantity [14,34,36,37,39,40]. Preclinical studies of polyphenols and mitochondria-modulating antioxidants broadened this evidence to mitochondrial, contractile, and tissue-level structural outcomes [41,51,52,55]. Nrf2- and glutathione-related strategies, by contrast, remain supported mainly by preclinical mechanistic evidence [43,60,61]. This classification therefore provides a practical framework for interpreting heterogeneous findings, but the categories should not be regarded as biologically exclusive because many antioxidants simultaneously influence redox, mitochondrial, inflammatory, and metabolic pathways.

Most studies did not directly measure muscle quality, such as intramuscular fat, muscle density, specific force, fiber quality or imaging-based parameters. The reported effects more commonly involved functional outcomes, including muscle strength and physical performance measures, mitochondrial endpoints, redox-related markers or histological features [14,35,39,41,50,51,52]. Future studies should combine muscle imaging, specific-force assessment, histology, mitochondrial evaluation, and separate measures of muscle strength and physical performance to clarify whether functional improvements occur independently of changes in muscle quantity or quality.

The observed differences between functional and structural outcomes should be interpreted cautiously. Functional outcomes may be more responsive than structural measures over short intervention periods, and several clinical trials included exercise or nutritional co-interventions that could preferentially affect muscle strength or physical performance. Although redox- or mitochondrial-related changes may precede detectable changes in muscle quantity, the available evidence does not establish such a temporal sequence. The apparent functional–structural divergence should therefore be considered hypothesis-generating rather than a demonstrated biological effect of antioxidant intervention. Redox biology should be interpreted in terms of homeostasis rather than simple ROS suppression. ROS are not exclusively damaging molecules but also serve as physiological signals involved in muscle contraction and exercise-induced adaptation [16]. Accordingly, the objective of antioxidant intervention should be restoration of an appropriate redox balance rather than maximal elimination of ROS. This framework may help explain why effects differ according to baseline nutritional or redox status, exercise exposure, dose, treatment duration, and disease context. It also highlights the possibility that high-dose antioxidant supplementation may attenuate beneficial exercise-induced redox signaling and selected skeletal-muscle adaptations [16,69]. Future studies should therefore define the appropriate compound, dose, timing, target population, and co-intervention context rather than assuming that stronger ROS suppression necessarily produces better outcomes.

This review has several limitations. The clinical evidence comprised only eight heterogeneous trials, several of which included exercise or multinutrient co-interventions and follow-up periods of no more than 6 months. These factors limited attribution of the observed effects to antioxidant compounds and may have reduced the ability to detect changes in muscle quantity. Women predominated in several studies, but sex-by-treatment interactions were rarely examined. Meta-analysis was not appropriate because of the limited number of comparable studies and the substantial heterogeneity in interventions, populations, and outcome definitions. Preclinical studies used models with differing relationships to physiological aging; chemically induced aging-like and experimentally induced atrophy or disuse models are useful for interrogating selected mechanisms but do not fully recapitulate spontaneous age-related sarcopenia [70]. In addition, few studies evaluated redox target engagement in human skeletal muscle. The review was not prospectively registered, was restricted to English-language full-text publications, and excluded reports that could not be retrieved, which may have introduced selection bias and reduced the completeness of the evidence base.

Given the limited clinical evidence, frequent use of combined interventions, and lack of target-engagement data in human skeletal muscle, antioxidants cannot currently be recommended as routine standalone treatment for sarcopenia or broader aging-related muscle decline. A more appropriate interpretation is that specific antioxidant strategies may improve selected outcomes in particular nutritional, exercise, or disease contexts, rather than that antioxidants exert a uniform therapeutic effect across populations.

An additional source of clinical variability is the heterogeneity of sarcopenia itself. Individual patients may differ in the relative contribution of neuromuscular denervation, mitochondrial dysfunction, chronic inflammaging, nutritional deficiency, and anabolic resistance; consequently, a single broad-spectrum antioxidant strategy is unlikely to produce uniform benefit [5]. Future clinical trials should stratify participants using clinically and mechanistically relevant features and align antioxidant selection with the dominant phenotype. Such phenotype-guided treatment may provide a more realistic framework for translating targeted preclinical findings into personalized clinical management.

Future evidence syntheses should avoid indiscriminate pooling of mechanistically distinct antioxidant interventions. In parallel, future trials should be organized more explicitly according to antioxidant category and mechanistic hypothesis. Studies of vitamins should distinguish deficient from non-deficient participants and clarify the contribution of exercise or protein-based nutritional support [35,38,57]. Polyphenol studies should measure physical performance, muscle composition, mitochondrial function and proteostasis in parallel, in order to distinguish functional improvement, metabolic remodeling and measurable structural preservation [14,29,30,32,33,39,40,41,51,55]. More broadly, future mechanistic studies should distinguish pathway-marker changes from direct assessment of skeletal-muscle protein turnover, particularly when translating proteostasis findings between animal models and humans [71]. Mitochondria-targeted antioxidants need to be tested in human sarcopenia trials and should include outcomes such as fatigue resistance, specific force or mitochondrial function [27,52,56]. Nrf2-related compounds need to move from cellular causal validation to animal and human target-engagement studies [60,61]. Glutathione-supporting strategies should incorporate muscle strength, walking ability and long-term muscle-mass outcomes [35,43]. Only when intervention components are clearly defined, follow-up is sufficiently long, and mechanistic indicators are measured alongside clinical outcomes can the translational potential of antioxidant strategies be properly evaluated.

## 5. Conclusions

In summary, antioxidant interventions across clinically defined sarcopenia and broader models of aging-related muscle decline did not produce uniform structural or functional responses. Clinical evidence suggested that the apparent functional–structural divergence was driven mainly by selected improvements in muscle strength or power, with more limited support for physical performance and body composition-related muscle quantity. Given the small and heterogeneous clinical evidence base, this pattern remains hypothesis-generating and requires confirmation in adequately powered trials with longer follow-up and concurrent assessment of strength, physical performance, and muscle quantity. Future trials should therefore stratify participants by dominant sarcopenic phenotype and align antioxidant selection with the relevant neuromuscular, mitochondrial, inflammatory, or nutritional context. Preclinical studies provided additional structural and mechanistic insights; however, most pathways were supported by associative changes in molecular markers, with direct experimental validation limited to a small number of cellular studies. Findings from accelerated-aging or deficiency, chemically induced aging-like, experimentally induced atrophy or disuse, and cellular models should therefore be regarded primarily as mechanistic evidence and should not be extrapolated directly to clinical sarcopenia.

## Figures and Tables

**Table 1:** **Basic Characteristics of the Included Clinical Studies.**

Study	Participants	Age	Intervention(s) and Comparator	Antioxidant Class	Duration	Outcome Measures	Muscle-Related Results	Mechanisms and Biomarkers	Key Findings
**Bo, 2019** [38]	60	Intervention: 73.23 ± 6.52 Placebo: 74.83 ± 5.94	Whey protein + Vitamin D + Vitamin E (twice/day) Iso-caloric placebo Dose: VD 1404 IU/day; VE 218 mg/day	Vitamins/antioxidant micronutrients	6 months	Body comp: ASM, RSMI (BIA) Muscle strength: handgrip (dynamometer) Physical performance: 6-m gait speed, TUG, chair stand (5 rises) PROs: QoL (SF-36: MCS, PCS), nutritional status (MNA-SF) Compliance & diet: diary, 3-d diet record Blood biomarkers: CRP, albumin, lipids, TNF-α, IGF-1, IL-2, IL-6, 25-OH-D_3_, vit E	**Muscle mass** BIA: appendicular skeletal muscle mass showed no significant difference between groups (*p* = 0.050) RSMI (relative skeletal muscle index) was significantly higher in the intervention group compared with placebo (*p* = 0.040) **Muscle strength:** Handgrip strength was significantly higher in the intervention group compared with placebo (*p* = 0.009) **Physical performance:** 6-m gait speed showed no significant difference between groups (*p* = 0.402) Chair stand test time showed no significant difference between groups (*p* = 0.176) Timed up and go test time showed no significant difference between groups (*p* = 0.383) **Quality of life** SF-36 mental component summary score was significantly higher in the intervention group compared with placebo (*p* = 0.004) SF-36 physical component summary score was significantly higher in the intervention group compared with placebo (*p* < 0.001)	**Anabolic and inflammatory markers** Serum IGF-1 was significantly higher in the intervention group compared with placebo (*p* = 0.023) Serum IL-2 was significantly lower in the intervention group compared with placebo (*p* = 0.038) No significant differences were observed for IL-6 or TNF-α (both *p* > 0.05) **Other biomarkers** Serum 25-hydroxyvitamin D_3_ was significantly higher in the intervention group compared with placebo (*p* < 0.001) Serum vitamin E was significantly higher in the intervention group compared with placebo (*p* = 0.001) Body weight, BMI, and fat mass showed no significant differences between groups (all *p* > 0.05)	Combined whey protein, vitamin D, and vitamin E supplementation significantly improved RSMI and handgrip strength compared with placebo Appendicular skeletal muscle mass and physical function tests (gait speed, chair stand, TUG) showed no significant differences between groups Quality of life (SF-36) was significantly improved in the intervention group Serum IGF-1 was significantly increased and IL-2 significantly decreased with supplementation
**Liu, 2025 [5]**	60	Intervention: 66.07 ± 4.05 Placebo: 65.65 ± 4.60	Vitamin C + Vitamin E + Resistance Training structured RT Dose: VC 1000 mg/day + VE 335 mg/day	Vitamins / antioxidant micronutrients	12 weeks	Muscle mass: total, arm, trunk, leg lean mass, SMI (DXA) Muscle strength: handgrip, knee extension Physical performance: 5-repetition chair stand, TUG, 6-m gait speed Oxidative stress & inflammation: GSH, GSSG, MDA, PCO, IL-6, TNF-α (ELISA) Antioxidant vitamins: serum vitamin C, vit E (HPLC) Anthropometry: height, weight, BMI Compliance & control: training diary, 3-d food record, IPAQ	**Muscle mass** DXA: arm lean mass increased in both groups, with a significantly greater increase in the antioxidant supplementation group compared with placebo (*p* = 0.003, Cohen’s d = 0.74) DXA: leg lean mass increased in both groups, with no significant difference between groups (*p* = 0.936) DXA: trunk lean mass showed no significant change in either group, with no significant difference between groups (*p* = 0.528) DXA: total lean mass increased in both groups, with no significant difference between groups (*p* = 0.375) Skeletal muscle index increased in both groups, with a significantly greater increase in the antioxidant supplementation group compared with placebo (*p* = 0.004, Cohen’s d = 0.71) **Muscle strength:** Handgrip strength increased in both groups, with a significantly greater increase in the antioxidant supplementation group compared with placebo (*p* = 0.047, Cohen’s d = 0.51) Knee extension strength increased in both groups, with a significantly greater increase in the antioxidant supplementation group compared with placebo (*p* < 0.001, Cohen’s d = 0.89) **Physical performance:** Five-repetition chair stand test time decreased in both groups, with no significant difference between groups (*p* = 0.100) Timed up and go test time decreased in both groups, with no significant difference between groups (*p* = 0.946) Six-meter gait speed decreased in the antioxidant supplementation group but not in the placebo group, with no significant difference between groups (*p* = 0.749)	Serum vitamin C levels increased in the antioxidant supplementation group but decreased in the placebo group, with a significantly higher level in the antioxidant supplementation group compared with placebo (*p* < 0.001, Cohen’s d = 1.70) Serum vitamin E levels increased in the antioxidant supplementation group but decreased in the placebo group, with a significantly higher level in the antioxidant supplementation group compared with placebo (*p* < 0.001, Cohen’s d = 1.63) Serum GSH increased in the antioxidant supplementation group but decreased in the placebo group, with a significantly higher level in the antioxidant supplementation group compared with placebo (*p* < 0.001, Cohen’s d = 1.52) Serum GSSG increased in both groups, with a significantly lower level in the antioxidant supplementation group compared with placebo (*p* < 0.001, Cohen’s d = 0.96) GSH/GSSG ratio increased in the antioxidant supplementation group but decreased in the placebo group, with a significantly higher ratio in the antioxidant supplementation group compared with placebo (*p* < 0.001, Cohen’s d = 1.52) Serum MDA decreased in the antioxidant supplementation group but increased in the placebo group, with a significantly lower level in the antioxidant supplementation group compared with placebo (*p* < 0.001, Cohen’s d = 1.65) Serum PCO showed no significant difference between groups (*p* = 0.074) Inflammatory markers Serum IL-6 decreased in both groups, with a significantly greater decrease in the antioxidant supplementation group compared with placebo (*p* < 0.001, Cohen’s d = 1.16) Serum TNF-α decreased in both groups, with no significant difference between groups (*p* = 0.082)	Vitamins C and E supplementation combined with 12 weeks of resistance training significantly improved arm lean mass, skeletal muscle index, handgrip strength, and knee extension strength compared with resistance training with placebo The intervention significantly improved oxidative stress markers (increased GSH and GSH/GSSG ratio, decreased MDA) and reduced IL-6 levels Leg lean mass, trunk lean mass, total lean mass, and physical performance tests showed no significant differences between groups All participants had inadequate baseline vitamin C levels, and 57% had inadequate vitamin E levels, suggesting that antioxidant supplementation may be particularly beneficial in sarcopenic populations with poor antioxidant status The intervention was well tolerated with no adverse events reported
**Seo, 2021 [40]**	67	Intervention: 64.8 ± 3.7 Placebo: 63.5 ± 3.7	Tannase-treated green tea extract Microcrystalline cellulose placebo Dose: 600 mg/day	Polyphenols / plant-derived extracts	12 weeks	Muscle strength: isokinetic knee flexor/extensor peak torque & TQ/BW at 60°/s (Biodex), handgrip (Jamar) Muscle mass: arm, leg, trunk muscle mass (DXA) Physical performance: SPPB total score Blood biomarkers: follistatin, myostatin, IL-6, IL-8, IGF-1, cortisol (ELISA) Safety: vital signs, AEs, blood counts, liver & renal function Compliance: protocol compliance	**Muscle strength:** Isokinetic peak torque of the right leg flexor muscles was significantly higher in the treatment group compared with placebo (*p* = 0.013) Isokinetic peak torque/body weight ratio of the right leg flexor muscles was significantly higher in the treatment group compared with placebo (*p* = 0.019) Other isokinetic parameters (left leg flexor, bilateral extensors) showed no significant differences between groups (all *p* > 0.05) Right handgrip strength was significantly higher in the treatment group compared with placebo (*p* = 0.037) Left handgrip strength showed no significant difference between groups (*p* > 0.05) **Muscle mass** Arm muscle mass (left and right) showed no significant difference between groups (both *p* > 0.05) Trunk muscle mass showed no significant difference between groups (*p* > 0.05) Leg muscle mass (left and right) showed no significant difference between groups (both *p* > 0.05) Total body muscle mass showed no significant difference between groups (*p* > 0.05)	**Myostatin and related markers** Serum myostatin was significantly lower in the treatment group compared with placebo (*p* = 0.045) Serum follistatin showed no significant difference between groups (*p* > 0.05) Serum IGF-1 showed no significant difference between groups (*p* > 0.05) Serum cortisol showed no significant difference between groups (*p* > 0.05) **Inflammatory markers** Serum IL-6, IL-8, and hs-CRP showed no significant differences between groups (all *p* > 0.05) Antioxidant activity (in vitro) ABTS and DPPH radical-scavenging activities were significantly higher in tannase-treated extract compared with untreated extract (*p* < 0.001) FRAP and GSH levels showed no significant differences between treated and untreated extracts (*p* > 0.05)	Tannase-treated green tea extract, containing high levels of (−)-epicatechin and gallic acid, significantly increased antioxidant activity compared with untreated extract Twelve weeks of supplementation without concomitant exercise significantly improved right leg flexor strength and right handgrip strength compared with placebo Arm muscle mass was preserved in the treatment group, whereas it significantly decreased in the placebo group; however, no significant between-group differences were observed for any muscle mass parameters Serum myostatin levels were significantly reduced in the treatment group, suggesting a potential mechanism for the observed improvements in muscle strength No significant changes were observed in follistatin, IGF-1, cortisol, or inflammatory markers The intervention was safe and well tolerated, with no significant differences in adverse events or laboratory safety parameters between groups
**Cho, 2021 [36]**	54	Intervention: 60.4 ± 8.5 Placebo: 59.2 ± 8.8	Schisandra chinensis extract Placebo (corn starch) Dose: 1 g/day	Polyphenols / plant-derived extracts	12 weeks	Muscle strength/power: knee extension peak torque at 60°/s (Biodex) Muscle strength: handgrip (Jamar) Body comp: total fat mass, nonfat mass, body fat %, android & gynoid fat, ASM/height^2^ (DXA) Blood biomarkers: CRP, TNF-α, IL-6, MDA, creatinine, pyruvate, lactate, CK, glucose, insulin, lipid profile, LFTs, electrolytes QoL: EQ-5D Safety: BP, pulse, CBC, LFTs, renal function, CK, glucose, lipids Compliance: protocol adherence, supplement log	**Muscle strength:** Isokinetic peak torque of right knee extension at 60°/s was significantly higher in the Schisandra chinensis extract group compared with placebo (*p* = 0.003) Isokinetic peak torque of left knee extension at 60°/s was significantly higher in the Schisandra chinensis extract group compared with placebo (*p* = 0.041) Handgrip strength (intention-to-treat analysis) showed no significant difference between groups (*p* > 0.05) In the per-protocol analysis, right handgrip strength was significantly higher in the Schisandra chinensis extract group compared with placebo (*p* = 0.048) **Muscle mass and body composition** Appendicular skeletal muscle mass index showed no significant difference between groups (*p* > 0.05) Total fat mass, body fat percentage, and android/gynoid fat mass showed no significant differences between groups (all *p* > 0.05)	**Inflammatory and oxidative stress markers** Serum TNF-α, IL-6, and C-reactive protein showed no significant differences between groups (all *p* > 0.05) Serum malondialdehyde showed no significant difference between groups (*p* > 0.05) Metabolic and muscle-related markers Serum creatine kinase, creatinine, pyruvate, and lactate showed no significant differences between groups (all *p* > 0.05) **Other biomarkers** Fasting blood glucose was significantly lower in the Schisandra chinensis extract group compared with placebo (*p* = 0.015) Fasting insulin and lipid profile (total cholesterol, LDL-C, HDL-C, triglycerides) showed no significant differences between groups (all *p* > 0.05) Liver enzymes (AST, ALT, GGT) and blood pressure showed no significant differences between groups (all *p* > 0.05) **Quality of life** Euro-QoL-5D score showed no significant difference between groups (*p* > 0.05)	Schisandra chinensis extract supplementation for 12 weeks significantly improved isokinetic knee extension strength (both right and left) compared with placebo in older adults with relatively low muscle mass who performed low-intensity walking exercise Handgrip strength improvement was significant only in the per-protocol analysis for the right hand No significant effects were observed on muscle mass, body composition, inflammatory markers, oxidative stress markers, or quality of life Fasting blood glucose was significantly lower in the Schisandra chinensis extract group, but insulin levels did not differ The intervention was safe and well tolerated, with no significant adverse events or changes in safety laboratory parameters The lack of effect on muscle mass may be due to the relatively low dose (1 g/d) compared with animal-based equivalent doses; higher doses may be needed to increase muscle mass
**Kwon, 2021 [34]**	20	Intervention: 74.63 ± 9.30 Placebo: 73.38 ± 10.66	Marine oligomeric polyphenols placebo Dose: 14 mg/day	Polyphenols / plant-derived extracts	4 weeks	Body comp: SMM, BMI, %BF, ALM, %FFMI, calf circumference (BIA, DEXA, tape) Bone density: T-score (DEXA) Muscle strength: grip strength (dynamometer) Physical performance: 5-rep chair rise time, 2.4-m up & go time, one-leg stand time Disease status: sarcopenia diagnosis (ALM cut-off) Compliance: product compliance (phone monitoring)	**Muscle mass** BIA: skeletal muscle mass increased significantly over time in both groups combined (*p* = 0.039), but no significant between-group difference or interaction was observed DEXA: lean body mass increased significantly over time in both groups combined (*p* = 0.018), with no between-group difference or interaction DEXA: % fat-free mass index increased significantly over time in both groups combined (*p* = 0.026), with no between-group difference or interaction BIA: intracellular fluid increased significantly over time in both groups combined (*p* = 0.031), with no between-group difference or interaction BIA: body weight, BMI, body fat percentage, total body water, and extracellular fluid showed no significant changes over time or between groups (all *p* > 0.05) Calf circumference showed no significant changes over time or between groups (both *p* > 0.05) **Muscle strength:** Handgrip strength (left and right) showed no significant changes over time or between groups (all *p* > 0.05) **Physical performance:** Chair rise test showed no significant changes over time or between groups (*p* > 0.05) 2.4 m up and go test time decreased significantly over time in both groups combined (*p* = 0.001), indicating improved dynamic balance, with no between-group difference or interaction One-leg stand test time increased significantly over time (*p* = 0.010), and there was a significant time × group interaction (*p* = 0.049), with the marine oligomeric polyphenol group showing greater improvement than the placebo group; no significant main effect of group was observed **Bone density** DEXA: bone mineral density (t-score) increased significantly over time in both groups combined (*p* = 0.020), with no between-group difference or interaction	*n*/A	Marine oligomeric polyphenol intake for 4 weeks resulted in significant time-dependent improvements in skeletal muscle mass, lean body mass, fat-free mass index, intracellular fluid, bone mineral density, and 2.4 m up and go test performance in older adults with sarcopenia, but no significant between-group differences were observed for any variable A significant time × group interaction was observed for the one-leg stand test, suggesting that marine oligomeric polyphenol intake may improve static balance more effectively than placebo No significant effects were observed on body weight, BMI, body fat percentage, calf circumference, grip strength, or chair rise test The lack of consistent between-group differences may be due to the small sample size (pilot study) and short intervention duration (4 weeks) Future longer-term studies with larger sample sizes and combined exercise intervention are needed to confirm the efficacy of marine oligomeric polyphenols for sarcopenia
**Alway, 2017 [14]**	30	Intervention: 68.1 ± 1.1 Placebo: 67.9 ± 1.1	Resveratrol + aerobic + RT aerobic+ RT Dose: 500 mg/day	Polyphenols / plant-derived extracts	12 weeks	Body comp: body weight, BMI, %BF, waist, hip, WHR Blood indices: fasting glucose, insulin, HbA1c, lipid profile Exercise capacity: VO_2_max, VCO_2_, RER, VE, VT, VE/VCO_2_, VE/VO_2_ (graded exercise test) Muscle strength/power: peak torque, total work (1 & 7 reps), work fatigue %, avg power, avg peak torque (7 reps), fatigue index (Biodex at 60°/s & 240°/s) Muscle histology & molecular: fiber type (I, IIA, IIX), CSA, min Feret diameter, mitochondrial volume fraction (EM), mtDNA copy number, mitochondrial metabolism gene expression (PCR array), satellite cell count (Pax7^+^), myonuclei count, nuclear DNA oxidative damage (8-OHdG)	**Muscle mass and body composition** Body weight, body fat percentage, lean body mass, and BMI showed no significant differences between the resveratrol group and the placebo group after 12 weeks of exercise (all *p* > 0.05) **Muscle strength:** Knee extension peak torque at 60°/s increased significantly in the resveratrol group compared with the placebo group after training (*p* < 0.05 for group × time interaction) Knee extension average peak torque at 60°/s increased significantly in the resveratrol group compared with the placebo group (*p* < 0.05) Knee extension average power at 60°/s increased significantly in the resveratrol group compared with the placebo group (*p* = 0.004 for group × time interaction) Other isokinetic parameters (total work, work fatigue, fatigue index) showed no significant differences between groups (all *p* > 0.05) **Muscle fiber morphology** Type I muscle fiber cross-sectional area increased significantly in the resveratrol group compared with the placebo group after training (*p* < 0.05) Type IIA muscle fiber cross-sectional area increased significantly in the resveratrol group compared with the placebo group (*p* < 0.05) Fiber type distribution did not change significantly in either group (*p* > 0.05) **Myonuclei and satellite cells** Total myonuclei per fiber increased significantly in the resveratrol group compared with the placebo group after training (*p* < 0.05) Pax7-positive satellite cell content per fiber increased significantly in the resveratrol group compared with the placebo group (*p* < 0.05)	**Mitochondrial density and function** Mitochondrial volume density increased significantly from pre- to post-training in the resveratrol group (*p* < 0.05), but not in the placebo group (*p* = 0.093); no significant between-group difference was detected Maximal oxygen uptake (absolute and relative) increased significantly in the resveratrol group compared with the placebo group (*p* < 0.05 for post-hoc comparisons; group × time interaction *p* = 0.09) **Gene expression** (mitochondrial-related, from pre- to post-training) Compared with placebo, resveratrol treatment resulted in greater than 2-fold increases in several genes involved in apoptosis (BAK1, BCL2, BCL2L1, BID, CDKN2A, PMAIP1, SFN, DNM1L, UXT), metabolism (CPT1B, COX18, UCP2, UCP3, HPRT1), mitochondrial morphology (MFN1, MFN2, MSTO1), and carrier/import proteins (multiple SLC25, TIMM, TOMM family members) Compared with placebo, resveratrol treatment resulted in greater than 2-fold decreases in genes involved in apoptosis/autophagy (SH3GLB1, RHOT1, RHOT2), metabolism (COX10, CPT2, GRPEL1, MIPEP), and carrier/import proteins (SLC25A4, SLC25A13, SLC25A14, SLC25A15, SLC25A19, SLC25A20, SLC25A21, SLC25A30, MTX2, TIMM23) **Oxidative stress** Nuclear oxidative DNA damage (8-OHdG-positive nuclei inside the basal lamina) decreased after exercise in both groups, with no significant difference between resveratrol and placebo groups (*p* > 0.05)	Twelve weeks of resveratrol supplementation (500 mg/d) combined with aerobic and resistance exercise significantly improved knee extensor peak torque, average peak torque, and average power in older men and women, whereas exercise alone did not Resveratrol plus exercise increased type I and type IIA muscle fiber cross-sectional area, total myonuclei, and Pax7-positive satellite cell content compared with exercise alone Mitochondrial volume density and maximal oxygen uptake were improved by resveratrol plus exercise, but not by exercise alone Resveratrol altered the expression of numerous mitochondrial-related genes (apoptosis, metabolism, morphology, import proteins) in a complex manner, with both up- and down-regulation observed No additive effects of resveratrol were observed on body composition, blood lipid profiles, insulin, or oxidative DNA damage beyond those of exercise alone The findings suggest that resveratrol may enhance exercise-induced cellular and functional adaptations in skeletal muscle of older adults, potentially offering a strategy to counteract sarcopenia
**Boutry-Regard, 2020 [37]**	22	Intervention: 76 ± 2 placebo: 78 ± 2	Whey protein isolate + Bioactives (Omega-3 fatty acids, rutin, curcumin) +EMS Carbohydrate (maltodextrin) + placebo capsules+EMS Dose: Whey protein 20 g/day Omega-3 fatty acids 1.5 g/day Rutin 500 mg/day Curcumin 500 mg/day	Polyphenols / plant-derived extracts	12 weeks	Muscle thickness: rectus femoris thickness, gastrocnemius thickness (ultrasonography) Muscle strength/power: knee extension MVC force (customized dynamometer) Physical performance: 6-m gait speed Nutritional status: MNA total score & risk category Body comp: fat mass, lean mass (BIA) Blood biomarkers: CRP, transthyretin, fibrinogen, orosomucoid, CPK, HDL, LDL, albumin, total protein, TG, RBC, WBC, platelet count, PT, PTT Safety: AEs, resting HR, BP Compliance: capsule count	**Muscle mass** Ultrasound: calf and thigh muscle thickness increased significantly over time in all groups (time effect *p* = 0.008 and *p* = 0.018, respectively), but no significant differences were observed between the three nutritional intervention groups (CHO, WPI, WPI+BIO) (all *p* > 0.05 for group and interaction) Bioelectrical impedance analysis: total lean mass (percentage and kg) showed no significant changes over time or between groups (all *p* > 0.05) **Muscle strength:** Knee extension maximal voluntary contraction force increased significantly from baseline to 12 weeks only in the WPI+BIO group (whey protein + omega-3, curcumin, rutin) (*p* = 0.042 for time effect) The change in knee extension strength was significantly greater in the WPI+BIO group compared with the CHO group (*p* = 0.025), corresponding to a 13% improvement in the WPI+BIO group versus 5% in CHO and 6% in WPI alone No significant differences were observed between the WPI alone group and the CHO group, or between WPI+BIO and WPI alone **Physical performance:** Six-meter gait speed improved significantly only in the WPI+BIO group (from 1.10±0.14 to 1.19±0.13 m/s, *p* = 0.032 for time effect), representing an 8% increase No significant between-group differences were detected for gait speed (ANCOVA, *p* > 0.05)	The study did not measure direct mechanistic biomarkers (e.g., inflammatory cytokines, oxidative stress markers) that could explain the observed functional improvements Blood markers of inflammation (CRP, transthyretin, fibrinogen, orosomucoid), muscle damage (creatine phosphokinase), lipid profile, and coagulation parameters showed no significant differences between groups (all *p* > 0.05) Nutritional status assessed by Mini-Nutritional Assessment improved numerically in the WPI+BIO group (50% of participants improved) compared with CHO (25%) and WPI (13%), but the difference was not statistically significant	Electrical muscle stimulation alone (all groups) significantly increased thigh and calf muscle thickness by 3–5% after 12 weeks, regardless of nutritional supplementation Addition of whey protein alone did not provide additional benefits over carbohydrate control on muscle strength, gait speed, or lean mass Whey protein combined with omega-3 fatty acids, curcumin, and rutin (WPI+BIO) significantly improved knee extension strength compared with carbohydrate control (+13% vs. +5%, *p* = 0.025) and produced the largest improvement in gait speed (+8%, time effect *p* = 0.032) No significant effects on body composition (lean mass, fat mass) were observed in any group The functional improvements with WPI+BIO occurred without changes in muscle size, suggesting enhanced muscle quality rather than hypertrophy The combination of polyphenols and omega-3 fatty acids may improve muscle function by reducing oxidative stress, inflammation, or anabolic resistance, but further mechanistic studies are needed This multimodal approach (EMS + specific nutritional blend) could be a promising strategy for sarcopenia prevention, but larger studies are required to confirm efficacy
**Munguía, 2019 [39] **	74	75.9 ± 5.7	Flavonoid-rich natural cocoa No-flavonoid cocoa Dose: 179 mg/day	Polyphenols / plant-derived extracts	8 weeks	Anthropometry: height, weight, waist, BMI Clinical chemistry: glycemia, TC, LDL, HDL, TG, TG/HDL index Oxidative stress & inflammation: MDA, protein carbonyls, IL-6, TNF-α (ELISA) Muscle mass & strength: SMI (estimated), handgrip (dynamometer) Physical performance & mobility: 6-min walk distance, 2-min step test, 30-s chair stand, TUG QoL: EQ-5D Frailty status: Fried phenotype (weight loss, exhaustion, weakness, slowness, low activity) Disease status: sarcopenia diagnosis (SMI cut-off) Compliance: empty sachet return	**Muscle mass (initial study, 55–70 years, 12 weeks)** Skeletal muscle index (SMI) increased significantly only in the flavonoid-rich cocoa group compared with baseline (*p* = 0.03), with no significant changes in the placebo or no-flavonoid groups **Muscle strength:** **Initial study:** Handgrip strength showed no significant changes in any group **Follow-up study (65–90 years, 8 weeks, flavonoid-rich cocoa vs. no-flavonoid cocoa):** Handgrip strength increased significantly in the flavonoid group compared with the no-flavonoid group (*p* < 0.05) **Physical performance:** **Initial study:** Up and Go test time decreased significantly only in the flavonoid-rich cocoa group (*p* = 0.04), indicating improved mobility **Follow-up study:** Six-minute walk distance increased significantly in the flavonoid group compared with the no-flavonoid group (*p* < 0.05) Two-minute step test count increased significantly in the flavonoid group compared with the no-flavonoid group (*p* < 0.05) Thirty-second sit-up test repetitions increased significantly in the flavonoid group compared with the no-flavonoid group (*p* < 0.05) Up and Go test time decreased significantly in the flavonoid group compared with the no-flavonoid group (*p* < 0.05) The proportion of pre-frail subjects decreased by 29.4% in the flavonoid group, with no change in the no-flavonoid group **Quality of life** Visual analog scale quality of life improved significantly only in the flavonoid-rich cocoa group (initial study) In the follow-up study, the flavonoid group showed a 66% reduction in reported problems with mobility and pain/discomfort on the EQ-5D scale	**Oxidative stress markers (initial study)** Serum malondialdehyde (lipid peroxidation) decreased significantly only in the flavonoid-rich cocoa group Serum protein carbonyls decreased significantly only in the flavonoid-rich cocoa group **Oxidative stress and inflammatory markers (follow-up study)** Serum malondialdehyde and protein carbonyls were significantly lower in the flavonoid group compared with the no-flavonoid group Serum interleukin-6 was significantly lower in the flavonoid group compared with the no-flavonoid group (*p* < 0.05) Serum tumor necrosis factor-α showed a trend toward reduction but did not reach statistical significance (*p* = 0.06) **Cardiometabolic markers (initial study)** Glycemia decreased significantly only in the flavonoid-rich cocoa group (*p* = 0.04) LDL cholesterol decreased significantly in all groups, with the largest reduction in the flavonoid group (-11.5 mg/dL, *p* = 0.01) HDL cholesterol increased significantly only in the flavonoid group (*p* = 0.003) Triglycerides decreased significantly only in the flavonoid group (*p* = 0.01) Triglyceride/HDL ratio decreased significantly only in the flavonoid group	Twelve weeks of flavonoid-rich cocoa supplementation (179 mg flavonoids/day) in middle-aged subjects (55–70 years) significantly improved skeletal muscle index, Up and Go test performance, glycemic control, lipid profile (↓LDL, ↑HDL, ↓triglycerides), and oxidative stress markers (↓malondialdehyde, ↓protein carbonyls) compared with placebo or no-flavonoid cocoa Eight weeks of flavonoid-rich cocoa supplementation in older subjects (65–90 years) significantly improved multiple physical performance measures (6-minute walk, step test, sit-up test, Up and Go test, handgrip strength), reduced pre-frailty prevalence by 29.4%, and improved quality of life compared with no-flavonoid cocoa The improvements in physical function were accompanied by reductions in oxidative stress (malondialdehyde, protein carbonyls) and inflammation (interleukin-6), suggesting that flavonoid-induced mitigation of oxidative stress and inflammation may underlie the functional benefits No significant changes in handgrip strength were observed in the initial study, but improvements were detected in the older cohort The sum of these effects suggests that regular flavonoid consumption may help mitigate frailty development in the elderly population

Clinical context: Four trials used explicit sarcopenia-related diagnostic or muscle-mass criteria, whereas the remaining trials enrolled older adults with broader age-associated muscle impairment without requiring a formal diagnosis of sarcopenia. **Abbreviations:** ABTS, 2,2′-azinobis(3-ethylbenzothiazoline-6-sulfonic acid); AE, adverse event; ASM, appendicular skeletal muscle mass; BIO, bioactives; BMI, body mass index; CBC, complete blood count; CHO, carbohydrate; CRP, C-reactive protein; DPPH, 2,2-diphenyl-1-picrylhydrazyl; DPPH, 2,2-diphenyl-1-picrylhydrazyl; DXA, dual-energy X-ray absorptiometry; ELISA, enzyme-linked immunosorbent assay; FRAP, ferric reducing antioxidant power; GSH, reduced glutathione; GSSG, oxidized glutathione; HPLC, high-performance liquid chromatography; IGF-1, insulin-like growth factor-1; IL-6, interleukin-6; IPAQ, International Physical Activity Questionnaire; LFT, liver function test; MDA, malondialdehyde; NS, not significant; *n*/A, not applicable or not assessed; PCO, protein carbonyls; QoL, quality of life; RT, resistance training; SMI, skeletal muscle index; SPPB, Short Physical Performance Battery; TNF-α, tumor necrosis factor-alpha; TUG, Timed Up and Go; WPI, whey protein isolate. **Note:** ↑, increase; ↓, decrease.

**Table 2:** **Basic Characteristics of the Included Preclinical Animal Studies.**

Study	Species and Sex	Age/Model	Intervention(s)	Target Muscle(s)	Muscle Mass or Morphology	Muscle Function	Protein Metabolism and Signaling	Mitochondrial and Oxidative-Stress Outcomes	Key Findings
**Marzani 2008 [45]**	Wistar rat (male)	Adult: 8 mo Old: 20 mo Natural aging	**Type:** Rutin, Vitamin E, Vitamin A, Zinc, Selenium (combined) Vehicle: Incorporated into pelleted diet (AIN-93M base with modifications) Route: Oral dietary supplementation Dose (g/kg diet): Rutin: 5.0 others based on AIN-93M with modifications – exact modified doses *n*/A in g/kg diet **Frequency:** Continuous for 7 weeks Vitamin E, A, zinc, selenium were increased relative to AIN-93M, but the final g/kg diet values were not provided by authors.	Epitrochlearis muscle, Gastrocnemius, Tibialis anterior, Extensor digitorum longus (EDL), Soleus	**Gastrocnemius, tibialis anterior, EDL, soleus weights** No significant difference between old supplemented (OAox+) vs. old control (OAox–) for any muscle (*p* > 0.05) **Myofiber CSA** N/A quantitatively; authors state no significant effect of supplementation on muscle mass or fiber size	**N/A**	**Leucine-stimulated protein synthesis** **Old control vs. adult control:** ↓ response (*p* < 0.05) Old supplemented vs. old control: ↑ response to adult level (*p* < 0.05) Leucine-induced inhibition of degradation Old control vs. adult control: tendency to be lower Old supplemented vs. old control: improved (*p* value not given, described as “tended to improve”) S6K oxidation (carbonyl content) No significant difference between any groups (age or diet effect)	**Heart TBARS (lipid peroxidation)** Old supplemented vs. old control: ↓ (*p* < 0.0001) **Plasma inflammatory markers** α2-macroglobulin: old supplemented vs. old control ↓ (*p* < 0.05) Fibrinogen & albumin: no diet effect	Antioxidant supplementation (7 weeks) restores the defective leucine-stimulated muscle protein synthesis in old rats to the level of adult rats, without increasing leucine availability. The improvement is not explained by reduced oxidative damage to S6K protein (carbonyl content unchanged). The effect may be mediated by a reduction in low-grade inflammation (↓ plasma α2-macroglobulin) and/or oxidative stress (↓ heart TBARS). The supplementation period was likely too short to observe a significant improvement in muscle mass or creatinine excretion. Longer-term studies are needed to determine if this nutritional strategy can slow sarcopenia.
**Zainul Azlan, 2020 [49]**	Sprague-Dawley rat (male)	Young: 3 mo Old: 21 mo Natural aging	**Type:** Chlorella vulgaris Beijerinck strain 072 (algal supplement) Vehicle: Harvested algae centrifuged and diluted in distilled water Route: Oral gavage Dose: 150 or 300 mg/kg body weight Frequency: Once daily (morning) for 90 consecutive days	Gastrocnemius	Gastrocnemius relative weight No significant difference between C. vulgaris-treated and control groups in either young or old rats (*p* > 0.05)	**Muscle strength:** **Grip strength (front & hind paws)** Young: 150 or 300 mg/kg ↑ vs. control on days 30,60,90 (*p* < 0.05) Old: 300 mg/kg ↑ hind paw grip strength on day 90 vs. control (*p* < 0.05); front paw not significantly different from control **Physical performance:** **Open field test** Young (300 mg/kg): total path length ↑ vs. control on days 30,60,90 (*p* < 0.05); % time moving ↑ vs. control on days 30,60,90 (*p* < 0.05) Old (300 mg/kg): total path length ↑ on days 60,90 vs. day 0 control old rats (*p* < 0.05); % time moving no significant difference vs. control old rats	**N/A**	**Urinary isoprostane F_2t_** **Young:** 150 or 300 mg/kg ↓ vs. control on day 90 (*p* < 0.05) Old: 150 or 300 mg/kg ↓ vs. control on day 90 (*p* < 0.05) Plasma creatine kinase-MM (CK-MM) Young: 150 or 300 mg/kg ↓ vs. control on day 90 (*p* < 0.05) Old: 150 or 300 mg/kg ↓ vs. control on day 90 (*p* < 0.05) Skeletal muscle MDA + HAE (gastrocnemius) Young: 150 or 300 mg/kg ↓ vs. control (*p* < 0.05) Old: 150 or 300 mg/kg ↓ vs. control (*p* < 0.05)	C. vulgaris supplementation for 3 months improves muscle strength (grip strength) and function (open field total path) in young and old rats. It reduces oxidative stress markers (urinary isoprostane, plasma CK-MM, muscle MDA+HAE) in both age groups. Body composition improves: ↑ bone mineral content (BMC), bone mineral density (BMD), lean mass; ↓ fat mass (especially at 300 mg/kg). No significant effect on gastrocnemius muscle mass or relative organ weights (heart, brain, muscle) – the benefit is primarily functional and oxidative stress-mediated, not hypertrophic.
**Gatineau, 2015 [46]**	Wistar rat (male)	16 mo (start) Natural aging + dietary challenge	**Type:** Rutin, Vitamin E, Vitamin A, Vitamin D, Selenium, Zinc (combined) Vehicle: Incorporated into dry powdered diets (starch-based or sucrose-based, AIN-93 base) Route: Oral dietary supplementation Dose: Rutin 5 g/kg diet; others: specified modifications to AIN-93 vitamin/mineral mixture – exact g/kg diet N/A Frequency: Daily (ad libitum then pair-fed) for 5 months	Gastrocnemius, tibialis anterior, extensor digitorum longus (EDL), soleus	**Absolute muscle weight** **Tibialis anterior:** sucrose-fed (Su) ↓ vs. starch-fed (St) (*p* = 0.02) Soleus: Su ↓ vs. St (*p* = 0.04) Gastrocnemius & EDL: no significant main effect of carbohydrate (*p* > 0.05) Relative muscle weight (%BW) All muscles: Su ↓ vs. St (*p* < 0.01 to *p* < 0.001) EDL: significant carbohydrate × R interaction (*p* = 0.03): R decreased weight in St, increased in Su Lean body mass Su lost more lean mass than St (−8.1% vs. −5.4%, *p* = 0.03) R supplementation had no significant effect on muscle or lean mass	**N/A**	Postprandial muscle protein synthesis (FSR) St: +22% increase with meal Su: only +7.3% increase (carbohydrate × nutritional state interaction, *p* = 0.04) R supplementation did not restore postprandial response Muscle protein concentration R supplementation ↑ (*p* < 0.01) regardless of diet Ribosomal capacity Su ↑ (*p* = 0.03) Carbohydrate × R interaction (*p* = 0.05): R decreased capacity in Su, no effect in St ASR & RE Only nutritional state effect (*p* < 0.01), no effect of sucrose or R	Muscle glutathione (tibialis anterior) Carbohydrate × R interaction: R ↑ glutathione in St (*p* = 0.03), no effect in Su Muscle SOD activity R supplementation ↓ (*p* = 0.01) irrespective of diet Plasma total antioxidant capacity R ↑ in both St and Su (*p* < 0.05) Plasma inflammation markers α2-macroglobulin: no significant final difference; sucrose tended to increase over last 2 mo (*p*-interaction = 0.02) Fibrinogen: no effect of carbohydrate or R IGF-I No effect of diet or R	High chronic sucrose intake (62% sucrose for 5 months) accelerates age-related loss of lean body mass and muscle mass in old male rats. This is mediated by a reduced postprandial stimulation of muscle protein synthesis, likely due to decreased insulin sensitivity (insulin sensitivity index halved in sucrose-fed rats), not via changes in IGF-I, inflammation, or oxidative stress. Antioxidant supplementation (R) slightly improved muscle protein concentration and some oxidative stress markers but did not prevent sucrose-induced acceleration of sarcopenia.
**Mohamad Ishak, 2024 [27]**	C57BL/6J mouse (male)	Young: 8 wk (~2 mo) Old: 83 wk (~19 mo) (Intervention 9 wk, old endpoint ~21 mo) Natural aging	**Type:** Pyrroloquinoline quinone (PQQ, BioPQQ) Vehicle: Mixed into AIN-93M diet pellets Route: Oral dietary supplementation Dose: ~19.9 mg/kg body weight/day (0.02% *w*/*w* in diet) Frequency: Daily for 9 weeks	Gastrocnemius (for gene expression), Soleus (for fiber CSA)	Soleus muscle fiber cross-sectional area (CSA) Fast myosin fibers: old + PQQ ↑ vs. old control (*p* = 0.020) Slow myosin fibers: old + PQQ vs. old control, not significant (*p* = 0.169, but trend toward increase) Total fibers: old + PQQ ↑ vs. old control (*p* = 0.005) Number of fibers No significant difference between old and old + PQQ for any fiber type	**Muscle strength:** **Hanging test (muscle strength)** Old control vs. young: ↓ latency time (*p* < 0.05) Old + PQQ: intermediate, not significantly different from either young or old control (*p* > 0.05) **Physical performance:** **Pole test (motor coordination)** Old + PQQ: ↓ sliding distance score vs. old control (*p* < 0.05, shown in Figure 3B)	Muscle inflammation (gastrocnemius mRNA) IL-6: old + PQQ ↓ vs. old control (*p* < 0.05) TNF-α: old + PQQ ↓ vs. old control (*p* < 0.05) IL-1β: no significant effect Satellite cell / regeneration genes Pax7, Myf5: no significant effect of PQQ Mitochondrial biogenesis genes PGC-1α, Tfam: no significant effect of PQQ	Not directly assessed in animal muscle (In vitro cell data show PQQ reduces ROS and improves mitochondrial function, but these were not measured in mouse muscle tissue.) Serum biomarkers (indirect) Creatine kinase (CK): ↓ trend in old + PQQ vs. old control (*p* = 0.641, NS) No other oxidative stress markers measured in vivo	Dietary PQQ (20 mg/kg/day for 9 weeks) hinders aging progression in aged male mice. PQQ attenuates muscle atrophy (increases fast-twitch fiber CSA). PQQ showed an intermediate response in the hanging test without a significant improvement versus old controls, whereas pole-test performance improved. PQQ reduces chronic inflammation in skeletal muscle (↓ IL-6, TNF-α). The anti-aging effect may be mediated by improved mitochondrial function and reduced oxidative stress (based on cell data), but in vivo mechanisms require further study. PQQ also improves integument conditions (skin frailty) and prevents rapid body fat loss and fluid accumulation, possibly via improving intestinal barrier function and metabolic homeostasis.
**Munguia, 2020 [55]**	C57BL/6 mouse (male)	64 wk (~15 mo) at start of treatment (aged + HFD-induced obesity) Natural aging + metabolic challenge	**Type:** (1) Dark chocolate beverage (DC) rich in flavonoids (epicatechin + procyanidins) OR (2) (−)-Epicatechin (EC) pure Vehicle: Water (gavage) Route: Intragastric (oral gavage) Dose: DC: 2 mg EC + 12.8 mg procyanidins / kg body weight; EC: 2 mg/kg body weight Frequency: Once daily for 5 weeks	Gastrocnemius	Gastrocnemius muscle weight No significant difference between vehicle, DC, or EC groups (*p* > 0.05) Fat mass (subcutaneous + visceral) DC and EC: ↓ vs. vehicle (*p* < 0.05, Body weight change DC: significant ↓ (−11.6 g vs. −2.5 g in vehicle, *p* < 0.05) EC: trend toward ↓ (−6.8 g, NS)	**Muscle strength:** **Weight-lifting test (forelimb strength)** EC: ↑ score change vs. vehicle (*p* < 0.05); DC: NS **Dynamometry (forelimb grip strength)** DC and EC: ↑ force vs. vehicle (*p* < 0.05) **Physical performance:** **Inverted screen test (strength/endurance)** DC and EC: ↑ time vs. vehicle (*p* < 0.05) **Hang wire test (coordination/strength)** DC: ↑ time vs. vehicle (*p* < 0.05); EC: NS	**Pro-growth/differentiation signaling** **Follistatin/myostatin ratio:** ↑ by DC and EC (*p* < 0.05) MEF2A protein expression: ↑ by DC and EC (*p* < 0.05) Ubiquitin-proteasome degradation pathway FOXO1A: ↓ by DC and EC (*p* < 0.05) MuRF1: ↓ by DC and EC (*p* < 0.05) MAFbx: NS (trend toward a decrease)	Serum oxidative stress markers Malondialdehyde (MDA): ↓ by DC and EC vs. vehicle (*p* < 0.05) Protein carbonylation: NS (trend toward a decrease)	Five weeks of DC or EC administration to aged (64 wk) mice with HFD-induced obesity improves physical performance (multiple tests) without changing muscle weight. The improvements are associated with increased follistatin/myostatin ratio and MEF2A (pro-growth), and decreased FOXO1A and MuRF1 (anti-atrophy). Serum lipid peroxidation (MDA) is reduced, suggesting reduced oxidative damage. DC and EC also reduce body weight and fat mass, and improve lipid profile (↓ LDL-c, triglycerides). Flavonoids from dark chocolate or pure (−)-epicatechin may protect against sarcopenic obesity.
**Annunziata, 2020 [48]**	Sprague-Dawley rat (male)	Young: 3 mo Old: 20 mo Natural aging	**Type:** Taurisolo® (grape pomace polyphenolic extract; contains catechin, epicatechin, procyanidins, resveratrol, etc.) Vehicle: Dissolved in water (100 mg/mL) Route: Oral gavage Dose: 100 mg/kg body weight Frequency: Once daily for 30 days	Gastrocnemius	**N/A**	**Physical performance:** **Rotarod test (motor coordination/endurance)** Old treated vs. old control: ↑ latency to fall (*p* < 0.05) Old treated at t30: not significantly different from young controls (*p* > 0.05)	Gene expression in gastrocnemius (qPCR) Antioxidant enzymes: CAT ↑, GPx ↑, Sirt1 ↑ (vs. old control, *p* < 0.05) Mn- SOD: ↑ trend (NS) Pro-inflammatory cytokine: IL-6 ↓ (vs. old control, *p* < 0.05) IL-10: NS	Total antioxidant capacity (FRAP) Old treated vs. old control: ↑ (*p* < 0.05); reached young level Antioxidant enzyme activities CAT: ↑ (*p* < 0.05) GPx: ↑ (*p* < 0.05) GRd: ↑ (*p* < 0.05) SOD: NS Oxidative damage markers Malondialdehyde (MDA, lipid peroxidation): old treated vs. old control ↓ (*p* < 0.05) Nitrotyrosine (N- Tyr, protein nitration): old treated vs. old control ↓ (*p* = 0.06, trend)	30-day oral administration of Taurisolo® (grape polyphenols) to aged rats improves motor coordination (Rotarod) to levels comparable to young rats. The treatment increases muscle total antioxidant capacity and the activities of key antioxidant enzymes (CAT, GPx, GRd), while reducing lipid peroxidation (MDA) and protein nitration (*n*-Tyr). It up-regulates the expression of antioxidant genes (CAT, GPx, Sirt1) and down-regulates the pro-inflammatory cytokine IL-6. These findings suggest that grape polyphenols can mitigate age-related oxidative stress in skeletal muscle and improve muscle performance, without direct measurement of muscle mass.
**Wang, 2020 [41]**	C57BL/6 mouse (male)	Young: 6–9 mo Old: 16–25 mo (treatment from 16 to 25 mo) Natural aging	**Type:** Apigenin Vehicle: Distilled water Route: Intragastric administration (gavage) Dose: 25, 50, or 100 mg/kg/day Frequency: Daily for 9 months	Tibialis anterior (TA), extensor digitorum longus (EDL), soleus (SOL), quadriceps, gastrocnemius	Muscle weight (absolute and normalized) Old control vs. young: ↓ TA, EDL, SOL weights and muscle/body weight ratios Apigenin (50 mg/kg): ↑ absolute and relative weights vs. old control (*p* < 0.05) Myofiber cross-sectional area (CSA) Old control vs. young: ↓ CSA Apigenin (50 mg/kg): ↑ CSA vs. old control (*p* < 0.05) Fiber-type distribution Old control: ↓ type I (slow oxidative) and IIa (fast oxidative glycolytic), ↑ type IIb (fast twitch glycolytic) Apigenin (50 mg/kg): ↑ type I and IIa, ↓ type IIb (*p* < 0.05)	**Muscle strength:** **Grip strength** Old control: ↓ vs. young Apigenin (50 mg/kg): ↑ vs. old control (*p* < 0.05) **Physical performance:** **Frailty index (FI)** Old control: ↑ FI with age (from 16 to 25 mo) Apigenin (50 mg/kg): ↓ FI vs. old control at 25 mo (*p* < 0.05) **Running distance** Old control: ↓ vs. young Apigenin (50 mg/kg): ↑ vs. old control (*p* < 0.05)	Fiber-type shift (see Muscle mass results) Apigenin promotes shift toward oxidative fibers (type I/IIa) and reduces glycolytic fibers (type IIb)	Mitochondrial respiration & energetics Apigenin (50 mg/kg): ↑ basal O_2_ consumption rate (State 4), ↑ activities of complexes I, II, IV, ↑ ΔΨm, ↑ ATP content (*p* < 0.05) Mitochondrial structure & biogenesis Apigenin (50 mg/kg): ↑ mitochondrial number, diameter, volume density (TEM), ↑ mtDNA copy number, ↑ protein expression of PGC-1α, NRF-1, TFAM, ATP5B (*p* < 0.05) Mitophagy & apoptosis Apigenin (50 mg/kg): ↓ BNIP3, ↓ p62, ↓ LC3-II/I ratio, ↓ Cyt-C release from mitochondria to cytosol, ↓ DNA fragmentation, ↓ PARP and caspase-3 activities (*p* < 0.05) Oxidative stress Apigenin (50 mg/kg): ↓ mitochondrial superoxide anion (O_2_ ^−^), ↓ H_2_O_2_, ↓ MDA, ↓ protein carbonyls (*p* < 0.05) Antioxidant defense Apigenin (50 mg/kg): ↑ T-AOC, ↑ SOD and GPx activities (*p* < 0.05)	Nine-month apigenin supplementation (50 mg/kg/day) to aged mice (starting at 16 mo) relieves age-related muscle atrophy, improves muscle function (grip strength, running distance), and reduces frailty. The beneficial effects are associated with reduced oxidative stress, enhanced antioxidant enzyme activities, improved mitochondrial respiratory function and biogenesis, and inhibition of hyperactive mitophagy and mitochondria-dependent apoptosis. Apigenin also promotes a shift from glycolytic (type IIb) to oxidative (type I/IIa) muscle fibers.
**Vays, 2014 [56]**	Wistar rat (male) and OXYS rat (male)	Young: 3 mo Old untreated: 24 mo Old treated: 24 mo (treatment from 19 to 24 mo) Mixed natural + accelerated aging	**Type:** SkQ1 (mitochondria-targeted antioxidant; 10-(6′-plastoquinonyl)decyltriphenylphosphonium) Vehicle: Mixed with standard rodent feed Route: Oral (with food) Dose: 250 nmol/kg body weight/day Frequency: Daily for 5 months (from 19 to 24 months of age)	*M. gracilis* Medial ventrum of *M. quadriceps femoris*	**N/A**	**N/A**	**N/A**	Mitochondrial network and ultrastructure (Wistar rats) Aging (24 mo vs. 3 mo): ↓ mitochondrial occupancy in isotropic region (38% → 24%); ↑ matrix electron density, compressed matrix, enlarged intermembrane space (de-energized state); disordered cristae; degradation of mitochondrial reticulum SkQ1: ↑ mitochondrial occupancy (24% → 33%); improved ultrastructure (intermediate/”twisted” energized state); preserved subsarcolemmal clusters and interfibrillar network OXYS rats (accelerated aging model) At 3 mo already: megamitochondria; cristae-free regions; reduced cristae number; ↓ mitochondrial occupancy (17% vs. Wistar 38%) Aging (24 mo): further ↓ occupancy (17% → 6%); complete destruction of mitochondrial reticulum; only small solitary mitochondria; extensive autophagosome-rich regions; myofibril lysis SkQ1: prevented network degradation; ↑ occupancy (6% → 11%); mitochondria exhibited “twisted” energized state; reduced autophagosome areas	Daily oral SkQ1 (250 nmol/kg) for 5 months starting at 19 months of age delays age-related sarcopenia-associated damage to mitochondrial ultrastructure in both normal (Wistar) and accelerated-aging (OXYS) rats. SkQ1 preserves the mitochondrial reticulum (subsarcolemmal clusters and interfibrillar network), increases mitochondrial volume density, and maintains cristae in an energized (“twisted”) state. It also prevents the appearance of megamitochondria, cristae-free regions, and autophagosome accumulation in OXYS rats. The effect may be partially mediated by activation of the GH/IGF-1 axis (reported in a related study).
**Mosoni, 2014 [47]**	Wistar rat (male)	Start: ~16 mo (69 wk) End: ~22 mo (96 wk) Treatment duration: 6 mo Natural aging	**Type:** Chamomile extract (with apigenin as marker), Vitamin E, Vitamin D (combined) Vehicle: Incorporated into pelleted diet Route: Oral dietary supplementation Dose: Chamomile extract 17 g/kg diet; Vitamin E 300 IU/kg diet; Vitamin D 5000 IU/kg diet Frequency: Continuous for 6 months	Gastrocnemius, tibialis anterior, extensor digitorum longus (EDL), soleus, epitrochlearis	Muscle weights (absolute) No significant effect of Aox+ supplementation on gastrocnemius, tibialis anterior, EDL, or soleus weights (*p* > 0.05) Lean body mass change Aox+ did not affect the age-related loss of lean body mass	**N/A**	In vivo muscle protein synthesis (gastrocnemius FSR) No significant effect of Aox+ Ex-vivo muscle protein synthesis (epitrochlearis) No significant effect of Aox+ in post-absorptive or post-prandial states Leucine-stimulated protein synthesis (ex-vivo) No significant effect of Aox+ Ex-vivo muscle proteolysis No significant effect of Aox+	Muscle oxidative damage TBARS (lipid peroxidation): ↓ with Aox+ (*p* < 0.01) Protein carbonyls: NS Liver antioxidant status Glutathione content: ↑ with Aox+ (*p* < 0.05) SOD activity: NS (except casein vs. whey effect) Plasma antioxidant capacity Total antioxidant capacity (TAC): ↑ with Aox+ (*p* < 0.01) Muscle antioxidant enzymes SOD, glutathione: NS	Six-month dietary supplementation with chamomile extract, vitamin E, and vitamin D (Aox+) in healthy old rats improved some oxidative stress markers (↓ muscle TBARS, ↑ liver glutathione, ↑ plasma TAC) and showed a trend toward reduced plasma α2-macroglobulin (inflammation). However, Aox+ did not affect muscle mass, lean body mass loss, muscle protein synthesis (basal or leucine-stimulated), or muscle proteolysis. In these healthy rats with low baseline inflammation, antioxidant supplementation above nutritional recommendations was ineffective at delaying sarcopenia.
**van Dijk, 2016 [54]**	C57BL/6J mouse (male)	Start: 18 mo After 4 mo: 22 mo After 3 mo intervention: 25 mo Natural aging + nutritional challenge	**Phase I (all lowox groups):** Low-antioxidant diet (vitamins A/E, Se, Zn at 25% of recommended intake) for 4 months. Phase II (3 months, from 22 to 25 mo, all groups continued lowox base except CTRL): CTRL: Standard AIN-93M diet (normal antioxidant levels) for entire 7 months. Lowox: Continued low-antioxidant diet. AOX: Lowox + supplementation with vitamins A (3×), E (25×), Se (13×), Zn (6.5×). PROT: Lowox + replacement of casein by leucine-enriched whey protein. TOTAL: Lowox + AOX + PROT. All diets were isocaloric and isonitrogenous. Route: Oral (dietary).	Tibialis anterior (TA), extensor digitorum longus (EDL), soleus (SOL), plantaris (PLANT), gastrocnemius (GM)	**Lowox vs. CTRL:** No difference in muscle mass (TA, EDL, SOL, PLANT, GM) at 22 or 25 m. Interventions vs. Lowox: AOX, PROT, TOTAL: no significant change in any muscle mas. TOTAL: ↑ whole-body lean mass *p* = 0.031), but not specific muscle weight.	**Muscle strength:** **Lowox vs. CTRL:** ↓ maximal grip strength (already at 22 mo, *p* < 0.00001). ↔ maximal ex vivo force. **Interventions vs. Lowox:** AOX, PROT, TOTAL: ↑ maximal grip strength ( *p* < 0.05). PROT & TOTAL: ↑ maximal ex vivo force (Figure 5C, *p* < 0.05). **Physical performance:** **Lowox vs. CTRL:** ↑ muscle fatigue (lower fatigue index, Figure 5E, *p* = 0.003). ↔ physical activity. **Interventions vs. Lowox:** AOX, PROT, TOTAL: ↓ muscle fatigue (fatigue index restored to CTRL level, *p* = 0.012). PROT: ↑ physical activity (slowed decline, *p* = 0.012).	**N/A**	**Oxidative stress (liver):** Lowox vs. CTRL: ↔ GSH at 22 and 25 m; ↑ MDA at 25 mo (*p* < 0.05); ↔ plasma vitamins A/E; trend ↓ hepatic vitamin E (*p* = 0.076). AOX & TOTAL: ↑ GS, ↓ MD, ↑ plasma and hepatic vitamins A/E (*p* < 0.05). Mitochondrial dynamics (EDL gene expression): Lowox vs. CTRL: ↔ at 22 or 25 mo individually, but a trend toward ↓ when ages combined (PCA, *p* = 0.079). AOX, PROT, TOTAL: ↑ mitochondrial dynamics (PCA, Figure 7A–C, *p* < 0.05). ↔ mitochondrial densit.	Long-term low antioxidant intake (25% of recommendation) in aged mice impairs muscle function (lower grip strength, increased fatigue) without changing muscle mass. Supplementation with antioxidants (AOX) improves oxidative status, reduces fatigue, and restores grip strength, associated with improved mitochondrial dynamics. Replacement of casein by leucine-enriched whey protein (PROT) improves muscle force, power, grip strength, and physical activity, also linked to better mitochondrial dynamics. Combination (TOTAL) gives additive benefits, increasing lean mass and further improving muscle quality. Neither protein source change nor antioxidant supplementation affected muscle mass; benefits are functional and quality-related.
**Takisawa, 2019 [57]**	SMP30-KO mouse (female)	Start: 8 wk End: 24 wk (AsA deficiency for 16 wk) Deficiency model	**Type:** L-Ascorbic acid (vitamin C, AsA) Vehicle: Dissolved in drinking water (containing 10 μM EDTA) Route: Oral (drinking water) Dose: 1.5 g/L (AsA^+^ group); AsA^−^ group received tap water without AsA; Recovery group: AsA^−^ for 12 wk then AsA^+^ for 12 wk Frequency: Ad libitum, continuous	Gastrocnemius, soleus, plantaris, tibialis anterior (TA), extensor digitorum longus (EDL)	**AsA^−^ vs. AsA^+^:** At 12 and 16 wk of treatment (i.e., 20 and 24 wk of age): ↓ weight of gastrocnemius, soleus, plantaris, TA, EDL (to 70-88% of control, *p* < 0.05) At 16 wk: ↓ CSA of soleus type I and IIa fibers (by 25-28%, *p* < 0.05, Figure 2b) Recovery (AsA^−^→^+^) vs. AsA^−^: Muscle weights restored to AsA^+^ levels	**Muscle strength:** **Grip strength (wire hanging)** AsA^−^ vs. AsA^+^: ↓ from 8 wk (*p* < 0.05) **Physical performance:** **Endurance (treadmill distance)** AsA^−^ vs. AsA^+^: ↓ from 4 wk of treatment (*p* < 0.05) **Home cage activity (diurnal/nocturnal)** AsA^−^ vs. AsA^+^: ↓ from 12 wk (*p* < 0.05) **Recovery vs. AsA^−^:** All functional parameters restored to AsA^+^ level	**AsA^−^ vs. AsA^+^ (TA muscle mRNA):** At 8-16 wk: ↑ FOXO1, Cblb (early) At 16 wk: ↑ atrogin1/MAFbx (418%), MuRF1 (268%), FOXO1 (193%) (*p* < 0.05) Also ↑ Nrf2, Cat, Aco2, GPx4, SOD1 (antioxidant genes) at later time points Recovery vs. AsA^−^: Expression of most genes (except MuRF1) restored to AsA^+^ levels	**AsA^−^ vs. AsA^+^:** Muscle AsA content at 4 wk: 0.7% of control (*p* < 0.05) Muscle ROS levels: ↔ at 4 wk; ↑ 2-fold at 8 wk (*p* < 0.05) Muscle protein carbonyls: ↔ at 4 wk; ↑ 1.5-fold at 8 wk (*p* < 0.05) Recovery vs. AsA^−^: AsA content restored; ROS levels restored; protein carbonyls partially restored	Long-term vitamin C deficiency in SMP30-KO mice (which cannot synthesize AsA) causes significant muscle atrophy (↓ muscle weight and fiber CSA) and impairs physical performance (endurance, grip strength, activity). The muscle wasting is associated with upregulation of ubiquitin ligases (atrogin1/MAFbx, MuRF1) and FOXO1, indicating activation of the ubiquitin-proteasome pathway. Oxidative stress (↑ ROS, ↑ protein carbonyls) appears after 8 wk of deficiency. Re-supplementation of AsA for 12 wk fully restores muscle mass and function, and normalizes most gene expression changes.
**Sinha-Hikim, 2013 [43]**	C57BL/6J mouse (male)	Young: 5 mo Old: 23 mo (treatment from 17 to 23 mo) Natural aging	**Type:** F1 (cystine-based glutathione precursor; contains L-cystine, glycine, selenomethionine, L-glutamine) Vehicle: Mixed into mouse chow diet pellets Route: Oral (dietary) Dose: 3 mg/kg food Frequency: Daily for 6 months	Gastrocnemius	**Muscle weight** **Old vs. young:** ↓ by 17.3% (*p* < 0.05) Old + F1 vs. old: ↑ to young level (*p* < 0.05) Relative muscle weight (mg/100g body weight) Old + F1: ↑ vs. old (0.35 → 0.44), but still lower than young (0.62) Muscle fiber cross-sectional area (CSA) Old vs. young: ↓ (*p* < 0.001) Old + F1 vs. old: ↑ to young level (*p* < 0.001)	**N/A**	**AMPK signaling** **p-AMPK:** old ↓ vs. young; old + F1 ↑ to young level Lipogenesis FAS: old ↑ vs. young; old + F1 ↓ to young level G6PD: old ↓ vs. young; old + F1 ↑ to young level Apoptosis signaling p-JNK: old ↑ vs. young; old + F1 ↓ to young level Muscle cell apoptosis (TUNEL): old ↑; old + F1 fully prevented (to 0%) Notch/Akt signaling Delta1: old ↓ vs. young; old + F1 ↑ to young level p-Akt: old ↓ vs. young; old + F1 ↑ to young level	**Oxidative stress** **GSH/GSSG ratio:** old ↓ vs. young; old + F1 ↑ to young level 4-HNE (lipid peroxidation): old ↑ vs. young; old + F1 ↓ to below young level SOD2: old vs. young no change; old + F1 ↑ vs. old (*p* < 0.05) SOD1: no change	Six-month dietary supplementation with F1 (a cystine-based antioxidant) to middle-aged (17 mo) mice prevents age-related loss of muscle mass and fiber size, restoring them to young levels. F1 reduces oxidative stress (↑ GSH/GSSG, ↓ 4-HNE), suppresses JNK-mediated muscle cell apoptosis, and restores AMPK activation, leading to reduced lipogenesis (↓ FAS) and improved lipid metabolism. F1 also enhances Notch (Delta1) and Akt signaling, promoting muscle growth and survival. These results suggest that F1 may be an effective intervention to delay sarcopenia.
**Joseph, 2013 [53]**	Fischer 344 × Brown Norway hybrid rat (male)	27 mo (≈117 wk) at start; 6 wk treatment, endpoint ≈28.5 mo Natural aging	**Type:** Resveratrol (RSV) (50 mg/kg/day) alone, or combined with 20% caloric restriction (CR + RSV). Also CR alone (20% reduction) and ad-libitum control (AL). Vehicle: Bacon-flavored tablet mixed with normal food pellets Route: Oral (dietary) Dose: RSV: 50 mg/kg body weight/day; CR: 20% reduction of AL intake Frequency: Daily for 6 weeks	White gastrocnemius (WG), red gastrocnemius (RG), tibialis anterior (TA), soleus, plantaris	**Absolute muscle weight:** CR+RSV vs AL: ↓ gastrocnemius wet weight (*p* < 0.05) No other significant differences in absolute weights Relative muscle weight (mg/g body weight): CR vs. AL: ↑ gastrocnemius (*p* < 0.05) and soleus (*p* < 0.05) CR+RSV vs AL: ↑ soleus (*p* < 0.05) and plantaris (*p* < 0.05) RSV alone: no significant effect on any relative muscle weight	**N/A**	**RSV-specific (vs. AL):** Sirt1 protein in WG: ↑ 2.6-fold (*p* < 0.05) AIF protein in RG: ↑ 1.5-fold (*p* < 0.05) Bax protein in WG: ↑ (*p* < 0.05) Bcl-2 protein in WG: ↑ (*p* < 0.05) Bax/Bcl-2 ratio: no change p-AMPK/AMPK ratio: no change DNA fragmentation (apoptotic index): no change CR-specific (vs. AL): PGC-1α protein in WG: ↑ 2-fold (*p* < 0.05) MnSOD protein in WG: ↑ 1.7-fold (*p* < 0.05) p-AMPK/AMPK ratio: no change DNA fragmentation: no change CR+RSV vs AL: No additive/synergistic effects; PGC-1α in WG was lower than CR or RSV alone	Mitochondrial function (TA permeabilized fibers): State 3/State 4 respiration, RCR: no significant differences among groups COX activity (WG, RG): No significant differences Cytochrome c protein (WG, RG): No significant differences MnSOD protein: CR ↑ in WG (see Mechanisms); RSV no effect	Six-week late-life 20% caloric restriction (CR) modestly protects against age-related loss of relative muscle mass (gastrocnemius, soleus, plantaris) in aged rats, associated with increased PGC-1α and MnSOD in glycolytic (white) muscle. Resveratrol (RSV) alone did not improve muscle mass, but altered Sirt1, AIF, Bax, and Bcl-2 in a fiber-type-specific manner without affecting apoptotic index or mitochondrial respiration. Combined CR+RSV did not provide additive benefits; some proteins were blunted compared to single treatments. Glycolytic muscle was more responsive to interventions than oxidative muscle.
**Toniolo, 2018 [44]**	C57BL/6 mouse (male)	Start: 12 mo End: 18 mo (treatment 6 mo) Natural aging	**Type:** Resveratrol (RES) Vehicle: Mixed in standard chow diet Route: Oral (dietary) Dose: 0.04% (*w*/*w* in diet); estimated ~50–55 mg/kg body weight/day Frequency: Continuous for 6 months	Tibialis anterior (TA), gastrocnemius, extensor digitorum longus (EDL), soleus	**Body weight:** no significant difference EDL and soleus muscle weights: no significant difference Mean myofiber CSA (TA): ↓ by 8.6% (*p* < 0.0001) Fiber size distribution: increased proportion of small/medium-small fibers, decreased large fibers	**Muscle strength:** **Ex vivo contractile properties (EDL & soleus)** Twitch tension: no change (Figure 2A) Tetanus tension: no change (Figure 2B) Time to peak: no change (Figure 2C) Half-relaxation time: no change (Figure 2D) **Physical performance:** **Resistance to fatigue** ↑ in both EDL and soleus (*p* < 0.05, Figure 2E) **In vivo (treadmill endurance to exhaustion)** No significant difference (Figure 2F)	**Tubular aggregates (TAs,** **TA muscle):** Number of TAs ↓ by 26% (*p* < 0.05) Inflammatory marker: Cox II protein expression ↓ (*p* < 0.05) Myosin heavy chain isoforms (gastrocnemius): MyHCIIB: ↑ (*p* < 0.05) MyHCIIA/IIX: ↓ (*p* < 0.05) MyHCI: no change SERCA1 (fast isoform): ↑ (*p* < 0.05)	**Succinate dehydrogenase (SDH) activity (TA):** ↑ by 10% (*p* < 0.05) Mitochondrial markers (gastrocnemius): Tom20 (mitochondrial volume): no change Cox IV (mitochondrial activity marker): no change	Six-month dietary resveratrol (0.04%) supplementation in middle-aged mice (starting at 12 mo) reduces the age-related appearance of tubular aggregates and improves ex vivo muscle resistance to fatigue without affecting muscle mass or treadmill endurance. RES induces a shift toward a more oxidative metabolism (↑ SDH activity) and alters myosin heavy chain isoform distribution (↑ MyHCIIB, ↓ IIA/IIX). The anti-inflammatory effect (↓ Cox II) may contribute to the reduction of tubular aggregates. RES may be a good candidate to reduce some age-related skeletal muscle alterations.
**Chen, 2024 [28]**	C57BL/6J mouse (male)	Young: ~6 mo Old: ~22 mo (treatment 4 weeks, endpoint ~23 mo) Natural aging	**Type:** Polygonatum sibiricum polysaccharide (PSP) Vehicle: Dissolved in saline Route: Intraperitoneal injection Dose: 200 mg/kg body weight Frequency: Once daily for 4 weeks	Extensor digitorum longus (EDL), gastrocnemius (GA), quadriceps (QA), tibialis anterior (TA), soleus (SOL)	**Relative muscle weight (mg/g body weight):** Old control vs. young: ↓ QA, GA, TA, SOL Old + PSP vs. old control: ↑ QA, GA, TA, SOL (*p* < 0.05) Myofiber CSA (TA): Old control vs. young: ↓ mean CSA Old + PSP vs. old control: ↑ mean CSA (*p* < 0.05) Fiber size distribution: rightward shift (more large fiber)	**Muscle strength:** **Grip strength:** Old control vs. young: ↓ Old + PSP vs. old control: ↑ (*p* < 0.05) **Physical performance:** **Hanging time:** Old + PSP vs. old control: ↑ (*p* < 0.05) **Rotarod (max speed):** Old + PSP vs. old control: ↑ (*p* < 0.05) **Treadmill running distance:** Old + PSP vs. old control: ↑ (*p* < 0.05)	**N/A**	**Oxidative stress:** ROS (DHE staining): old control ↑ vs. young; old + PSP ↓ vs. old control (*p* < 0.05) T-AOC, SOD, CAT activities: old control ↓ vs. young; old + PSP ↑ vs. old control (*p* < 0.05) Mitochondrial calcium overload (Rhod-2): old + PSP ↓ vs. old control MAM formation (VDAC/GRP78 colocalization): old + PSP ↓ vs. old control MAM/calcium-related proteins (muscle tissue): GRP75, MCU: old control ↑ vs. young; old + PSP ↓ vs. old control	Four weeks of PSP (200 mg/kg/day i.p.) in aged mice (starting at 22 mo) improves muscle mass (increases relative muscle weights and fiber CSA) and multiple functional parameters (grip strength, hanging time, rotarod speed, treadmill endurance). PSP reduces oxidative stress (↓ ROS, ↑ T-AOC, SOD, CAT) and attenuates mitochondrial calcium overload, MAM formation, and the expression of MAM-related proteins (GRP75, MCU). The proposed mechanism involves regulation of calcium homeostasis via MAM, leading to improved mitochondrial function and anti-aging effects in skeletal muscle.
**Wang, 2023 [58]**	C57BL/6J mouse (male)	Start: 8 wk End: ~18 wk (treatment 10 wk) Chemically induced aging-like model	**Type:** Nobiletin (Nob) Vehicle: DMSO dissolved in 0.9% saline Route: Oral gavage Dose: 100 mg/kg body weight Frequency: Once daily for 10 weeks	Gastrocnemius (Gas), soleus (Sol), tibialis anterior (TA)	**Body weight and gain:** D-gal vs. CK: ↓ BW and BW gain (*p* < 0.05) D-gal+Nob vs. D-gal: ↑ BW and BW gain (*p* < 0.05) Relative muscle weight (Gas/BW, Sol/BW): D-gal vs. CK: ↓ D-gal+Nob vs. D-gal: ↑ (*p* < 0.05) Lean mass (lean/BW): D-gal+Nob vs. D-gal: ↑ (*p* < 0.05) Myofiber CSA (TA): D-gal vs. CK: ↓ D-gal+Nob vs. D-gal: ↑ (*p* < 0.05) Myofiber ultrastructure: D-gal: disorganized sarcomere structure (I band, A band, Z line, M line disrupted) D-gal+Nob: structure restored	**Muscle strength:** **Grip strength:** D-gal vs. CK: ↓ D-gal+Nob vs. D-gal: ↑ (*p* < 0.05) **Physical performance:** **Running time (muscular endurance):** D-gal vs. CK: ↓ D-gal+Nob vs. D-gal: ↑ (*p* < 0.05)	**Protein synthesis (mTOR/Akt pathway):** D-gal vs. CK: ↓ p-Akt/Akt, p-mTOR/mTOR, p-p70 S6K D-gal+Nob vs. D-gal: ↑ all (*p* < 0.05) Protein degradation (FOXO-MAFbx/MuRF1 pathway): D-gal vs. CK: ↑ p-FOXO3a/FOXO3a, ↑ MAFbx, ↑ MuRF1 D-gal+Nob vs. D-gal: ↓ all (*p* < 0.05) Inflammation (serum): D-gal vs. CK: ↑ LBP, TNF-α, IL-6 D-gal+Nob vs. D-gal: ↓ all (*p* < 0.05)	**N/A**	Ten-week oral Nobiletin (100 mg/kg/day) treatment in D-galactose-induced aging mice (8-wk start) significantly improves body weight, muscle mass (Gas, Sol, lean mass), myofiber CSA and ultrastructure, and muscle function (running endurance, grip strength). Nob activates protein synthesis via the Akt/mTOR/p70 S6K pathway and inhibits protein degradation by suppressing FOXO3a-mediated MAFbx/MuRF1 expression and reducing systemic inflammation (↓ LBP, TNF-α, IL-6). Nob regulates protein homeostasis to counteract D-gal-induced skeletal muscle atrophy, suggesting its potential as a candidate for preventing/treating age-associated muscle loss.
**Jackson, 2011 [42]**	C57BL/6 mouse (male)	Young: 3 mo Middle-aged: 18 mo (start of treatment) Aged: 28 mo (end of treatment, 10 mo supplementation) Natural aging	**Type:** Resveratrol (trans-resveratrol) Vehicle: Mixed into standard rodent chow (AIN-76A based) Route: Oral (dietary) Dose: 0.05% (*w*/*w*) in diet; estimated intake: 60 mg/kg/day at 18 mo, 46 mg/kg/day at 28 mo Frequency: Continuous for 10 months	Plantaris, gastrocnemius, vastus lateralis	**Gastrocnemius and plantaris wet weights:** Old control vs. young: ↓ (*p* < 0.05) Old + resveratrol vs. old control: no significant difference (*p* > 0.05) Muscle weight normalized to body weight: Same pattern; no protective effect of resveratrol	**Muscle strength:** **Ex vivo isometric contractility (plantaris):** Force-frequency curve: rightward shift in resveratrol group vs. old control (faster phenotyp) Contraction time (CT): ↓ in resveratrol vs. old control (34.7 ± 0.8 vs. 39.6 ± 1.4 ms, *p* < 0.05, Figure 6B) Half-relaxation time (½ RT): no change Twitch-to-tetanus ratio (Pt/Po): no change **Physical performance:** **Fatigue index** No change	**Sirt1:** Activity: old control ↑ vs. young; resveratrol ↑ further (*p* < 0.05) Protein content: old control ↑ vs. young; resveratrol showed trend (*p* = 0.063) FOXO3a: protein content unchanged by age or resveratrol	**Oxidative stress markers (gastrocnemius):** H_2_O_2_: old control ↑ vs. young; resveratrol ↓ vs. old control (*p* < 0.05), but still > young (Figure 5A) Lipid peroxidation (MDA/HAE): old control ↑ vs. young; resveratrol ↓ vs. old control (*p* < 0.05), partially attenuated **Protein carbonyls:** old control ↑ vs. young; resveratrol no effect (Figure 5C) Antioxidant enzymes (vastus lateralis): MnSOD activity: old control vs. young no change; resveratrol ↑ vs. old control (*p* < 0.05) CuZnSOD activity: old control ↑ vs. young; resveratrol ↓ to young level (*p* < 0.05) MnSOD and CuZnSOD protein: aging ↑; resveratrol no effect Mitochondrial markers: PGC1α protein: no change **Citrate synthase activity:** old control ↓ vs. young; resveratrol no effect **Cytochrome c in cytosol:** old control ↑ vs. young; resveratrol no improvement	Ten-month dietary resveratrol (0.05%) in aged mice (starting at 18 mo) improves some markers of oxidative stress (↓ H_2_O_2_, ↓ lipid peroxidation, ↑ MnSOD activity, normalizes CuZnSOD activity) but does not prevent age-related loss of muscle mass (sarcopenia). Resveratrol shifts muscle contractile properties toward a faster phenotype (rightward force-frequency curve, shorter contraction time) without affecting fatigue resistance. The intervention increases Sirt1 activity but does not enhance mitochondrial biogenesis (PGC1α, citrate synthase, mitochondrial cytochrome c release unchanged). Overall, resveratrol has a protective effect against age-induced oxidative stress in skeletal muscle but does not attenuate sarcopenia.
**Chang, 2024 [26]**	SAMP8 mouse (male)	Young: 12 wk Old control: 40 wk (at sacrifice) Old + Olg: treated from 32 to 40 wk Accelerated-aging model	**Type:** Oligonol® (Olg) (low-molecular-weight polyphenol mixture) Vehicle: Mixed in standard chow diet Route: Oral (dietary) Dose: 200 mg/kg chow diet Frequency: Continuous for 8 weeks	Gastrocnemius, soleus	**N/A**	**N/A**	**BCAA transport & catabolism:** Sarcolemma LAT1 protein: ↑ (vs. old control, *p* < 0.05) Total BCAT2 protein: ↑ (vs. old control, *p* < 0.05) mTOR signaling (in vitro validation): Olg + BCAAs synergistically ↑ p-mTOR/p70S6K; effect blocked by LAT1 inhibitor JPH203 **BCAA concentrations:** Gastrocnemius BCAA: ↑ (vs. old control) Serum BCAA: ↓ (vs. old control)	**N/A**	Olg supplementation for 8 weeks in pre-sarcopenic SAMP8 mice increases the expression of the BCAA transporter LAT1 on the sarcolemma and the catabolic enzyme BCAT2 in skeletal muscle. These changes are associated with a shift of BCAAs from circulation into muscle, and in vitro experiments confirm that Olg enhances BCAA-stimulated mTOR/p70S6K signaling in a LAT1-dependent manner. The findings suggest that Olg may improve anabolic sensitivity to BCAAs during aging by restoring BCAA transport and metabolism.
**Javadov, 2015 [52]**	Fischer Brown Norway (F344/BN) rat (male)	Adult: 5 mo Old: 29 mo (treatment from 28 to 29 mo, 4 weeks) Natural aging	**Type:** XJB (mitochondria-targeted ROS/electron scavenger) Vehicle: Dissolved in sunflower seed oil Route: Intraperitoneal injection Dose: 3 mg/kg body weight Frequency: 3 times per week for 4 weeks	Soleus, gastrocnemius, plantaris	**Body weight:** no effect Muscle weight: Plantaris: no change Gastrocnemius: no change Soleus: ↑ by 14.9% (*p* < 0.05) Muscle-to-body weight ratio: Soleus: ↓ by 10.3% (*p* < 0.05) Gastrocnemius & plantaris: no change Single fiber CSA (gastrocnemius): no change	**Muscle strength:** **Single muscle fiber (gastrocnemius):** Maximal unloaded shortening velocity (V_0_): ↑ by 35% (*p* < 0.01,) Absolute power: ↑ by 58% (*p* < 0.01) Maximum force (P_0_): no change Specific force (P_0_/CSA): no change Normalized power: no change	**N/A**	**Mitochondrial ETC complex activity:** Complex I: ↑ in soleus (69%, *p* < 0.05) and plantaris (60%, *p* < 0.05); gastrocnemius NS Complex III: ↑ in soleus (39%, *p* < 0.05) and gastrocnemius (59%, *p* < 0.05); plantaris NS Complex IV: ↑ in soleus (83%, *p* < 0.01), gastrocnemius (70%, *p* < 0.05), plantaris (44%, *p* < 0.05) **Mitochondrial mass (citrate synthase):** no change ETC supercomplex levels: no change vs. aged control SOD2 protein: no change Oxidative damage: Protein carbonyls (gastrocnemius homogenate): aged ↑ vs. adult; XJB ↓ vs. aged control (*p* < 0.05) Protein carbonyls (mitochondria): aged ↑ vs. adult; XJB ↓ vs. aged control (*p* < 0.01)	Four weeks of XJB treatment in aged rats (starting at 28 mo) does not affect body weight or most muscle weights (except soleus weight increased). XJB improves single muscle fiber contractile properties (↑ V_0_, ↑ absolute power) without changing fiber size. These improvements are associated with increased activity of mitochondrial ETC complexes (I, III, IV) in a muscle-specific manner, without changes in mitochondrial mass or ETC supercomplex assembly. XJB reduces protein carbonylation (oxidative damage) in muscle homogenate and mitochondria. The study supports a causal role for mitochondrial ROS in age-related muscle weakness and suggests that mitochondrial-targeted ROS scavengers can reverse these deficits.
**Kanazawa, 2026 [31]**	ICR mouse (male)	4-wk-old mice; hindlimb unloading for 14 day Disuse-induced atrophy	**Type:** Edaravone-loaded human serum albumin nanoparticles (HSA-NPs; SPARC-binding injured muscle-targeted DDS / imDDS) **Model:** Hindlimb unloading (HU) for 14 days **Control:** Saline; free edaravone control **Vehicle/carrier:** Human serum albumin nanoparticles **Route:** Intravenous tail vein injection **Dose:** HSA-NPs [3 mg edaravone/kg]; free edaravone 3 mg/kg **Frequency:** Once daily for 4 days, days 10–13 after HU initiation; endpoint day 14	Tibialis anterior, gastrocnemius, soleus Tibialis anterior for SPARC, myostatin, atrogin-1, MDA, PGC-1α	**Hindlimb muscle weight** HU caused time-dependent muscle weight loss vs. day 0 (*p* < 0.05) **Tibialis anterior & gastrocnemius weight** **Free edaravone:** no significant protection against HU-induced loss **HSA-NPs:** ↑ / preserved muscle weight vs. HU saline (*p* < 0.05) **Soleus weight** **HSA-NPs:** tendency to increase vs. HU saline, not clearly significant **Myofiber CSA** N/A	**Physical performance:** **Treadmill running distance** **HU saline vs. control:** ↓ running distance **HSA-NPs vs** **HU saline:** ↑ running distance (*p* < 0.05) **Free edaravone:** no significant improvement	**SPARC targeting** HU increased SPARC expression in tibialis anterior during muscle atrophy (*p* < 0.05) Cy5-labeled HSA-NPs accumulated preferentially in injured hindlimb muscle and co-localized with SPARC-positive myocytes **Atrophy-related signaling** **Myostatin:** HU ↑ vs. control; HSA-NPs ↓ vs. HU saline (*p* < 0.05) **Atrogin-1:** HU ↑ vs. control; HSA-NPs tended to ↓ vs. HU saline (*p* = 0.09, NS) **Free edaravone:** no effect on myostatin or atrogin-1	**Muscle MDA** **HU saline vs. control:** ↑ **HSA-NPs vs** **HU saline:** ↓ (*p* < 0.05) **Free edaravone:** NS **PGC-1α** **HU saline vs. control:** ↓ HSA-NPs prevented HU-induced downregulation / ↑ vs. HU saline (*p* < 0.05) **Muscle injury marker** **Plasma CPK:** HSA-NPs ↓ vs. HU saline (*p* < 0.05) **Safety** **Organ coefficients:** no significant difference **Liver, kidney, spleen histology:** no obvious abnormality **AST, ALT, BUN,** **SCr:** no significant difference among groups	SPARC-guided edaravone-loaded HSA-NPs reversed HU-induced skeletal muscle atrophy, whereas free edaravone did not. The treatment improved both muscle mass and treadmill endurance. The effect is associated with injured-muscle targeting, reduced MDA, reduced myostatin, a trend toward lower atrogin-1, and restored PGC-1α. Short-term systemic i.v. treatment showed no obvious liver/kidney/spleen toxicity. Limitation: model used young mice with acute HU-induced atrophy rather than naturally aged sarcopenia.
**Jia, 2026 [33]**	C57BL/6N mouse (male)	Start: 4 wk old D-gal-induced aging/sarcopenia model for 10 wk Endpoint: ~14 wk old Chemically induced aging-like model	**Type:** Ginsenoside Ro (GRo) **Model:** D-galactose-induced aging/sarcopenia **Positive control:** NMN **Vehicle:** Normal saline for control/model; GRo vehicle not clearly specified **Route:** D-gal intraperitoneal injection; GRo oral gavage; NMN oral gavage **Dose:** D-gal 200 mg/kg; GRo 20 or 40 mg/kg; NMN 100 mg/kg **Frequency:** D-gal daily for 10 wk; GRo/NMN from week 3 to week 10	Gastrocnemius (GA), soleus (SOL), tibialis anterior (TA), extensor digitorum longus (EDL) GA for histology, CSA, fibrosis, oxidative stress	**Body weight** No significant difference in body weight gain among groups **Food and water intake:** no significant difference **Muscle weight** **D-gal vs. control:** GA ↓ from 307.07 ± 22.23 to 261.31 ± 19.21 mg (*p* < 0.05) **D-gal vs. control:** TA ↓ from 100.67 ± 11.19 to 89.87 ± 4.46 mg (*p* < 0.05) **SOL and EDL:** no significant change **GA index:** D-gal ↓; GRo ↑ vs. D-gal (*p* < 0.05) **GA myofiber CSA** **Control:** 2065.17 ± 196.36 μm^2^ **D-gal:** 1209.34 ± 89.76 μm^2^, −41.44% vs. control **GRo:** 1686.56 ± 90.68 μm^2^ (↑ vs. D-gal) High-dose GRo increased the proportion of large fibers >2000 μm^2^ **Myofiber number** No significant difference among groups **Fibrosis** D-gal ↑ Sirius Red-positive collagen deposition GRo ↓ Sirius Red-positive area (*p* < 0.05)	**Muscle strength:** **Grip strength** **D-gal vs. control:** ↓ 19.38% GRo restored grip strength to near-control level **Physical performance:** **Treadmill test** **D-gal vs. control:** running time ↓ 21.12%, running distance ↓ 32.63%, maximum speed ↓ 17.29% GRo restored running time, distance, and maximum speed vs. D-gal (*p* < 0.05) **Rotarod** D-gal impaired rotarod performance GRo restored motor coordination to near-control level	**Direct protein metabolism signaling** N/A; Akt/mTOR/FoxO/MuRF1/Atrogin-1 not measured in vivo **Gut-muscle axis** D-gal reduced microbial richness and altered β-diversity GRo restored microbial richness, Shannon index, and Pielou evenness GRo shifted gut microbiota profile closer to control GRo enriched beneficial taxa including Monoglobaceae/Monoglobus, Peptococcaceae, Saccharimonadaceae, Colidextribacter, Akkermansiaceae/Akkermansia Akkermansia, Bifidobacterium, Akkermansiaceae, and Bifidobacteriaceae were positively correlated with GA mass, SOD, GSH, and ATP	**In vitro C2C12 myotubes** D-gal ↑ ROS 20 μM GRo ↓ intracellular ROS by ~22.3% **20 μM** **GRo restored mitochondrial membrane potential:** red/green JC-1 ratio ↑ 73.77% vs. D-gal 20 μM GRo ↑ mitochondrial fluorescence by 46.36% vs. D-gal GRo dose-dependently restored ATP content **In vivo GA muscle** D-gal ↑ MDA; GRo ↓ MDA D-gal ↓ SOD and GSH; GRo ↑ SOD and GSH D-gal ↓ skeletal muscle ATP; GRo restored ATP dose-dependently **Serum inflammatory cytokines** D-gal ↑ TNF-α, IL-6, IL-1β GRo reduced TNF-α by 40.26%, IL-6 by 35.94%, and IL-1β by 46.49%	GRo alleviated D-gal-induced sarcopenia-like phenotypes by improving GA mass, GA index, CSA, fibrosis, endurance, coordination, and grip strength. Benefits were associated with reduced oxidative stress, improved antioxidant capacity, restored ATP, and reduced systemic inflammation. GRo modulated gut microbiota composition and enriched taxa positively correlated with muscle mass and antioxidant/energy metabolism markers. Direct protein synthesis/degradation signaling was not assessed, so the main proposed mechanisms are oxidative stress, inflammation, mitochondrial function, and gut-muscle axis regulation.
**Lim, 2026 [32]**	C57BL/6 mouse (male)	Age: N/A / not reported Dex-induced acute muscle atrophy model for 10 days Experimentally induced atrophy	**Type:** Amentoflavone (AMF; biflavonoid from Ginkgo biloba) **Model:** Dexamethasone-induced muscle atrophy **Vehicle:** AMF suspended in 0.5% sodium carboxymethyl cellulose (CMC-Na); Dex in 30% PEG400 saline **Route:** AMF oral administration; Dex intraperitoneal injection **Dose:** AMF 1 or 10 mg/kg; Dex 25 mg/kg **Frequency:** AMF once daily for 11 days; Dex once daily for 10 days	Gastrocnemius (GAS), quadriceps (QUAD)	**Body weight** Dex caused significant body-weight loss beginning day 7 (*p* ≤ 0.05) AMF LD/HD maintained body weight comparable to control **Muscle weight** Dex ↓ GAS and QUAD muscle weights vs. control (*p* ≤ 0.001) **AMF ↑** **GAS weight vs. Dex:** LD and HD (*p* ≤ 0.01) **AMF ↑** **QUAD weight vs. Dex:** LD (*p* ≤ 0.05), HD (*p* ≤ 0.001) **Myofiber CSA** Dex ↓ GAS and QUAD CSA vs. control (*p* ≤ 0.001) AMF preserved/increased CSA vs. Dex **GAS CSA:** LD ↑ vs. Dex (*p* ≤ 0.001), HD ↑ vs. Dex (*p* ≤ 0.05) **QUAD CSA:** LD/HD ↑ vs. Dex (*p* ≤ 0.001) **MYH-positive fibers** Dex ↓ **MYH-positive fibers** AMF restored **MYH-positive fibers**	**Muscle strength:** **Grip strength** Dex ↓ vs. control AMF LD/HD ↑ vs. Dex (*p* ≤ 0.01) **Physical performance:** **Rotarod retention time** Dex ↓ vs. control (*p* ≤ 0.01) AMF LD/HD ↑ vs. Dex (*p* ≤ 0.05) **Muscle CK activity** Dex ↓ CK activity in GAS and QUAD AMF restored CK activity **GAS:** LD ↑ (*p* ≤ 0.001), HD ↑ (*p* ≤ 0.01) **QUAD:** LD/HD ↑ (*p* ≤ 0.05)	**MSTN binding / upstream validation** **In silico:** AMF showed strong predicted binding to MSTN (binding energy −8.20 kcal/mol) **CETSA:** AMF increased MSTN thermal stability, supporting AMF-MSTN interaction **Atrophy signaling in vivo** Dex ↑ Atrogin-1 and MuRF1 in GAS and QUAD AMF ↓ Atrogin-1 and MuRF1 **Myogenic markers** Dex ↓ MYF5 in GAS and MYOD in QUAD AMF partially restored these markers MYOG and MYH returned toward control levels in both tissues **MSTN-SMAD signaling** Dex ↑ MSTN and p-SMAD3 AMF ↓ MSTN and p-SMAD3 p-SMAD2 recovery mainly in Dex+HD group **AKT/mTOR** Dex ↓ p-AKT and p-mTOR AMF restored p-AKT and p-mTOR to varying degrees depending on muscle type	**In vivo animal muscle** Direct mitochondrial function or oxidative stress markers were not clearly reported in the main animal results **In vitro C2C12 / human MSC data** AMF ↓ intracellular ROS by >10% in C2C12 cells and >20% in human MSCs AMF ↑ NRF2 and SOD2 mRNA/protein expression These data suggest antioxidant contribution, but in vivo oxidative endpoints were not directly demonstrated in the main animal figures	AMF prevented Dex-induced body-weight and muscle-weight loss and improved GAS/QUAD CSA. AMF improved grip strength, rotarod endurance/coordination, and CK activity. The main in vivo mechanism is inhibition of MSTN-SMAD signaling, suppression of Atrogin-1/MuRF1, restoration of myogenic markers, and activation of AKT/mTOR anabolic signaling. Antioxidant effects were supported mainly by in vitro data. Limitation: mouse age was not reported and the model is acute Dex-induced atrophy rather than natural aging sarcopenia.
**He, 2025 [29]**	BALB/c mouse (male)	6-8 wk Body weight: 20 ± 2 g Experimentally induced atrophy	**Type:** Ferulic acid (FA) **Model:** Dexamethasone (Dex)-induced sarcopenia / muscle atrophy **Comparator:** 10,12-Tricosadiynoic acid (TA; ACOX1 inhibitor) **Vehicle:** Saline for control/model groups **Route:** Dex intraperitoneal injection; FA oral gavage **Dose:** Dex 20 mg/kg/day; FA 100 mg/kg/day; TA 100 μg/kg/day **Frequency:** Dex daily for 10 days, followed by FA or TA gavage daily for 10 days	Gastrocnemius (GAS)	**Body weight** Dex ↓ vs. NC after 10-20 days FA ↑ vs. Dex (*p* < 0.05) **Relative daily food intake** No significant difference among groups **Gastrocnemius individual muscle fiber area** Dex ↓ vs. NC (*p* < 0.001) FA ↑ vs. Dex (*p* < 0.01) TA ↑ vs. Dex (*p* < 0.05) **Absolute muscle weight** N/A	**Muscle strength:** **Grip strength** Dex ↓ vs. NC (*p* < 0.01) FA ↑ vs. Dex (*p* < 0.05) TA showed ↑ trend vs. Dex, but NS	**ACOX1-related mechanism** Dex ↑ ACOX1 mRNA in GAS (*p* < 0.001) FA ↓ ACOX1 mRNA vs. Dex (*p* < 0.0001) Dex ↑ ACOX1 protein (*p* < 0.01) FA ↓ ACOX1 protein vs. Dex (*p* < 0.05) Dex ↑ ACOX1 enzyme activity (*p* < 0.001) FA ↓ ACOX1 enzyme activity vs. Dex (*p* < 0.05) **Ubiquitin-proteasome pathway** **Atrogin-1 mRNA:** Dex ↑ vs. NC (*p* < 0.001); FA ↓ vs. Dex (*p* < 0.0001) **MuRF1 mRNA: FA showed ↓ trend vs. Dex; protein band showed reduced expression, but quantitative** ***p*** **value not clearly provided** **Myogenic differentiation markers** **MyoD mRNA:** FA ↑ vs. Dex (*p* < 0.01) **MHC-1 mRNA:** FA showed ↑ trend vs. Dex Western blot trends for Atrogin-1, MuRF1, MHC-1, and MyoD were consistent with mRNA results **Inflammatory factors in GAS** **TNF-α:** Dex ↑ vs. NC (*p* < 0.01); FA ↓ vs. Dex (*p* < 0.01) **IL-6:** Dex ↑ vs. NC (*p* < 0.01); FA showed ↓ trend vs. Dex, but NS	**Peroxisomal oxidative stress** FA inhibited ACOX1 expression and enzyme activity in GAS FA reduced ACOX1 immunofluorescence density vs. Dex (*p* < 0.01) **Catalase** **Catalase density in GAS:** no significant difference among groups **In vitro ROS** Dex ↑ ROS in C2C12 cells FA, especially 200 μg/ml, ↓ ROS vs. Dex **NRF2 pathway** FA did not significantly induce NRF2 nuclear translocation or HO-1/NQO1 expression, suggesting NRF2 was not the primary mechanism	FA alleviated Dex-induced sarcopenia-like muscle atrophy in mice, improving body weight, grip strength, and gastrocnemius fiber area. The main target identified was peroxisomal ACOX1, linking fatty acid β-oxidation-derived ROS to muscle atrophy. FA suppressed ACOX1 expression/activity, reduced peroxisomal oxidative stress, and improved redox homeostasis. FA inhibited protein degradation mainly through ↓ Atrogin-1 and reduced MuRF1 expression trend, while promoting myogenic differentiation marker MyoD. Anti-inflammatory effects were evident for TNF-α, while IL-6 showed a decreasing trend but was not clearly significant in the animal figure. Limitation: the model was acute Dex-induced atrophy in young mice, not natural aging sarcopenia.
**Park, 2020 [30]**	ICR mouse (male)	8 wk Body weight: 24.25 ± 1.06 g Experimentally induced atrophy	**Type:** Water extract of lotus leaf (LL; Nelumbo nucifera Gaertn) **Preparation:** Dried lotus leaf extracted with water at 95 °C for 4 h; filtered, concentrated, dextrin added, then dried **Model:** Dexamethasone-induced muscle atrophy **Vehicle:** 0.5% CMC solution **Route:** DEX in drinking water; LL oral administration **Dose:** DEX 0.01% *w*/*v* in drinking water; LL 100, 200, or 300 mg/kg/day **Frequency:** Daily for 4 weeks	Calf muscle Gastrocnemius muscle for molecular assays	**Calf muscle volume** DEX ↓ by ~30% vs. control (*p* < 0.01) LL 100, 200, 300 mg/kg ↑ vs. DEX (*p* < 0.01) **Calf muscle surface area** DEX ↓ by ~30% vs. control (*p* < 0.01) LL 100, 200, 300 mg/kg ↑ vs. DEX (*p* < 0.01) **Calf muscle density** DEX ↓ by ~40% vs. control (*p* < 0.01) LL recovered density by ~10%, 20%, and 30% at 100, 200, and 300 mg/kg, respectively LL 100/200 ↑ vs. DEX (*p* < 0.05); LL 300 ↑ vs. DEX (*p* < 0.01) **PCSA of calf muscle fibers** DEX ↓ vs. control (*p* < 0.01) LL 100, 200, 300 mg/kg ↑ vs. DEX (*p* < 0.01) **Body weight** DEX ↓ vs. control LL 300 mg/kg ↑ throughout 4 weeks LL 100 and 200 mg/kg ↑ at weeks 1, 3, and 4	**Muscle strength:** **Grip strength** DEX ↓ forelimb/hindlimb grip strength vs. control LL treatment restored grip strength starting from week 2 LL 100, 200, 300 mg/kg showed significant improvement at later time points **Physical performance:** **Moving distance / treadmill** DEX ↓ vs. control LL 100, 200, 300 mg/kg ↑ vs. DEX after weeks 1, 3, and 4 **Week 2:** only LL 100 mg/kg showed significant improvement; LL 200/300 showed ↑ trend	**Protein catabolic pathway** **Atrogin-1 mRNA:** DEX ↑ vs. control (*p* < 0.01); LL ↓ vs. DEX (*p* < 0.05 to *p* < 0.01) **MuRF1 mRNA:** DEX ↑ vs. control (*p* < 0.01); LL ↓ vs. DEX (*p* < 0.01) **FoxO/AMPK pathway** **FoxO1 mRNA:** no significant change **FoxO3a mRNA:** LL 200 and 300 mg/kg ↓ vs. DEX (*p* < 0.05 to *p* < 0.01) **p-AMPK:** DEX ↑ vs. control (*p* < 0.01); LL 200 and 300 mg/kg ↓ vs. DEX (*p* < 0.01) **Total AMPK:** unchanged **AKT/mTOR anabolic pathway** **p-mTOR:** DEX ↓ vs. control (*p* < 0.01); LL 300 mg/kg ↑ vs. DEX (*p* < 0.01) **Total mTOR:** unchanged **p-AKT:** LL 100, 200, 300 mg/kg ↑ vs. DEX (*p* < 0.05 to *p* < 0.01) **Total AKT:** unchanged **Active component validation** HPLC identified quercetin 3-O-beta-glucuronide (Q3G) as a major active component Q3G ↓ atrogin-1, MuRF1, FoxO3a, and p-AMPK in DEX-treated C2C12 cells Q3G ↑ p-AKT and p-mTOR in DEX-treated C2C12 cells	**ATP production** DEX ↓ **ATP production** LL recovered **ATP production** vs. DEX **Oxidative stress markers** **Direct ROS/MDA/SOD/GSH measurements in animal muscle:** N/A **Antioxidant-related evidence** LL contains polyphenol-rich components and Q3G, which is reported as an antioxidant-active compound Mechanistic data mainly support regulation of protein metabolism rather than direct in vivo antioxidant enzyme changes	LL administration for 4 weeks prevented Dex-induced muscle atrophy in mice. LL improved calf muscle volume, surface area, density, PCSA, body weight, moving distance, and grip strength. The anti-atrophy effect is mainly mediated by suppressing the AMPK/FoxO3a/atrogin-1/MuRF1 catabolic pathway and activating AKT/mTOR anabolic signaling. Q3G was identified as a major active phenolic component and reproduced the protein-metabolism regulatory effects in C2C12 cells. Direct in vivo oxidative stress markers were not measured, although ATP production was recovered and LL/Q3G have antioxidant-related properties. The study supports LL as a potential supplement or therapeutic candidate for muscle wasting, including sarcopenia.
**Saud Gany, 2026 [50]**	Sprague-Dawley rat (male)	Young: 3 mo Old: 21 mo Treatment duration: 3 mo Natural aging	**Type:** Tocotrienol-rich fraction (TRF; Golden TriTM E70) **Model:** Natural ageing rat model **Control:** Young and old control groups receiving palm olein vehicle **Vehicle:** Refined, bleached, and deodorized palm olein **Route:** Oral gavage **Dose:** 60 mg/kg body weight/day **Frequency:** Daily for 3 months	Gastrocnemius and soleus muscles Gastrocnemius for oxidative stress / metabolomics; soleus and gastrocnemius for histology	**Body composition / DEXA** TRF supplementation did not significantly restore muscle mass or overall body composition **Lean mass + BMC / total mass** N/A for direct anti-atrophy improvement; paper focused on ageing-associated body composition and redox/metabolic changes **Muscle histology** TRF improved histological features of aged muscle and reduced nuclear infiltration in old supplemented rats **Myofiber CSA** N/A	**Muscle strength:** Not assessed (grip strength was not included) **Physical performance:** Not assessed (endurance testing was not included) **Note** Authors noted that grip strength or endurance testing would complement biochemical and histological findings	**Protein metabolism signaling** N/A; Akt/mTOR/FoxO/MuRF1/Atrogin-1 were not assessed **Metabolomics / redox-inflammatory coupling** Ageing altered metabolite-biomarker correlations linking amino acid and carnitine metabolism with oxidative stress, inflammation, and DNA damage TRF weakened maladaptive correlations and strengthened associations between protective metabolites and antioxidant enzymes **DNA damage** TRF reduced DNA damage markers in older rats after prolonged intake	**Blood oxidative stress** TRF ↑ SOD and CAT activities TRF ↓ lipid peroxidation markers MDA and 4-HNE **Muscle oxidative stress** MDA and 4-HNE were measured in gastrocnemius muscle tissue **Inflammatory markers** CRP, IL-6, TNF-alpha, and PGF2-alpha were assessed TRF modulated inflammatory-marker/metabolite associations **Mitochondrial endpoints** **Direct mitochondrial respiration /** **ATP endpoints:** N/A	TRF acted mainly through redox, inflammatory, DNA-damage, and metabolomic network modulation rather than clear restoration of muscle mass. The study supports TRF as a nutritional strategy to improve antioxidant defence in ageing skeletal muscle. Muscle performance tests were not performed. Limitation: muscle mass/body composition was not significantly restored.
**Ramirez-Sanchez, 2026 [51]**	Sprague-Dawley rat (male)	23 mo old at treatment start Endpoint: 25 mo old Natural aging	**Type:** (+)-Epicatechin (+Epi; cacao flavanol) **Model:** Natural ageing / sarcopenia-related mitochondrial dysfunction **Control:** Vehicle-treated aged controls **Vehicle:** N/A / not clearly specified in main text **Route:** Oral administration **Dose:** 1 mg/kg/day **Frequency:** Daily for 8 weeks	Gastrocnemius skeletal muscle White blood cells for mitochondrial DNA / citrate synthase time-course endpoints	**Muscle mass** Main paper focused on mitochondrial and oxidative endpoints Authors state that in the same aged animals, +Epi previously increased muscle mass vs. controls **Myofiber CSA** N/A **Body weight** N/A	**Muscle strength:** **Grip strength** Main paper refers to prior results in the same animals showing ↑ front paw grip strength vs. controls **Physical performance:** **Treadmill time** Main paper refers to prior results in the same animals showing ↑ treadmill time vs. controls **Direct functional testing in this paper** N/A; current report analyzed mitochondrial / oxidative mechanisms using the same cohort	**NAD/SIRT1/PGC-1alpha axis** +Epi ↑ NAD+ (~80%) and ↑ NAD+/NADH ratio (>100%) vs. controls +Epi ↑ SIRT1 activation (~60%) and ↓ nuclear protein acetylation (~50%) +Epi ↓ acetylated PGC-1alpha (~70%) **Mitochondrial biogenesis signaling** +Epi ↑ TFAM and NRF1 mRNA (~50%) mtDNA copy number showed an increasing trend **Protein metabolism signaling** **Akt/mTOR/FoxO/MuRF1/Atrogin-1:** N/A in this main report	**Mitochondrial structure / function** +Epi ↑ SIRT3 (~35%) +Epi ↓ acetylated mitochondrial complexes I and V (~50%) +Epi ↑ complex I activity (~100%) +Epi ↑ complex I, complex V, mitofilin, and TFAM protein levels (~20–30%) +Epi ↑ citrate synthase activity (>200%) and ATP content (~90%) **Oxidative stress** Plasma protein carbonyls ↓ ~40% after 8 weeks Gastrocnemius protein carbonyls and MDA ↓ ~60% Total oxidized proteins in skeletal muscle ↓ ~50% **Antioxidant enzymes** SOD2 and catalase protein levels ↑ ~100% SOD2 activity ↑ ~60%; catalase activity ↑ ~100%	(+)-Epicatechin improved mitochondrial biogenesis/function and reduced oxidative stress in aged rat skeletal muscle. Benefits were linked to activation of the NAD/SIRT1/PGC-1alpha axis, SIRT3, mitochondrial complex activity, ATP, and antioxidant enzymes. Functional and muscle-mass benefits were cited from prior analyses of the same aged cohort rather than newly shown as primary endpoints in this report. Limitation: the main paper is mechanistic and does not provide full direct muscle mass/function tables.

**Note:** ↑, increase; ↓, decrease; ↔, no significant change or difference; →, change from the first value to the second value; ×, fold relative to the recommended intake or indicated reference level; NS, not significant; N/A, not applicable or not assessed. **Abbreviations: **Aox+, antioxidant-supplemented formulation; AOX, antioxidant-supplemented group; ATP, adenosine triphosphate; CTRL, control group; CSA, cross-sectional area; EDL, extensor digitorum longus; FSR, fractional synthesis rate; GM, gastrocnemius; GSH, reduced glutathione; Lowox, low-antioxidant diet group; MDA, malondialdehyde; mo, months; NAD+, nicotinamide adenine dinucleotide; NRF1, nuclear respiratory factor 1; OXPHOS, oxidative phosphorylation; PCA, principal component analysis; PGC-1α, peroxisome proliferator-activated receptor gamma coactivator 1-alpha; PLANT, plantaris; PROT, protein-supplemented group; ROS, reactive oxygen species; SOL, soleus; SIRT1, sirtuin 1; SOD, superoxide dismutase; TA, tibialis anterior; TAC, total antioxidant capacity; TBARS, thiobarbituric acid-reactive substances; TFAM, mitochondrial transcription factor A; TOTAL, combined antioxidant and protein-supplemented group; wk, weeks.

**Table 3:** **Basic Characteristics of the Included Preclinical Cellular Studies.**

Study	Cell Model	Intervention	Cellular Outcomes	Molecular Mechanisms	Validation Experiments	Other Outcomes	Key Findings
Mohamad Ishak et al., 2024 [27]	C2C12 myoblasts and myotubes; IEC6 intestinal epithelial cells (ancillary non-muscle model)	**Compound:** PQQ (pyrroloquinoline quinone) **Senescence model:** D-galactose (D-gal, 20 g/L) **Treatment:** Co-treatment with PQQ and D-gal **Concentration:** C2C12, 25, 50, and 100 nM; IEC6, 0.1–50 μM (proliferation) and 50 μM (barrier function) **Duration:** 24–72 h	**Cell viability (C2C12):** D-gal ↓ (*p* < 0.001); PQQ (25–100 nM) ↔ vs. D-gal **Cell proliferation (IEC6):** D-gal ↓; PQQ (50 μM) ↑ vs. D-gal (*p* < 0.001) **Senescence:** D-gal ↑ SA-β-gal-positive cells; PQQ ↓ (*p* < 0.05) **ROS levels:** D-gal ↑ 2.6-fold; PQQ (50, 100 nM) ↓ to control levels (*p* < 0.001) **Mitochondrial number:** D-gal (72 h) ↓; PQQ (50, 100 nM, 72 h) ↑ (*p* < 0.01) **Differentiation (fusion index):** D-gal ↓ (~10%); PQQ (100 nM) ↑ to ~40% (*p* < 0.001) **Barrier function (IEC6):** D-gal ↑ FITC-dextran permeability; PQQ (50 μM) ↓ (*p* < 0.05)	**Mitochondrial biogenesis-related markers:** PQQ prevented D-gal-induced decreases in PGC-1α and Tfam mRNA expression (*p* < 0.05). **Mitophagy-related features:** D-gal (24 h) increased mitophagy-related features; PQQ at 72 h was accompanied by findings interpreted as greater clearance of damaged mitochondria. **NAD^+^ metabolism:** D-gal decreased total NADH/NAD^+^ and NAD^+^ levels; PQQ (100 nM) prevented these decreases (*p* < 0.05).	**Evidence level:** Associative/inferred. **Direct pathway validation:** None; conclusions were based on marker changes and mitochondrial-related measurements.	**Ancillary non-muscle evidence (IEC6):** TEER decreased with D-gal and increased with PQQ (50 μM); FITC-dextran permeability increased with D-gal and decreased with PQQ (50 μM).	PQQ (25–100 nM) did not reverse the D-gal-induced reduction in C2C12 cell proliferation, but attenuated cellular senescence, reduced ROS levels to approximately control levels at 50–100 nM, and increased mitochondrial number and selected mitochondrial-related endpoints. PQQ enhanced myotube differentiation, with the fusion index increasing from ~10% to ~40% at 100 nM. In ancillary IEC6 experiments, PQQ (50 μM) increased cell proliferation and reduced monolayer permeability. These effects were accompanied by changes in PGC-1α/TFAM-related mitochondrial biogenesis markers, mitophagy-related features, and NAD^+^ metabolism; pathway dependence was not directly tested.
Choi et al., 2020 [61]	C2C12 myoblasts	**Compound:** Platycodin D (PD) **Oxidative-stress model:** H_2_O_2_ (1 mM) **Treatment:** PD pretreatment for 1 h followed by co-treatment with H_2_O_2_ **Concentration:** 10–40 μM (cytotoxicity assay); 30 μM (protective effects) **Duration:** 1 h pretreatment + 24 h co-treatment; 1 h for ROS measurement	**Cell viability:** H_2_O_2_ decreased viability to ~60% of control; PD (30 μM) increased viability versus H_2_O_2_ alone (*p* < 0.001). **ROS levels:** H_2_O_2_ increased intracellular ROS 9.7-fold; PD (30 μM) reduced this increase (*p* < 0.001), and NAC completely blocked ROS accumulation. **Mitochondrial ROS:** H_2_O_2_ increased MitoSOX-positive cells; PD (30 μM) reduced this increase. **Mitochondrial membrane potential (ΔΨm):** H_2_O_2_ decreased ΔΨm; PD (30 μM) attenuated the decrease. **Apoptosis:** H_2_O_2_ increased nuclear condensation/fragmentation and annexin V-positive cells; PD reduced these changes (*p* < 0.001). **DNA damage:** H_2_O_2_ increased comet-tail formation; PD reduced this increase. **Caspase-3 activity:** H_2_O_2_ increased activity; PD reduced this increase (*p* < 0.001).	**Mitochondrial apoptosis-related changes:** PD attenuated H_2_O_2_-associated cytochrome c release, Bax/Bcl-2 imbalance, caspase-3 activation, and PARP cleavage. **Mitophagy-related markers:** H_2_O_2_ increased Parkin and NIX/BNIP3L expression; PD reduced these changes. **Nrf2/HO-1 signaling:** PD + H_2_O_2_ increased Nrf2, p-Nrf2, and HO-1 and reduced Keap1; PD alone also increased Nrf2, p-Nrf2, and HO-1.	**Evidence level:** Direct experimental validation at the tested cellular endpoints. **Nrf2 siRNA:** Knockdown reduced PD-induced Nrf2 and HO-1 expression and reversed the PD-associated decrease in apoptosis and increase in cell viability (*p* < 0.01). **Positive control (NAC, 10 mM):** Completely blocked H_2_O_2_-induced ROS accumulation and cytotoxicity.	**Caspase-3 activity:** DEVDase assay **Cytochrome c release:** Mitochondrial/cytosolic fractionation **Bax/Bcl-2 ratio:** Western blot **DNA damage:** Comet assay **Mitochondrial membrane potential:** JC-1 staining **Mitochondrial ROS:** MitoSOX staining	Platycodin D (30 μM) attenuated H_2_O_2_-induced cytotoxicity, intracellular and mitochondrial ROS accumulation, DNA damage, and mitochondria-related apoptotic changes in C2C12 myoblasts. PD treatment preserved mitochondrial membrane potential limited cytochrome c release, reduced the Bax/Bcl-2 ratio, and decreased caspase-3 activation and PARP cleavage. PD treatment was accompanied by increased Nrf2, p-Nrf2, and HO-1 and decreased Keap1. Nrf2 knockdown reversed the PD-associated anti-apoptotic and cell-viability effects, supporting a direct role of Nrf2/HO-1 signaling at the tested cytoprotective endpoints.
He et al., 2025 [29]	C2C12 myoblasts and myotubes	**Compound:** Ferulic acid (FA) **Atrophy model:** Dexamethasone (Dex, 20 μM) **Treatment:** Co-treatment with FA and Dex **Concentration:** 10, 50, 200, and 500 μg/mL (proliferation); 10, 50, and 200 μg/mL (differentiation) **Duration:** 24–72 h	**Cell viability:** Dex reduced viability to 50–70% of control (*p* < 0.001); FA (50 and 200 μg/mL) increased viability versus Dex (*p* < 0.05 and *p* < 0.01). **Myotube diameter:** Dex reduced diameter; FA (50 and 200 μg/mL) increased diameter versus Dex (*p* < 0.01 and *p* < 0.001 at 72 h). **Inflammatory factors:** Dex increased TNF-α and IL-6; FA (50 and 200 μg/mL) reduced these increases (*p* < 0.01 and *p* < 0.001). **ROS levels:** Dex increased ROS; FA (200 μg/mL) reduced this increase (*p* < 0.01).	**ACOX1 protein expression:** Dex increased ACOX1; FA reduced this increase (*p* < 0.05). **ACOX1 enzyme activity:** Dex increased activity; FA reduced this increase (*p* < 0.01). **NRF2/HO-1-related markers:** FA did not significantly change HO-1, NQO1, or NRF2 nuclear translocation.	**Evidence level:** Mechanistic support without demonstration of pathway necessity. **ACOX1 inhibitor (TA, 1 μg/mL):** Reduced ACOX1 activity, ROS, and MuRF1 expression and increased myotube diameter versus Dex (*p* < 0.01). **Iron chelator (DFOM, 100 μM):** Reduced ROS but did not reverse FA-associated protection of myotube differentiation; DFOM increased NRF2 nuclear translocation and NQO1 expression, whereas FA did not. **Direct pathway validation:** TA phenocopied selected FA-associated effects, but ACOX1 was not shown to be required for the response to FA.	N/A	FA (50–200 μg/mL) attenuated Dex-associated reductions in C2C12 cell viability and myotube diameter and reduced TNF-α and IL-6 levels. FA (200 μg/mL) reduced Dex-associated ROS accumulation and lowered ACOX1 expression and activity. Pharmacological ACOX1 inhibition produced similar reductions in ROS and atrophy-related endpoints, supporting possible involvement of ACOX1. The study did not establish that ACOX1 was required for the effects of FA; FA also did not increase the assessed NRF2/HO-1-related markers.
Dong et al., 2022 [59]	C2C12 myoblasts	**Compound:** Ginsenoside Rb1 **Oxidative-stress model:** H_2_O_2_ (1 mM) **Treatment:** Rb1 pretreatment for 6 h followed by co-treatment with H_2_O_2_ **Concentration:** 20 and 40 μM (protective effects); 0–100 μM (cytotoxicity assay) **Duration:** 6 h pretreatment + 24 h co-treatment	**Cell viability:** H_2_O_2_ reduced viability to ~60% of control; Rb1 (20 and 40 μM) increased viability (*p* < 0.05). **Colony formation:** H_2_O_2_ reduced colony size and number; Rb1 increased both endpoints (*p* < 0.05). **Cell death and DNA damage:** Rb1 reduced H_2_O_2_-associated PI-positive cells and γ-H2AX (*p* < 0.05). **Apoptosis:** H_2_O_2_ increased apoptotic cells to ~40%; Rb1 reduced them to ~20–30% (*p* < 0.05). **Apoptosis-related proteins:** Rb1 reduced H_2_O_2_-associated cleaved caspase-3/9, Bax, and the Bax/Bcl-2 ratio and increased Bcl-2 (*p* < 0.05). **ROS levels:** Rb1 reduced H_2_O_2_-associated intracellular and mitochondrial ROS (*p* < 0.05).	**Mitochondrial membrane potential:** Rb1 attenuated the H_2_O_2_-associated decrease in the JC-1 red/green ratio (*p* < 0.05). **Cytochrome c release:** Rb1 attenuated H_2_O_2_-associated cytosolic translocation (*p* < 0.05). **ATP content:** Rb1 attenuated the H_2_O_2_-associated decrease (*p* < 0.05). **Mitochondrial biogenesis-related markers:** Rb1 increased mtDNA copy number and Tom20, PGC-1α, NRF1, and TFEB expression versus H_2_O_2_ alone (*p* < 0.05). **Metabolic gene expression:** Rb1 increased the assessed lipid- and glucose-metabolism genes versus H_2_O_2_ alone (*p* < 0.05). **Mitochondrial ultrastructure:** Rb1 reduced the frequency of abnormal mitochondria (*p* < 0.05).	**Evidence level:** Associative/inferred. **Direct pathway validation:** None; molecular docking and changes in NF-κB-related markers suggested possible pathway involvement, but no pathway-specific knockdown or inhibitor experiment was performed.	N/A	Ginsenoside Rb1 (20–40 μM) attenuated H_2_O_2_-associated cytotoxicity, reduced cell death and DNA damage (γ-H2AX), and increased colony formation in C2C12 myoblasts. Rb1 treatment was accompanied by lower cleaved caspase-3/9 and Bax, higher Bcl-2, and a lower Bax/Bcl-2 ratio. Rb1 reduced intracellular and mitochondrial ROS and was accompanied by higher mitochondrial membrane potential, lower cytochrome c release, and increased ATP content. Treatment was accompanied by increased mtDNA copy number and higher Tom20, PGC-1α, NRF1, and TFEB expression, together with improved mitochondrial ultrastructure and changes in lipid- and glucose-metabolism genes. Molecular docking and changes in NF-κB-related markers were consistent with possible involvement of NF-κB signaling; pathway dependence was not experimentally validated.
Kaminski et al., 2023 [64]	C2C12 myoblasts	**Compound:** Polyphenol-rich plant extract (PRPE) **Composition:** 25 g polyphenols/100 g extract (flavonoids: quercetin 3.4 g/100 g and glycosylated quercetin 5.1 g/100 g; hydroxycinnamic acids: chlorogenic acid 0.5 g/100 g and chicoric acid 5.1 g/100 g; phenylpropanoid caffeic acid glycosides 5.0 g/100 g) **Oxidative-stress model:** AAPH (5 mM) **Treatment:** Co-treatment with PRPE and AAPH **Concentration:** 10 and 50 μg/mL **Duration:** 24–48 h	**Cell morphology (phase contrast microscopy):** AAPH (5 mM, 24 h) induced morphological changes (cell shrinkage, detachment). PRPE (50 μg/mL) co-treatment prevented these changes. **Cell viability (trypan blue exclusion):** AAPH (5 mM, 24 h) ↓ viable cells (*p* < 0.05). PRPE (50 μg/mL) ↑ viable cells vs. AAPH alone (*p* < 0.05). PRPE (10 μg/mL) showed no significant effect. **Intracellular ROS (DHE staining, flow cytometry):** AAPH (5 mM, 24 h) ↑ DHE fluorescence (*p* < 0.05). PRPE (50 μg/mL, 48 h) ↓ DHE fluorescence vs. AAPH alone (*p* < 0.05). PRPE (10 μg/mL) showed no significant effect. **Gene expression (RT-qPCR):** **Prdx1:** AAPH (5 mM, 24 h) ↑ vs. control (*p* < 0.05); PRPE (50 μg/mL) ↓ vs. AAPH alone (*p* < 0.05). **PGC-1α:** AAPH ↑ vs. control (*p* < 0.05); PRPE (50 μg/mL) ↓ vs. AAPH alone (*p* < 0.05). **Nrf2:** AAPH showed a non-significant increase; PRPE showed a non-significant decrease. **Gpx, Sod1, Sod2, Ucp2, Ucp3:** no significant changes.	**Antioxidant-related findings:** PRPE reduced AAPH-induced intracellular ROS (DHE flow cytometry) and reduced AAPH-associated Prdx1 and PGC-1α gene-expression changes (*p* < 0.05).	**Evidence level:** Associative/inferred. **KRL hemolysis assay:** PRPE (10 and 20 μg/mL) increased the 50% hemolysis time from 76 ± 4 min with AAPH alone to 112 ± 6 and 151 ± 8 min, respectively. **Cell-free DHE assay:** PRPE (50 μg/mL) reduced AAPH-associated DHE fluorescence (*p* < 0.05); 10 μg/mL had no significant effect. **Direct pathway validation:** None; the study did not assess pathway dependence by siRNA, inhibition, or comparable loss-of-function experiments.	N/A	PRPE (50 μg/mL) attenuated AAPH-associated cytotoxicity, preserved cell morphology, and increased viable C2C12 cell counts. PRPE (50 μg/mL) reduced AAPH-associated intracellular ROS at 48 h and showed direct ROS-scavenging activity in a cell-free DHE assay. PRPE (10 and 20 μg/mL) increased the half-hemolysis time in the KRL assay. PRPE reduced AAPH-associated changes in Prdx1 and PGC-1α expression alongside lower intracellular ROS; no specific signaling pathway was directly validated. No significant cellular effects were observed at 10 μg/mL, indicating concentration-dependent activity under the tested conditions.
Ghzaiel et al., 2022 [65]	C2C12 myoblasts	**Compound:** Milk thistle seed oil (MTSO) and α-tocopherol (α-toco) **Toxic agent:** 7β-hydroxycholesterol (7β-OHC, 50 μM) **Treatment:** Co-treatment with MTSO or α-toco and 7β-OHC **Concentration:** MTSO, 100 μg/mL; α-toco, 400 μM **Duration:** 24 h	**Cell viability:** 7β-OHC reduced viability to ~50% of control; MTSO and α-tocopherol increased viability (*p* < 0.05). **Cell adhesion:** 7β-OHC reduced adherent cells; MTSO and α-tocopherol attenuated this reduction (*p* < 0.05). **Mitochondrial membrane potential:** 7β-OHC increased cells with depolarized mitochondria (~40%); MTSO and α-tocopherol reduced this increase (*p* < 0.05). **ROS levels:** 7β-OHC increased DHE-positive cells (~60%); MTSO and α-tocopherol reduced this increase (*p* < 0.05). **Lipid droplets:** 7β-OHC produced Oil Red O-positive cells (~10%); MTSO and α-tocopherol did not induce lipid droplets. **Peroxisomal mass and ultrastructure:** MTSO and α-tocopherol attenuated 7β-OHC-associated reductions in ABCD3 and abnormal peroxisomal/mitochondrial morphology. **VLCFA accumulation:** MTSO and α-tocopherol reduced 7β-OHC-associated C22:0, C24:0, C24:1n-9, C26:0, and C26:1n-9 accumulation (*p* < 0.05).	**Peroxisomal β-oxidation (Western blot):** 7β-OHC ↓ ACOX1 (75, 50, 25 kDa isoforms) and MFP2 (*p* < 0.05). MTSO and α-toco ↑ vs. 7β-OHC alone (*p* < 0.05). **Peroxisomal biogenesis (RT-qPCR):** 7β-OHC ↓ Abcd3 mRNA (*p* < 0.05) and Pex5 mRNA (*p* < 0.05). MTSO and α-toco ↑ vs. 7β-OHC alone (*p* < 0.05). 7β-OHC ↑ Pex13 and Pex14 mRNA (*p* < 0.05); MTSO and α-toco attenuated these increases. **Cellular accumulation of 7β-OHC (GC-MS):** 7β-OHC accumulated in cells (~4000 ng/million cells). MTSO ↓ accumulation by ~20%; α-toco ↓ by ~57% (*p* < 0.05).	**Evidence level:** Associative/inferred. **Direct pathway validation:** None; pathway-specific siRNA or inhibitor experiments were not performed. ACOX1-knockdown findings cited from previous work were not counted as direct validation in this review.	**7β-OHC accumulation:** 7β-OHC (50 μM, 24 h) accumulated to 3973 ng/million cells; MTSO reduced accumulation to 3196 ng/million cells; α-toco reduced to 1699 ng/million cells (*p* < 0.05). **Positive control:** Oleic acid (100 μM) induced lipid droplet formation (positive control for ORO staining), while MTSO and α-toco did not.	MTSO (100 μg/mL) and α-tocopherol (400 μM) attenuated 7β-OHC-associated reductions in cell viability, adhesion, and mitochondrial membrane potential and reduced ROS accumulation in C2C12 myoblasts. 7β-OHC exposure was accompanied by reduced peroxisomal mass, altered peroxisomal ultrastructure, lower ACOX1 and MFP2 expression, and accumulation of very-long-chain fatty acids. MTSO and α-tocopherol attenuated these peroxisomal alterations, increased ACOX1 and MFP2 expression, reduced VLCFA accumulation, and lowered cellular 7β-OHC content. These findings describe cytoprotective and peroxisome-related changes under the tested cellular conditions; pathway dependence and relevance to clinical sarcopenia were not directly established.
Fang et al., 2025 [67]	C2C12 myoblasts	**Compound:** Puerarin **Treatment:** Puerarin added to proliferation or differentiation medium **Concentration:** 0, 5, 10, 20, 40, and 100 μM (proliferation); 0, 5, 10, 20, and 40 μM (migration and differentiation) **Duration:** 24 h (proliferation and migration); 4 days (differentiation)	**Cell proliferation:** Puerarin (5–40 μM) produced no significant change; 100 μM reduced proliferation (*p* < 0.05). **Migration (scratch assay):** Puerarin (10, 20, and 40 μM) increased migration to 62.82%, 66.85%, and 65.13% versus 48.19% in control (*p* < 0.05 and *p* < 0.01). **Migration (transwell assay):** Puerarin increased migrated cell numbers (*p* < 0.05 and *p* < 0.01). **Differentiation:** Puerarin increased myotube formation and fusion index, with the largest fusion-index increase at 20 μM (*p* < 0.01). **Differentiation-related proteins:** Puerarin (20 μM) increased MHC, MyoD, and MyoG (*p* < 0.05 and *p* < 0.01).	**FAK signaling (migration):** Puerarin (20 μM) ↑ p-FAK/FAK ratio vs. control (*p* < 0.05). FAK inhibitor PF-573228 (10 μM) reversed puerarin-induced ↑ in p-FAK/FAK ratio (*p* < 0.01). **PI3K/AKT signaling (differentiation):** Puerarin (20 μM) ↑ p-PI3K/PI3K and p-AKT/AKT ratios vs. control (*p* < 0.05, *p* < 0.01). PI3K inhibitor LY294002 (10 μM) reversed puerarin-induced ↑ in p-PI3K/PI3K and p-AKT/AKT ratios (*p* < 0.05, *p* < 0.01).	**Evidence level:** Direct experimental validation at the tested cellular endpoints. **FAK inhibitor (PF-573228, 10 μM):** Abolished the puerarin-associated increase in migration without affecting basal migration (*p* < 0.05, *p* < 0.01). **PI3K inhibitor (LY294002, 10 μM):** Abolished the puerarin-associated increases in fusion index and MHC, MyoD, and MyoG expression without affecting basal differentiation (*p* < 0.01).	**Ancillary in vivo regeneration evidence (not counted as an independent aging-related animal dataset):** In BaCl_2_-injured TA muscle, puerarin (100 mg/kg/day orally for 5 days) increased newly formed myofiber area and MyHC expression versus injury control (*p* < 0.01).	Puerarin (10–40 μM) increased C2C12 myoblast migration without increasing proliferation; migration was greatest at 20 μM. PF-573228 abolished the puerarin-associated increase in migration, supporting direct involvement of FAK signaling at this endpoint. Puerarin increased myotube formation, fusion index, and MHC, MyoD, and MyoG expression. LY294002 abolished the puerarin-associated differentiation effects, supporting direct involvement of PI3K/AKT signaling at these endpoints. Ancillary BaCl_2_-injury experiments showed increased regenerating myofiber area and MyHC expression; these data were retained as supportive evidence only.
Lee et al., 2025 [63]	C2C12 myoblasts and myotubes	**Compound:** Riboflavin (Ribo) **Senescence model:** D-galactose (D-gal, 2 g/L) **Treatment:** Co-treatment with riboflavin and D-gal **Concentration:** 10 and 20 μM **Duration:** 48–72 h (viability); 5 days (differentiation); 24 h for circadian-rhythm analysis after dexamethasone synchronization	**Cell viability (MTT, 48 h):** D-gal ↓ (*p* < 0.001); Ribo (10, 20 μM) ↔ vs. D-gal. **Cell viability (CCK8, 72 h):** D-gal ↓; Ribo (20 μM) ↑ vs. D-gal (*p* < 0.01). **Differentiation (H&E staining):** D-gal ↓ myotube formation (28.5% vs. 95.6% in control, *p* < 0.0001); Ribo (20 μM) ↑ to 64.3% (*p* < 0.05 vs. D-gal). D-gal ↓ myotube width (6.8 μm vs. 31.2 μm in control, *p* < 0.0001); Ribo (20 μM) ↑ to 18.45 μm (*p* < 0.05 vs. D-gal). **Senescence (SA-β-gal staining):** D-gal ↑ SA-β-gal-positive cells (76.3% vs. 8.7% in control, *p* < 0.0001); Ribo (20 μM) ↓ to 29.4% (*p* < 0.0001 vs. D-gal). **ROS levels (DCF-DA):** D-gal ↑ (*p* < 0.0001); Ribo (20 μM) ↓ vs. D-gal (*p* < 0.05). **MDA levels:** D-gal ↑ (*p* < 0.0001); Ribo (20 μM) ↓ vs. D-gal (*p* < 0.0001). **Antioxidant enzyme activities (SOD, CAT, GPx):** D-gal ↓ SOD, CAT, GPx (all *p* < 0.0001); Ribo (20 μM) ↑ CAT (*p* < 0.01) and GPx (*p* < 0.01) vs. D-gal; SOD showed a trend toward improvement (*p* = 0.06). **Mitochondrial content (MitoTracker):** D-gal ↓; Ribo (20 μM) ↑ vs. D-gal. **ATP levels:** D-gal ↓ (~25%, *p* < 0.0001); Ribo (20 μM) ↑ vs. D-gal (*p* < 0.0001). **Mitochondrial membrane potential (JC-1 red/green ratio):** D-gal ↓ (*p* < 0.0001); Ribo (20 μM) ↑ vs. D-gal (*p* < 0.01).	**Differentiation-related gene oscillation:** D-gal reduced MyoD, myogenin, and Mrf4 oscillatory amplitude; riboflavin restored these oscillations. **Core clock-gene oscillation:** D-gal reduced Bmal1, Per2, Cry1, and Nr1d1 amplitude; riboflavin restored amplitude. Rora changed minimally, whereas riboflavin attenuated the D-gal-associated antiphase Rorc pattern. **OXPHOS-related proteins:** D-gal reduced ATP5A, MTCO1, and SDHB; riboflavin increased their expression and restored ATP5A diurnal oscillation.	**Evidence level:** Associative/inferred. **Direct pathway validation:** None; circadian and mitochondrial interpretations were based on gene/protein expression and functional measurements without siRNA or inhibitor testing.	N/A	Riboflavin (20 μM) attenuated D-galactose-associated impairment of myotube formation and width and reduced SA-β-gal-positive cells. Treatment was accompanied by lower ROS and MDA levels and higher CAT and GPx activities. Riboflavin increased mitochondrial content, ATP production, mitochondrial membrane potential, and selected OXPHOS protein levels. Treatment was associated with restoration of oscillations in differentiation-related and core clock genes disrupted by D-galactose. These concurrent changes were consistent with possible involvement of circadian and mitochondrial processes; pathway dependence and effects on clinical age-related muscle decline were not directly tested.
Raiteri et al., 2021 [68]	C2C12 myotubes (differentiated)	**Compounds:** VD3 (cholecalciferol), 25VD (calcidiol), and 1,25VD (calcitriol) **Treatment:** Treatment in serum-free medium **Concentration:** 100 nM for each metabolite **Duration:** 24 h	**Mitochondrial membrane potential (JC-1):** 1,25VD ↓ red/green ratio (*p* < 0.01). 25VD ↔ vs. control. VD3 ↑ red/green ratio (*p* < 0.05). **Mitochondrial respiration (intact cells, Oroboros):** 1,25VD ↔ vs. control in routine, LEAK, and ET. 25VD ↔ vs. control. VD3 ↑ routine (*p* < 0.05) and ET (*p* < 0.05). **Mitochondrial respiration (permeabilized cells, complex I OXPHOS):** 1,25VD ↓ (*p* < 0.05). 25VD and VD3 ↔ vs. control. **Mitochondrial content (mtDNA):** 1,25VD ↔ vs. control. 25VD and VD3 ↑ (*p* < 0.05, *p* < 0.01). **Mitochondrial morphology (MiNA):** No changes in footprint or branches with any treatment. **Mitophagy (LC3B-MitoTracker colocalization, LC3B-VDAC co-IP):** 1,25VD ↑ LC3B puncta (*p* < 0.05) and LC3B-VDAC interaction. 1,25VD + CLQ did not further increase LC3B-VDAC interaction, indicating blocked mitophagic flux. **ROS production (CellROX):** 1,25VD ↑ (*p* < 0.01). 25VD and VD3 ↓ (*p* < 0.01). **Mitochondrial ROS (H_2_O_2_ flux, Amplex Red, permeabilized cells):** 1,25VD ↔ vs. control (only slight, non-significant increase). **Myotube diameter:** 1,25VD ↓ (*p* < 0.01). NAC (5 mM) co-treatment prevented 1,25VD-induced reduction (*p* < 0.01). mitoTEMPO (10 μM) partially prevented reduction (*p* < 0.05 vs. 1,25VD alone). **Atrogene expression (MuRF1, Atrogin-1):** 1,25VD ↑ (*p* < 0.01). NAC co-treatment prevented 1,25VD-induced upregulation (*p* < 0.01). mitoTEMPO co-treatment did not prevent upregulation (*p* > 0.05 vs. 1,25VD alone). **Mitochondrial depolarization (JC-1):** 1,25VD ↓ red/green ratio (*p* < 0.01). NAC co-treatment prevented depolarization (*p* < 0.01). mitoTEMPO co-treatment did not prevent depolarization (*p* > 0.05 vs. 1,25VD alone).	**PKC activation:** Calcitriol increased p-PKC substrates within 5–20 min (*p* < 0.05). **PKC inhibition:** GÖ6850 prevented calcitriol-associated ROS production, reduced myotube diameter, atrogene upregulation, and mitochondrial depolarization. **JNK inhibition:** SP600125 prevented the calcitriol-associated reduction in myotube diameter (*p* < 0.01).	**Evidence level:** Direct experimental validation of the calcitriol-induced atrophic mechanism at the tested cellular endpoints. **NAC (5 mM):** Prevented calcitriol-associated ROS production, reduced myotube diameter, atrogene upregulation, and mitochondrial depolarization (*p* < 0.01). **MitoTEMPO (10 μM):** Partially prevented the reduction in myotube diameter but did not prevent atrogene upregulation or mitochondrial depolarization. **PKC inhibitor (GÖ6850, 1 μM):** Prevented calcitriol-associated ROS production, myotube atrophy, atrogene upregulation, and mitochondrial depolarization (all *p* < 0.01). **JNK inhibitor (SP600125, 1 μM):** Prevented calcitriol-associated reduction in myotube diameter (*p* < 0.01). **Chloroquine (10 μM):** The absence of further LC3B-VDAC accumulation was consistent with blocked mitophagic flux.	N/A	Calcitriol (1,25VD) induced C2C12 myotube atrophy, mitochondrial depolarization, and increased ROS, whereas VD3 and 25VD reduced basal ROS and increased selected mitochondrial endpoints. The response pattern was consistent with a predominantly non-mitochondrial ROS source because mitochondrial H_2_O_2_ flux changed minimally and MitoTEMPO provided only partial endpoint rescue. PKC inhibition prevented calcitriol-associated ROS production, myotube atrophy, atrogene upregulation, and mitochondrial depolarization. JNK inhibition also prevented the reduction in myotube diameter, supporting a PKC–ROS–JNK-dependent mechanism for calcitriol-induced cellular atrophy. Calcitriol-associated LC3B-VDAC accumulation did not increase further with chloroquine, consistent with blocked mitophagic flux. These experiments support an atrophic mechanism in C2C12 myotubes but do not demonstrate a protective antioxidant mechanism in aged muscle.
Khor et al., 2017 [66]	Human primary myoblasts (young, presenescent, senescent)	**Compounds:** Tocotrienol-rich fraction (TRF), α-tocopherol (ATF), and N-acetyl-cysteine (NAC) **Treatment:** Single or combination treatment **Concentration:** TRF, 50 μg/mL; ATF, 50 μg/mL; NAC, 1.0 mg/mL; TRF + NAC, 25 μg/mL + 1.0 mg/mL; ATF + NAC, 25 μg/mL + 1.0 mg/mL **Duration:** 24 h	**Cell morphology (anti-Desmin staining):** Senescent control showed fewer spindle-shaped cells; TRF, ATF, NAC, TRF + NAC, and ATF + NAC improved morphology. **Senescence (SA-β-gal staining):** Senescent control ↑ SA-β-gal-positive cells (*p* < 0.05 vs. young). TRF, ATF, NAC, TRF + NAC, and ATF + NAC ↓ SA-β-gal-positive cells vs. senescent control (*p* < 0.05). **ROS levels (carboxy-H_2_DCFDA and DHE, flow cytometry):** Presenescent and senescent controls ↑ ROS (*p* < 0.05 vs. young). TRF and ATF ↓ carboxy-H_2_DCFDA in presenescent and senescent (*p* < 0.05). TRF + NAC ↓ carboxy-H_2_DCFDA in senescent (*p* < 0.05). ATF ↓ DHE in senescent (*p* < 0.05). **Lipid peroxidation (C11-BODIPY, oxidized/reduced ratio):** Presenescent and senescent controls ↑ ratio (*p* < 0.05 vs. young). TRF and ATF ↓ ratio in presenescent and senescent (*p* < 0.05). NAC ↑ ratio in presenescent (*p* < 0.05). TRF + NAC and ATF + NAC ↓ ratio in senescent (*p* < 0.05). **Antioxidant enzyme activities:** **Sod:** Senescent control ↔ vs. young; TRF ↑ in presenescent and senescent (*p* < 0.05). **Cat:** Senescent control ↓ vs. young (*p* < 0.05); TRF ↑ in senescent (*p* < 0.05); ATF ↑ in presenescent (*p* < 0.05). **Gpx:** Senescent control ↑ vs. young (*p* < 0.05); TRF and ATF ↓ in senescent (*p* < 0.05). **GSH/GSSG ratio:** No significant change with aging. NAC ↑ ratio in young, presenescent, and senescent (*p* < 0.05). TRF ↓ ratio in young and presenescent (*p* < 0.05). **Cell viability (MTS assay):** Senescent control ↓ vs. young (*p* < 0.05). TRF, ATF, NAC, TRF + NAC, and ATF + NAC ↑ vs. respective controls (*p* < 0.05). **Apoptosis (Annexin V/PI flow cytometry):** Senescent control ↑ early and late apoptosis (*p* < 0.05 vs. young). TRF, ATF, NAC, TRF + NAC, and ATF + NAC ↓ early apoptosis in senescent (*p* < 0.05). TRF, TRF + NAC, and ATF + NAC ↓ late apoptosis in senescent (*p* < 0.05).	**Antioxidant enzyme mRNA expression (RT-qPCR):** **SOD1:** No significant changes. **SOD2:** Presenescent control ↑ vs. young (*p* < 0.05); TRF ↑ in young, presenescent, and senescent (*p* < 0.05); TRF + NAC ↑ in young (*p* < 0.05). **CAT:** Presenescent control ↑ vs. young (*p* < 0.05); TRF and ATF ↑ in senescent (*p* < 0.05); TRF + NAC ↑ in young (*p* < 0.05). **GPX1:** TRF ↑ in young, presenescent, and senescent (*p* < 0.05). **Vitamin E uptake (HPLC):** TRF-treated cells contained all five vitamin E isomers (ATF, ATT, BTT, GTT, DTT). ATF-treated cells contained primarily ATF. TRF + NAC-treated cells contained high levels of all five isomers; ATF + NAC-treated cells contained primarily ATF.	**Evidence level:** Associative/inferred for the proposed antioxidant-defense mechanisms. **NAC comparator (1.0 mg/mL):** Increased the GSH/GSSG ratio but did not significantly reduce ROS or lipid peroxidation or alter SOD2, CAT, or GPX1 mRNA in senescent myoblasts. **Combination treatments:** TRF + NAC increased SOD activity and reduced apoptosis more than NAC alone; ATF + NAC increased CAT activity in senescent myoblasts (*p* < 0.05). **Direct pathway validation:** None; the study compared treatments but did not test pathway dependence by knockdown or inhibition.	N/A	Replicative senescence in human primary myoblasts was associated with increased ROS, lipid peroxidation, and altered antioxidant-enzyme activities. TRF (50 μg/mL) reduced ROS and lipid peroxidation and was accompanied by changes in SOD and CAT activities and SOD2, CAT, and GPX1 mRNA expression. ATF (50 μg/mL) reduced ROS and lipid peroxidation and altered CAT and GPx-related endpoints, but showed narrower changes than TRF under the tested conditions. NAC increased the GSH/GSSG ratio and reduced senescence markers but did not significantly reduce ROS or lipid peroxidation. TRF + NAC showed greater changes in selected antioxidant-enzyme and apoptosis endpoints than NAC alone. TRF showed broader antioxidant-enzyme activity and expression changes than α-tocopherol; the mechanism underlying this difference was not directly validated.
Choi et al., 2021 [62]	C2C12 myoblasts	**Compound:** Trans-cinnamaldehyde (tCA) **Oxidative-stress model:** H_2_O_2_ (1 mM) **Treatment:** tCA pretreatment for 1 h followed by co-treatment with H_2_O_2_ **Concentration:** 5, 10, and 20 μM (protective effects); 0–50 μM (cytotoxicity assay) **Duration:** 1 h pretreatment + 24 h co-treatment; 1 h for ROS measurement	**Cell viability (MTT assay):** H_2_O_2_ ↓ to ~60% of control (*p* < 0.001). tCA (10, 20 μM) ↑ vs. H_2_O_2_ alone (*p* < 0.01, *p* < 0.001). tCA (5 μM) showed no significant effect. **DNA damage (comet assay):** H_2_O_2_ ↑ comet tail formation; tCA (20 μM) ↓ vs. H_2_O_2_ alone. **DNA damage markers (Western blot):** H_2_O_2_ ↑ p-γH2AX and p-ATM; tCA (20 μM) ↓ vs. H_2_O_2_ alone. **Apoptosis (DAPI staining):** H_2_O_2_ ↑ nuclear condensation and fragmentation; tCA (20 μM) ↓ vs. H_2_O_2_ alone. **DNA fragmentation (agarose gel):** H_2_O_2_ ↑ DNA laddering; tCA (20 μM) ↓ vs. H_2_O_2_ alone. **Apoptosis (Annexin V/PI flow cytometry):** H_2_O_2_ ↑ apoptotic cells (~5-fold vs. control, *p* < 0.001); tCA (20 μM) ↓ vs. H_2_O_2_ alone (*p* < 0.001). **Mitochondrial membrane potential (JC-1 flow cytometry):** H_2_O_2_ ↓ red/green ratio (↑ % of cells with JC-1 monomers, *p* < 0.001); tCA (20 μM) prevented ↓ vs. H_2_O_2_ alone (*p* < 0.001). **Cytochrome c release (Western blot, mitochondrial/cytosolic fractions):** H_2_O_2_ ↑ cytochrome c in cytosol, ↓ in mitochondria; tCA (20 μM) prevented release. **Bax and Bcl-2 expression (Western blot):** H_2_O_2_ ↑ Bax, ↓ Bcl-2; tCA (20 μM) reversed these changes. **Bax/Bcl-2 ratio:** H_2_O_2_ ↑; tCA (20 μM) ↓ vs. H_2_O_2_ alone. **Caspase-3 activation (Western blot, colorimetric assay):** H_2_O_2_ ↑ cleaved caspase-3 and caspase-3 activity (*p* < 0.001); tCA (20 μM) ↓ vs. H_2_O_2_ alone (*p* < 0.001). **PARP cleavage (Western blot):** H_2_O_2_ ↑ cleaved PARP; tCA (20 μM) ↓ vs. H_2_O_2_ alone. **ROS levels (DCF-DA flow cytometry and fluorescence microscopy):** H_2_O_2_ ↑ ROS (~4-fold vs. control, *p* < 0.001); tCA (20 μM) ↓ vs. H_2_O_2_ alone (*p* < 0.001).	**Mitochondrial apoptosis-related changes:** tCA attenuated H_2_O_2_-associated mitochondrial depolarization, cytochrome c release, Bax/Bcl-2 imbalance, caspase-3 activation, and PARP cleavage. **ROS levels:** tCA treatment was accompanied by lower H_2_O_2_-induced intracellular ROS accumulation measured by DCF-DA.	**Evidence level:** Associative/inferred for pathway-level interpretation. **Direct pathway validation:** None; siRNA or pathway-inhibitor experiments were not performed. **Positive control (NAC, 10 mM):** Completely blocked H_2_O_2_-associated cytotoxicity (*p* < 0.001).	N/A	Trans-cinnamaldehyde (10–20 μM) attenuated H_2_O_2_-associated cytotoxicity in C2C12 myoblasts, with the largest effect at 20 μM. tCA treatment was accompanied by less DNA damage, nuclear condensation, DNA fragmentation, and Annexin V positivity. Treatment also attenuated mitochondrial depolarization, cytochrome c release, Bax/Bcl-2 imbalance, caspase-3 activation, and PARP cleavage. tCA reduced H_2_O_2_-associated intracellular ROS accumulation. These concurrent changes were consistent with attenuation of mitochondrial apoptosis-related processes; pathway dependence was not directly tested.
Sciandra et al., 2023 [60]	C2C12 myoblasts and myotubes	**Compound:** Verbascoside (V) **Oxidative-stress model:** H_2_O_2_ (0.5–1 mM) **Treatment:** Verbascoside pretreatment followed by co-treatment with H_2_O_2_ **Concentration:** 150 μM (primary); 19–150 μM (dose-response) **Duration:** 24 h pretreatment; H_2_O_2_ exposure for 1 h (myoblasts) or 2.5 h (myotubes)	**Cell viability (MTT):** H_2_O_2_ ↓ myoblast viability to 69% (*p* < 0.001); V (150 μM) restored to 88% (*p* < 0.001 vs. H_2_O_2_). H_2_O_2_ ↓ myotube viability to 16% (*p* < 0.001); V (75, 150 μM) restored to 38% and 44% (*p* < 0.01, *p* < 0.001 vs. H_2_O_2_). **ROS levels (DCF-DA):** H_2_O_2_ ↑ ROS in myoblasts and myotubes (*p* < 0.001). V (150 μM) ↓ ROS vs. H_2_O_2_ alone in myoblasts (*p* < 0.01). V (75, 150 μM) ↓ ROS vs. H_2_O_2_ alone in myotubes (*p* < 0.05, *p* < 0.01). V (150 μM) also ↓ endogenous ROS in myoblasts and myotubes (*p* < 0.05). **Mitochondrial respiration (high-resolution respirometry, intact cells):** **Basal OCR:** V ↔ vs. control in both myoblasts and myotubes. **Maximal OCR:** V ↑ by 27% in myoblasts (*p* < 0.01) and 13% in myotubes (*p* < 0.05) vs. control. **Proton leak and residual OCR:** unchanged. **Spare respiratory capacity (SRC, maximal OCR/basal OCR):** V ↑ SRC by 51% in myoblasts (*p* < 0.01) and 13% in myotubes (*p* < 0.05) vs. control. **RCR and CE:** unchanged. **Mitochondrial respiration under oxidative stress:** H_2_O_2_ ↓ basal OCR and maximal OCR in myoblasts and myotubes. V did not restore basal OCR but ↑ maximal OCR by 77% in myoblasts (*p* < 0.01) and 25% in myotubes (*p* < 0.05) vs. H_2_O_2_ alone. V ↑ SRC by 34% in myoblasts (*p* < 0.05) and 61% in myotubes (*p* < 0.05) vs. H_2_O_2_ alone. V restored RCR in myotubes by 66% (*p* < 0.05).	**Nrf2/HO-1-related signaling:** Verbascoside + H_2_O_2_ increased Nrf2 protein, the p-Nrf2/Nrf2 ratio in myoblasts, Nrf2 nuclear localization, and HO-1 mRNA under the tested conditions. **PGC-1α expression:** Verbascoside increased PGC-1α mRNA in myoblasts but not in myotubes.	**Evidence level:** Associative/inferred. **Direct pathway validation:** None; Nrf2/HO-1 involvement was inferred from expression and localization changes without siRNA or inhibitor validation.	N/A	Verbascoside (150 μM) increased viability and reduced ROS in C2C12 myoblasts and myotubes exposed to H_2_O_2_. Verbascoside increased maximal oxygen consumption and spare respiratory capacity under basal conditions. Under oxidative stress, treatment partially increased maximal OCR and spare respiratory capacity and increased the respiratory control ratio in myotubes. These effects occurred concurrently with increased Nrf2 expression, Nrf2 phosphorylation/nuclear localization, and HO-1 expression, consistent with Nrf2/HO-1-related signaling. Pathway dependence was not directly validated, and PGC-1α expression changed in myoblasts but not myotubes.
Park et al., 2020 [30]	C2C12 myoblasts (differentiated)	**Compound:** Quercetin 3-O-β-glucuronide (Q3G) **Atrophy model:** Dexamethasone (DEX, 100 μM) **Treatment:** Co-treatment with Q3G and DEX **Concentration:** 6.25, 12.5, and 20 μM **Duration:** 24 h	**Cell viability (MTT assay):** Q3G (3.15–25 μM) alone ↔ vs. control (*p* > 0.05). DEX (100 μM) alone ↔ vs. control (*p* > 0.05) (no cytotoxicity). **Atrogene mRNA expression (RT-qPCR):** **Atrogin-1:** DEX ↑ (*p* < 0.01 vs. control); Q3G (6.25, 12.5, 20 μM) ↓ vs. DEX alone (*p* < 0.05, *p* < 0.01). **MuRF1:** DEX ↑ (*p* < 0.01 vs. control); Q3G (6.25, 12.5, 20 μM) ↓ vs. DEX alone (*p* < 0.05, *p* < 0.01). **FoxO3a:** DEX ↑ (*p* < 0.01 vs. control); Q3G (6.25, 12.5, 20 μM) ↓ vs. DEX alone (*p* < 0.05, *p* < 0.01). **Western blot (protein expression):** p-AKT/AKT: DEX ↓ (*p* < 0.01 vs. control); Q3G (6.25, 12.5, 20 μM) ↑ vs. DEX alone (*p* < 0.05, *p* < 0.01). p-mTOR/mTOR: DEX ↓ (*p* < 0.01 vs. control); Q3G (6.25, 12.5, 20 μM) ↑ vs. DEX alone (*p* < 0.05, *p* < 0.01). p-AMPK/AMPK: DEX ↑ (*p* < 0.01 vs. control); Q3G (6.25, 12.5, 20 μM) ↓ vs. DEX alone (*p* < 0.05, *p* < 0.01).	**Proteolysis-related markers:** Q3G reduced DEX-associated increases in atrogin-1, MuRF1, and FoxO3a mRNA and AMPK phosphorylation. **Protein-synthesis-related markers:** Q3G increased DEX-suppressed AKT and mTOR phosphorylation.	**Evidence level:** Associative/inferred. **Direct pathway validation:** None; AKT/mTOR- and AMPK/FoxO-related interpretations were not tested by siRNA or inhibitor experiments.	**Ancillary in vivo evidence (not counted as an independent aging-related animal dataset):** In DEX-treated mice, lotus leaf extract (100, 200, or 300 mg/kg for 4 weeks) increased calf muscle volume, surface area, density, grip strength, running distance, and body weight and produced changes in atrogin-1, MuRF1, FoxO3a, AMPK, AKT, and mTOR-related markers. HPLC identified Q3G as a major extract component.	Q3G (6.25–20 μM) did not affect C2C12 viability but reduced DEX-associated increases in atrogin-1, MuRF1, and FoxO3a mRNA. Treatment was accompanied by increased AKT and mTOR phosphorylation and decreased AMPK phosphorylation. These findings were consistent with modulation of protein synthesis- and degradation-related signaling, but pathway dependence was not directly tested.
Kanazawa et al., 2026 [31]	C2C12 myoblasts; A549 and HepG2 cells (ancillary non-muscle models)	**Compound/formulation:** Edaravone-loaded human serum albumin nanoparticles (HSA-NPs) **Oxidative-stress model:** H_2_O_2_-treated C2C12 cells (0.5 mM; 4 h for ROS and 12 h for lipid peroxidation) **Treatment:** Free edaravone, HSA + edaravone, or edaravone-loaded HSA-NPs; Cy5-HSA-NPs for uptake studies **Concentration:** Edaravone, 0.17 mg/mL for 4 h; Cy5-HSA-NPs, 0.1 mg HSA/mL	**SPARC and uptake:** H_2_O_2_ ↑ SPARC; SPARC level correlated with HSA-NP uptake (R^2^ = 0.4388, *p* = 0.0052). **Intracellular edaravone:** HSA-NPs ↑ delivery by ~5-fold vs. free edaravone or HSA + edaravone (*p* < 0.05). **Oxidative damage:** HSA-NPs ↓ H_2_O_2_-induced ROS to near-control levels and ↓ lipid peroxidation (*p* < 0.05).	**SPARC-guided uptake:** Thiol-rich HSA-NPs were internalized through SPARC-dependent thiol/disulfide interactions. **Intracellular release:** HSA-NPs entered lysosomes; acidic lysosomal processing promoted edaravone release into the cytosol.	**Evidence level:** Direct experimental validation of the nanoparticle uptake/delivery mechanism. **SPARC siRNA (20 nM):** Reduced HSA-NP uptake (*p* < 0.01). **Uptake/processing inhibitors:** NEM, EIPA, chlorpromazine, and chloroquine reduced uptake or ROS-related activity. **Interpretive boundary:** These experiments support SPARC-mediated uptake and lysosomal processing, not proof of a downstream muscle-protective pathway.	**Localization:** Cy5-HSA-NPs co-localized with lysosomes in C2C12 cells.	Edaravone-loaded HSA-NPs increased intracellular edaravone delivery and reduced H_2_O_2_-associated ROS and lipid peroxidation in C2C12 cells. SPARC siRNA and uptake-inhibitor experiments supported a direct role of SPARC-mediated uptake in HSA-NP delivery; this validation was specific to the delivery/uptake endpoint.
Jia et al., 2026 [33]	C2C12 myoblasts and differentiated myotubes	**Compound:** Ginsenoside Ro (GRo); eight ginsenosides were initially screened **Senescence/atrophy model:** D-galactose (D-gal, 20 mg/mL) **Treatment:** GRo with D-gal; screening used 10 μM of each ginsenoside **Concentration:** GRo, 5, 10, or 20 μM **Duration:** 48 h	**Cell viability/senescence:** GRo showed the strongest protection in the screen and reduced the SA-β-gal-positive area to 6.94%. **Myotube diameter:** Control 15.00 ± 2.40 μm; D-gal 8.68 ± 2.26 μm; GRo 10.97 ± 1.96 μm. **Mitochondrial function:** GRo ↓ ROS and restored mitochondrial membrane potential, MitoTracker fluorescence, and ATP in a dose-dependent manner.	**Redox/mitochondrial protection:** GRo attenuated D-gal-induced oxidative stress and mitochondrial dysfunction.	**Evidence level:** Associative/inferred. **Direct pathway validation:** None; comparative screening identified GRo as the strongest candidate among eight ginsenosides, but no pathway-specific siRNA or inhibitor was used.	N/A	GRo reduced D-gal-induced senescence and partially restored myotube diameter. Protection was associated with lower ROS and improved mitochondrial function.
Lim et al., 2026 [32]	C2C12 myoblasts/myotubes; human muscle satellite cells (MSCs)	**Compound:** Amentoflavone (AMF) **Atrophy models:** Dexamethasone (Dex, 10 μM) or recombinant myostatin (MSTN, 100 ng/mL) during differentiation **Concentration:** C2C12, 0.01–10 nM; human MSCs, 1–1000 nM **Duration:** Differentiation assessed for up to 6 days	**Myogenic differentiation:** AMF ↑ CK activity, myotube formation/fusion, and MYOD/MYOG/MYH expression in C2C12 cells and human MSCs. **Atrophy:** AMF reversed Dex- or MSTN-induced loss of myogenic markers and ↓ Atrogin-1 and MuRF1. **Oxidative stress:** AMF ↓ intracellular ROS in both cell models.	**MSTN-SMAD-related signaling:** AMF treatment was accompanied by lower MSTN expression and SMAD2/3 phosphorylation. **Antioxidant-related markers:** AMF treatment was accompanied by higher NRF2 and SOD2 expression.	**Evidence level:** Target-engagement and mechanistic support without demonstration of pathway necessity. **CETSA:** AMF (10 nM) increased MSTN thermal stability, supporting intracellular target engagement. **Recombinant MSTN challenge:** AMF dose-dependently attenuated MSTN-associated differentiation impairment. **Direct pathway validation:** Pathway necessity was not established by loss-of-function experiments.	**Human-cell confirmation:** Core effects were reproduced in primary human MSCs.	AMF increased myogenic differentiation and attenuated Dex- and MSTN-associated atrophy-related endpoints in mouse and human muscle cells. CETSA and recombinant MSTN challenge provided target-engagement and mechanistic support for involvement of MSTN-SMAD signaling; pathway necessity was not established by loss-of-function experiments.
Chang et al., 2024 [26]	Differentiated C2C12 myotubes	**Compound:** Oligonol® (Olg, 50 μg/mL) **Nutrient/atrophy model:** BCAAs (0.8 mM) and palmitic acid (PA, 250 μM) **Validation treatments:** LAT1 siRNA and LAT1 inhibitor JPH203 (1 μM) **BCAA stimulation:** 16 h	**LAT1 knockdown:** LAT1 mRNA decreased from 1.003 ± 0.058 to 0.157 ± 0.007; LAT2 was unchanged. **Protein-synthesis signaling:** LAT1 knockdown blunted BCAA-induced mTOR/p70S6K phosphorylation and increased BCAT2 ubiquitination. **Oligonol + BCAA:** PA reduced LAT1 and mTOR/p70S6K signaling; Olg restored LAT1, and Olg + BCAA restored signaling more than BCAA alone.	**LAT1-dependent BCAA signaling:** Olg enhanced BCAA-stimulated mTOR/p70S6K signaling by restoring LAT1-mediated BCAA transport. **BCAT2 stability:** Reduced LAT1/BCAA availability increased BCAT2 ubiquitination.	**Evidence level:** Direct experimental validation at the tested cellular signaling endpoint. **LAT1 siRNA:** Reduced BCAA-induced mTOR/p70S6K activation and increased BCAT2 ubiquitination. **LAT1 inhibitor (JPH203, 1 μM):** Reversed the Olg + BCAA effect on mTOR/p70S6K signaling.	N/A	LAT1 was required for BCAA-induced mTOR/p70S6K signaling in C2C12 myotubes. Olg restored LAT1 under PA stress and enhanced the anabolic response to BCAAs.
Chen et al., 2024 [28]	C2C12 myotubes differentiated for 6 days	**Compound:** Polygonatum sibiricum polysaccharide (PSP; purity ≥98%) **Senescence model:** D-galactose (D-gal, 40 g/L) for 48 h **Treatment:** PSP pretreatment before D-gal exposure **Concentration:** 50 or 100 mg/L **Duration:** PSP for 24 h before D-gal exposure	**Senescence:** PSP ↓ SA-β-gal, P16, and P53; ↑ SIRT1 and SIRT3. **Oxidative stress/mitochondria:** PSP ↓ ROS; ↑ T-SOD, CAT, ATP, mitochondrial membrane potential, and respiration. **MAM and Ca^2^^+^:** PSP ↓ ER-mitochondrial contacts and reduced intracellular and mitochondrial Ca^2^^+^ accumulation.	**MAM-related calcium-transfer markers:** PSP treatment was accompanied by lower IP3R, GRP75, and VDAC1 in the MAM fraction and lower mitochondrial MCU expression.	**Evidence level:** Associative/inferred. **Direct pathway validation:** None; fractionation, imaging, and calcium measurements supported a MAM-related interpretation, but no pathway-specific siRNA or inhibitor was used.	**Positive control:** Sulforaphane showed similar protective trends in supplementary experiments.	PSP reduced D-gal-associated senescence-related endpoints and increased selected mitochondrial functional measures in C2C12 myotubes. Treatment was accompanied by lower MAM-related protein expression, fewer ER–mitochondrial contacts, and lower mitochondrial Ca^2^^+^ accumulation, consistent with possible modulation of MAM-mediated calcium transfer; pathway dependence was not directly validated.

Cellular stress models were used for mechanistic interrogation, not as direct equivalents of clinical sarcopenia. Ancillary non-muscle and non-aging experiments were supportive only and were not counted as independent datasets. **Note:** ↑, increase; ↓, decrease; ↔, no significant change or difference; ×, fold relative to the indicated reference level; ±, values are presented as mean ± standard deviation (SD) or as reported in the original study; NS, not significant; N/A, not applicable or not assessed. Cellular stress models were used primarily for mechanistic interrogation and were not considered direct equivalents of clinical sarcopenia. Ancillary non-muscle and non-aging experiments were supportive only and were not counted as independent datasets. **Abbreviations:** AAPH, 2,2′-azobis(2-amidinopropane) dihydrochloride; ABCD3, ATP-binding cassette subfamily D member 3; ACOX1, acyl-CoA oxidase 1; AKT, protein kinase B; AMF, amentoflavone; AMPK, adenosine monophosphate-activated protein kinase; ATF, α-tocopherol; ATP, adenosine triphosphate; BCAA, branched-chain amino acid; BCAT2, branched-chain amino acid transaminase 2; Bcl-2, B-cell lymphoma-2; CK, creatine kinase; D-gal, D-galactose; DCF-DA, 2′,7′-dichlorofluorescin diacetate; DEX, dexamethasone; DHE, dihydroethidium; EDL, extensor digitorum longus; ER, endoplasmic reticulum; FAK, focal adhesion kinase; GC-MS, gas chromatography–mass spectrometry; GM, gastrocnemius; GPx, glutathione peroxidase; GRo, ginsenoside Ro; GRP75, glucose-regulated protein 75; GSH/GSSG, reduced glutathione/oxidized glutathione ratio; H_2_O_2_, hydrogen peroxide; HPLC, high-performance liquid chromatography; HSA-NPs, human serum albumin nanoparticles; IEC6, rat intestinal epithelial cell line; IL-6, interleukin-6; IP3R, inositol 1,4,5-trisphosphate receptor; JC-1, 5,5′,6,6′-tetrachloro-1,1′,3,3′-tetraethylbenzimidazolylcarbocyanine iodide; KRL, Kit Radicaux Libres; LAT1, L-type amino acid transporter 1; LC3B, microtubule-associated protein 1 light chain 3 beta; MAM, mitochondria-associated membrane; MDA, malondialdehyde; MHC, myosin heavy chain; MitoTEMPO, mitochondria-targeted superoxide dismutase mimetic; MSCs, muscle satellite cells; MTSO, milk thistle seed oil; MuRF1, muscle RING-finger protein-1; NAC, N-acetyl-cysteine; NAD^+^, nicotinamide adenine dinucleotide; NF-κB, nuclear factor kappa B; NRF1, nuclear respiratory factor 1; Nrf2, nuclear factor erythroid 2-related factor 2; Olg, Oligonol®; OXPHOS, oxidative phosphorylation; PA, palmitic acid; PCA, principal component analysis; PGC-1α, peroxisome proliferator-activated receptor gamma coactivator 1-alpha; PI3K, phosphoinositide 3-kinase; PKC, protein kinase C; PQQ, pyrroloquinoline quinone; PRPE, polyphenol-rich plant extract; PSP, Polygonatum sibiricum polysaccharide; Q3G, quercetin 3-O-β-glucuronide; ROS, reactive oxygen species; RT-qPCR, reverse transcription quantitative polymerase chain reaction; SA-β-gal, senescence-associated β-galactosidase; SDHB, succinate dehydrogenase complex iron sulfur subunit B; siRNA, small interfering RNA; SIRT1, sirtuin 1; SIRT3, sirtuin 3; SOD, superoxide dismutase; TA, tibialis anterior; TAC, total antioxidant capacity; TBARS, thiobarbituric acid-reactive substances; TFAM, mitochondrial transcription factor A; TRF, tocotrienol-rich fraction; tCA, trans-cinnamaldehyde; VD3, cholecalciferol; 25VD, calcidiol; 1,25VD, calcitriol; VDAC1, voltage-dependent anion channel 1; VLCFA, very-long-chain fatty acids; ΔΨm, mitochondrial membrane potential.

## Data Availability

All data analyzed in this systematic review were derived from the published studies cited in the article. Extracted study characteristics and outcome data are summarized in the main tables, and methodological appraisal results are provided in the Supplementary Materials.

## Supplementary Materials

The following supporting information can be downloaded at https://www.mdpi.com/article/10.3390/antiox15091167/s1, Figure S1: Cochrane RoB 2 domain-level assessment for individual clinical trials (*n* = 8); Figure S2: Cochrane RoB 2 domain-level risk distribution (*n* = 8 RCTs); Figure S3: Modified SYRCLE risk-of-bias assessment for individual animal studies (*n* = 26); Figure S4: Modified SYRCLE risk-of-bias domain-level distribution (*n* = 26); Figure S5: Cellular study quality: criterion-level summary (*n* = 18); Figure S6: In vitro quality assessment: modified ToxRTool (*n* = 18); Table S1: Search strategy: detailed search strings for each database; Table S2: Primary Analytical Classification and Experimentally Supported Secondary Mechanistic Actions in the 36 Preclinical Publications Reporting Mechanistic Outcomes.
