# Supplementary material for: Antioxidant Interventions on Muscle Function and Mass in Aging-Related Muscle Decline: A Systematic Review of Preclinical and Clinical Evidence
Source: Antioxidants (Basel). 2026 Sep 15;15(9):1167. doi: 10.3390/antiox15091167 (PMC13603870; doi:10.3390/antiox15091167)

Supplementary Figure S1. Cochrane RoB 2 domain-level assessment for individual clinical trials (n = 8)

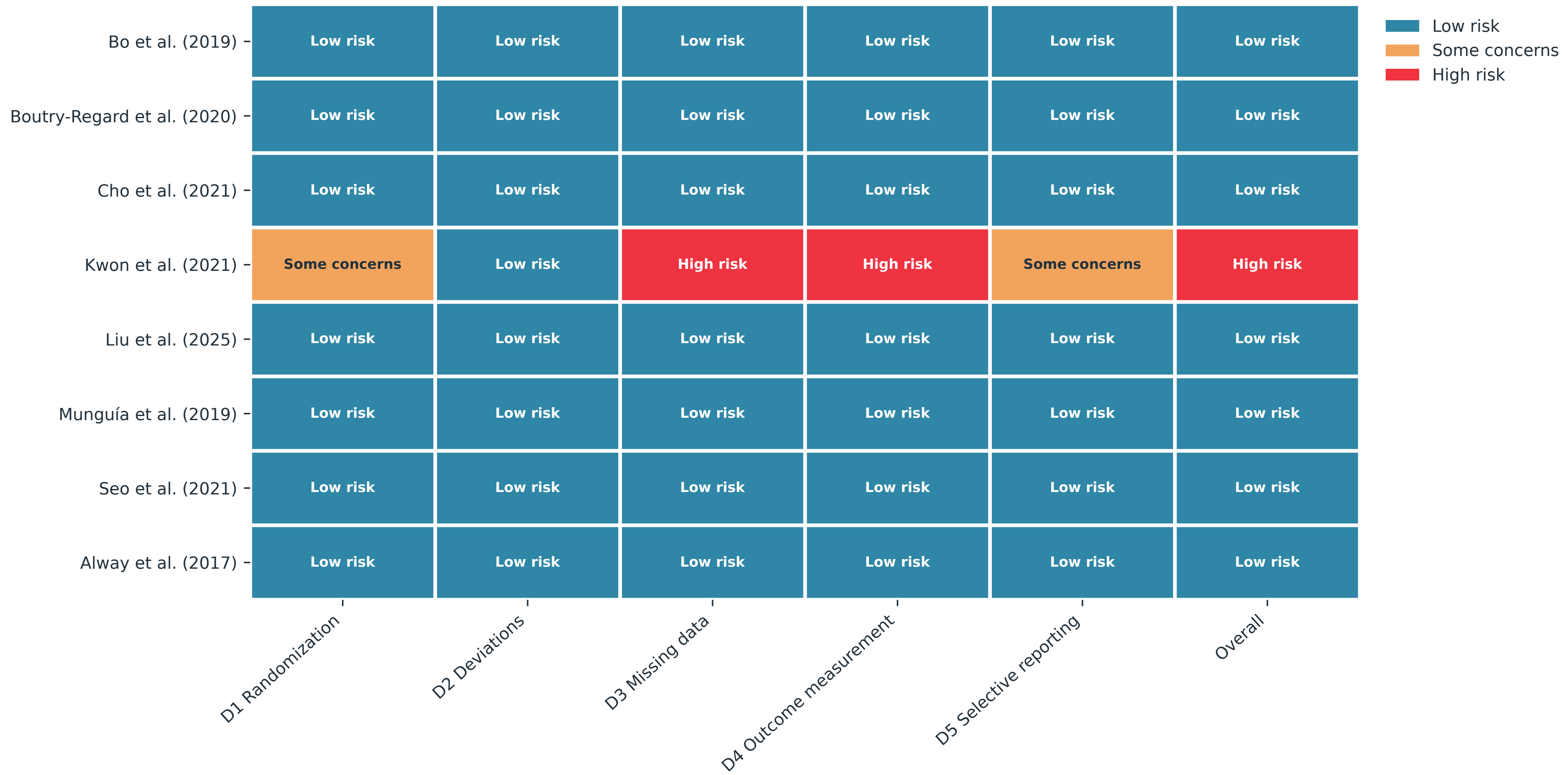

Supplement: Supplementary file 1 [file antioxidants-15-01167-s001.zip › Supplementary Figure S1. Cochrane RoB 2 domain-level assessment for individual clinical trials(4).pdf]
