# Supplementary material for: Antioxidant Interventions on Muscle Function and Mass in Aging-Related Muscle Decline: A Systematic Review of Preclinical and Clinical Evidence
Source: Antioxidants (Basel). 2026 Sep 15;15(9):1167. doi: 10.3390/antiox15091167 (PMC13603870; doi:10.3390/antiox15091167)
Supplement: Supplementary file 1 [file antioxidants-15-01167-s001.zip › Supplementary Figure S3. SYRCLE risk-of-bias assessment for individual animal studies_corrected(4).pdf]

Supplementary Figure S3. Modified SYRCLE risk-of-bias assessment for individual animal studies (n = 26)

Study	Sequence generation	Baseline characteristics	Housing / husbandry	Blinding	Random outcome assessment	Incomplete outcome data	Selective reporting	Other bias	Overall
Wang (2020)	Low	Unclear	Unclear	Unclear	Unclear	Some concerns	Low	Low	Some concerns
Vays (2014)	Low	Unclear	Unclear	Unclear	Unclear	Low	Low	Low	Unclear
Marzani (2008)	Unclear	Unclear	Unclear	Unclear	Unclear	Low	Low	Low	Unclear
Zainul Azlan (2020)	Unclear	Unclear	Unclear	Unclear	Unclear	Low	Low	Low	Unclear
Gatineau (2015)	Low	Unclear	Unclear	Unclear	Unclear	Low	Low	Low	Unclear
Mohamad Ishak (2024)	Some concerns	Low	Unclear	Unclear	Unclear	Low	Low	Some concerns	Some concerns
Munguia (2020)	Low	Low	Unclear	Unclear	Unclear	Low	Low	Low	Unclear
Annunziata (2020)	Unclear	Unclear	Unclear	Unclear	Unclear	Low	Low	Low	Unclear
Mosoni (2014)	Some concerns	Low	Unclear	Unclear	Unclear	Low	Low	Some concerns	Some concerns
van Dijk (2016)	Low	Low	Unclear	Unclear	Unclear	Low	Low	Low	Unclear
Sinha-Hikim (2013)	Unclear	Low	Unclear	Unclear	Unclear	Low	Low	Low	Unclear
Jackson (2011)	Low	Unclear	Unclear	Unclear	Unclear	Low	Low	Low	Unclear
Javadov (2015)	Low	Low	Unclear	Unclear	Unclear	Low	Low	Low	Unclear
Wang (2023)	Low	Unclear	Unclear	Unclear	Unclear	Low	Low	Low	Unclear
Chang (2024)	Unclear	Unclear	Unclear	Unclear	Unclear	Low	Low	Low	Unclear
Chen (2024)	Low	Unclear	Unclear	Low	Low	Low	Low	Low	Unclear
Toniolo (2018)	Low	Unclear	Unclear	Unclear	Low	Some concerns	Low	Low	Some concerns
Joseph (2013)	Low	Low	Unclear	Unclear	Unclear	Some concerns	Low	Low	Some concerns
Takisawa (2019)	Unclear	Unclear	Unclear	Unclear	Low	Low	Low	Low	Unclear
He (2025)	Low	Unclear	Unclear	Unclear	Low	Low	Low	Low	Unclear
Park (2020)	Low	Unclear	Unclear	Unclear	Unclear	Low	Low	Low	Unclear
Ramirez-Sanchez (2026)	Low	Unclear	Unclear	Unclear	Unclear	Low	Low	Low	Unclear
Kanazawa (2026)	Unclear	Unclear	Unclear	Unclear	Unclear	Low	Low	Low	Unclear
Saud Gany (2026)	Unclear	Low	Unclear	Unclear	Unclear	Low	Low	Low	Unclear
Lim (2026)	Unclear	Unclear	Unclear	Unclear	Unclear	Low	Low	Low	Unclear
Jia (2026)	Unclear	Unclear	Unclear	Unclear	Unclear	Low	Low	Low	Unclear

Low
Unclear
Some concerns
