# Supplementary material for: Antioxidant Interventions on Muscle Function and Mass in Aging-Related Muscle Decline: A Systematic Review of Preclinical and Clinical Evidence
Source: Antioxidants (Basel). 2026 Sep 15;15(9):1167. doi: 10.3390/antiox15091167 (PMC13603870; doi:10.3390/antiox15091167)
Supplement: Supplementary file 1 [file antioxidants-15-01167-s001.zip › Supplementary Figure S6. In vitro quality assessment – detailed criteria per study(4).pdf]

Supplementary Figure S6. In vitro quality assessment: modified ToxRTool (n = 18)

Study	Objective defined	Substance identity	Test system described	Study design reported	Concentration justified	Appropriate controls	Adequate replication	Blinding of examination	Outcome measures valid	Statistics appropriate
Choi (2020)	Y	PY	Y	Y	Y	Y	Y	N	Y	Y
Mohamad Ishak (2024)	Y	PY	Y	Y	Y	Y	PY	N	Y	Y
He (2025)	Y	Y	Y	Y	Y	Y	Y	N	Y	Y
Dong (2022)	Y	Y	Y	Y	Y	Y	Y	N	Y	Y
Kaminski (2023)	Y	Y	Y	Y	PY	Y	Y	N	Y	Y
Ghzaiel (2022)	Y	Y	Y	Y	Y	Y	Y	N	Y	Y
Fang (2025)	Y	N	Y	Y	Y	Y	Y	N	Y	Y
Lee (2025)	Y	PY	PY	Y	Y	Y	PY	N	Y	Y
Raiteri (2021)	Y	Y	Y	Y	PY	Y	Y	N	Y	Y
Khor (2017)	Y	Y	Y	Y	Y	Y	Y	N	Y	Y
Choi (2021)	Y	PY	Y	Y	Y	Y	Y	N	Y	Y
Sciandra (2023)	Y	PY	Y	Y	Y	Y	Y	N	Y	PY
Park (2020)	Y	Y	Y	Y	Y	Y	Y	N	Y	PY
Chang (2024)	Y	Y	Y	PY	PY	Y	Y	N	Y	PY
Chen (2024)	Y	Y	Y	Y	Y	Y	Y	N	Y	PY
Kanazawa (2026)	Y	PY	Y	Y	Y	Y	Y	N	Y	Y
Lim (2026)	Y	Y	Y	Y	Y	Y	Y	N	Y	Y
Jia (2026)	Y	PY	Y	Y	Y	Y	Y	N	Y	Y

Legend: Y, PY, N
