# Supplementary material for: Antioxidant Interventions on Muscle Function and Mass in Aging-Related Muscle Decline: A Systematic Review of Preclinical and Clinical Evidence
Source: Antioxidants (Basel). 2026 Sep 15;15(9):1167. doi: 10.3390/antiox15091167 (PMC13603870; doi:10.3390/antiox15091167)
Supplement: Supplementary file 1 [file antioxidants-15-01167-s001.zip › Supplementary Table S1.Search strategy(4).pdf]

Supplementary Table S1. Search strategy: detailed search strings for each database

Search date: 11 July 2026.

**PubMed**	Filters applied	• None
	Search strategy	("sarcopenia"[MeSH Terms] OR "sarcopenia"[Title/Abstract] OR "muscle wasting"[Title/Abstract] OR "muscle loss"[Title/Abstract]) AND ("aged"[MeSH Terms] OR "aging"[MeSH Terms] OR "elderly"[Title/Abstract] OR "aging"[Title/Abstract] OR "aged"[Title/Abstract]) AND ("antioxidants"[MeSH Terms] OR "antioxidant*"[Title/Abstract] OR "vitamin C"[Title/Abstract] OR "ascorbic acid"[Title/Abstract] OR "vitamin E"[Title/Abstract] OR "tocopherol*"[Title/Abstract] OR "polyphenol*"[Title/Abstract] OR "flavonoid*"[Title/Abstract])
**Embase**	Filters applied	• None
	Search strategy	((sarcopenia/exp OR sarcopenia:ti,ab OR 'muscle wasting':ti,ab OR 'muscle loss':ti,ab) AND (aged/exp OR aging/exp OR elderly:ti,ab OR aging:ti,ab OR aged:ti,ab) AND ('antioxidant agent'/exp OR antioxidant*:ti,ab OR 'vitamin c':ti,ab OR 'ascorbic acid':ti,ab OR 'vitamin e':ti,ab OR tocopherol*:ti,ab OR polyphenol*:ti,ab OR flavonoid*:ti,ab))
**Web of Science**	Filters applied	• None
	Search strategy	TS=((("sarcopenia" OR "muscle wasting" OR "muscle loss") AND ("elderly" OR "aging" OR "aged") AND ("antioxidants" OR "antioxidant*" OR "vitamin C" OR "ascorbic acid" OR "vitamin E" OR "tocopherol*" OR "polyphenol*" OR "flavonoid*")))
**Cochrane CENTRAL**	Filters applied	• None
	Search strategy	(sarcopenia OR "muscle wasting" OR "muscle loss") AND (elderly OR aging OR aged) AND (antioxidant* OR "vitamin C" OR "ascorbic acid" OR "vitamin E" OR tocopherol* OR polyphenol* OR flavonoid*)
