# Supplementary material for: Antioxidant Interventions on Muscle Function and Mass in Aging-Related Muscle Decline: A Systematic Review of Preclinical and Clinical Evidence
Source: Antioxidants (Basel). 2026 Sep 15;15(9):1167. doi: 10.3390/antiox15091167 (PMC13603870; doi:10.3390/antiox15091167)
Supplement: Supplementary file 1 [file antioxidants-15-01167-s001.zip › Supplementary Table S2. Mechanistic Overlap.pdf]

## Supplementary Table S2. Primary Analytical Classification and Experimentally Supported Secondary Mechanistic Actions in the 36 Preclinical Publications Reporting Mechanistic Outcomes

| Study | Intervention | Primary analytical category | Basis for primary assignment | Secondary mechanistic actions / overlapping categories | Supporting endpoints in the included study |
|---|---|---|---|---|---|
| **1. van Dijk et al., 2016** | Leucine-enriched whey plus vitamins A/E, selenium, and zinc | Vitamins and antioxidant micronutrients | Nutritional multicomponent formulation in which vitamins and trace elements were supplied to support antioxidant defense. | Glutathione/redox homeostasis; mitochondrial-dynamics regulation; muscle function and quality. | Muscle GSH and MDA; Mfn1/2, Dnm1l, Fis1, Mff, and Mief1/2; grip strength, fatigue resistance, and muscle quality. |
| **2. Marzani et al., 2008** | Rutin plus vitamins E/A, zinc, and selenium | Vitamins and antioxidant micronutrients | Vitamin–trace-element antioxidant mixture investigated as nutritional supplementation in aged muscle. | Leucine-sensitive protein synthesis; inflammatory modulation. | Leucine-stimulated muscle protein synthesis; S6K1 signaling/oxidation; circulating inflammatory markers. |
| **3. Gatineau et al., 2015** | Rutin plus vitamins E/A/D, selenium, and zinc | Vitamins and antioxidant micronutrients | Vitamin–trace-element antioxidant mixture used to counter diet-associated redox and metabolic impairment. | Glutathione/redox defense; insulin sensitivity; muscle protein synthesis. | Plasma antioxidant capacity; muscle GSH and SOD; insulin sensitivity; fractional muscle protein-synthesis rate. |
| **4. Mosoni et al., 2014** | Whey/chamomile formulation with vitamins E and D | Vitamins and antioxidant micronutrients | Multinutrient strategy containing vitamin antioxidants, analyzed primarily as nutritional antioxidant supplementation. | Inflammatory and redox modulation; muscle protein turnover. | Fibrinogen and α2-macroglobulin; GSH, SOD, total antioxidant capacity, protein carbonyls, and TBARS; protein synthesis and proteolysis. |
| **5. Takisawa et al., 2019** | Vitamin C depletion and repletion | Vitamins and antioxidant micronutrients | Essential antioxidant vitamin studied through dietary deficiency and subsequent repletion. | Ubiquitin–proteasome-related proteolysis and muscle atrophy. | Muscle weight and myofiber CSA; grip strength and endurance; MAFbx and MuRF1; recovery after vitamin C repletion. |
| **6. Saud Gany et al., 2026** | Tocotrienol-rich fraction | Vitamins and antioxidant micronutrients | Vitamin E isoform mixture administered to reinforce endogenous antioxidant defense in aged muscle. | Inflammatory modulation; DNA protection; metabolic-profile modulation. | SOD and CAT activities; MDA and 4-HNE; CRP, IL-6, and TNF-α; comet assay; muscle histology and metabolite–marker correlations. |
| **7. Lee et al., 2025** | Riboflavin | Vitamins and antioxidant micronutrients | Essential vitamin investigated as a nutritional redox cofactor and circadian modulator. | Circadian regulation; mitochondrial energetics; cellular senescence. | Clock-gene rhythmicity; ROS, MDA, CAT, and GPx; ATP, mitochondrial membrane potential, and ATP5A rhythms; senescence and myotube width. |
| **8. Raiteri et al., 2021** | 1,25(OH)$_2$ vitamin D$_3$ (calcitriol) | Vitamins and antioxidant micronutrients | Active vitamin D metabolite examined as a vitamin-derived regulator of myotube redox status. | PKC–ROS–JNK signaling; mitochondrial dysfunction; autophagic flux; atrophy signaling. | NAC, GÖ6850, and SP600125 interventions; ROS; myotube diameter and atrogenes; mitochondrial membrane potential; chloroquine-based flux assessment. |
| **9. Khor et al., 2017** | Tocotrienol-rich fraction/α-tocopherol, with NAC comparator | Vitamins and antioxidant micronutrients | Vitamin E isoforms were the principal interventions used to restore antioxidant defense in senescent human myoblasts. | Glutathione homeostasis; enzymatic antioxidant defense; lipid-peroxidation control. | ROS and lipid peroxidation; SOD2, CAT, and GPX1 expression/activity; GSH/GSSG; NAC comparator. |
| **10. Chang et al., 2024** | Oligonol® | Dietary polyphenols and plant-derived extracts | Oligomerized litchi polyphenol investigated as a plant-derived polyphenolic intervention. | LAT1-dependent BCAA transport; mTOR-mediated protein synthesis; BCAT2-dependent BCAA catabolism. | JPH203 and LAT1 siRNA; LAT1 transport; mTOR/p70S6K; BCAT2 expression and ubiquitination; myotube diameter. |
| **11. Chen et al., 2024** | Polygonatum sibiricum polysaccharide | Dietary polyphenols and plant-derived extracts | Plant-derived polysaccharide/extract classified by its natural origin and phytochemical identity. | Mitochondria-associated membrane regulation; $Ca^{2+}$ homeostasis; mitochondrial bioenergetics. | ER–mitochondria contacts; IP3R–GRP75–VDAC1 and MCU; cellular/mitochondrial $Ca^{2+}$; oxygen-consumption parameters. |
| **12. He et al., 2025** | Ferulic acid | Dietary polyphenols and plant-derived extracts | Plant-derived phenolic acid; the primary intervention identity was a dietary polyphenol. | Peroxisomal ACOX1 signaling; redox and inflammatory modulation; proteolysis. | ACOX1 expression/activity; ROS; inflammatory mediators and MuRF1; pharmacological ACOX1 inhibition with 10,12-tricosadiynoic acid (TA), which reproduced selected FA-associated effects. |
| **13. Park et al., 2020** | Lotus-leaf water extract / quercetin-3-O-β-glucuronide | Dietary polyphenols and plant-derived extracts | Plant extract and its principal flavonoid metabolite were investigated as phytochemical interventions. | Akt/mTOR protein synthesis; AMPK/FOXO3a and ubiquitin–proteasome signaling; cellular energetics. | p-Akt/p-mTOR; p-AMPK/p-FOXO3a; MAFbx and MuRF1; ATP content; myotube and muscle-fiber size. |
| **14. Lim et al., 2026** | Amentoflavone | Dietary polyphenols and plant-derived extracts | Plant-derived biflavonoid classified by phytochemical identity despite multiple downstream targets. | Myostatin–SMAD inhibition; Akt/mTOR anabolism; Nrf2/SOD2 redox response. | CETSA evidence of myostatin engagement; MSTN/SMAD2/3; Akt/mTOR; ROS, Nrf2, and SOD2; atrogenes, strength, and CSA. |
| **15. Jia et al., 2026** | Ginsenoside Ro | Dietary polyphenols and plant-derived extracts | Plant-derived ginsenoside investigated as a phytochemical intervention. | Mitochondrial protection; redox and inflammatory modulation; gut-microbiota/metabolic effects. | ROS, mitochondrial membrane potential/mass, and ATP; SOD, MDA, and GSH; IL-1β, IL-6, and TNF-α; 16S rDNA profiles. |

| Study | Intervention | Primary analytical category | Basis for primary assignment | Secondary mechanistic actions / overlapping categories | Supporting endpoints in the included study |
|---|---|---|---|---|---|
| **16. Wang, D. et al., 2020** | Apigenin | Dietary polyphenols and plant-derived extracts | Plant-derived flavone; phytochemical identity was used for the unique primary assignment. | Mitochondrial biogenesis and respiration; antioxidant defense; mitophagy and apoptosis. | PGC-1α, NRF1, TFAM, and mtDNA; respiration and ATP; SOD/GPx; BNIP3, p62, and LC3-II/I; apoptotic markers. |
| **17. Jackson et al., 2011** | Resveratrol | Dietary polyphenols and plant-derived extracts | Plant-derived stilbene investigated as a dietary polyphenol. | Endogenous antioxidant defense and oxidative-damage control. | MnSOD; hydrogen peroxide and lipid peroxidation; PGC-1α; muscle mass and function (oxidative stress improved without attenuation of sarcopenia). |
| **18. Toniolo et al., 2018** | Resveratrol | Dietary polyphenols and plant-derived extracts | Plant-derived stilbene investigated as dietary polyphenol supplementation. | Oxidative muscle phenotype; mitochondrial enzyme activity; excitation–contraction structure and fatigue resistance. | SDH activity; Tom20 and COX IV; MyHC/SERCA1; tubular aggregates; ex vivo fatigue resistance. |
| **19. Joseph et al., 2013** | Resveratrol (alone or with caloric restriction) | Dietary polyphenols and plant-derived extracts | Plant-derived stilbene used as a polyphenolic caloric-restriction mimetic. | SIRT1/PGC-1α-related mitochondrial protein regulation; apoptosis; fiber-type-specific remodeling. | SIRT1 and PGC-1α; Bax/Bcl-2; mitochondrial protein-expression panels by muscle type; mitochondrial respiration. |
| **20. Annunziata et al., 2020** | Taurisolo® grape polyphenols | Dietary polyphenols and plant-derived extracts | Grape-derived polyphenol formulation classified by phytochemical origin. | Endogenous antioxidant enzymes; Sirt1 signaling; inflammatory modulation. | CAT, GPx, and glutathione reductase; Sirt1; IL-6 and IL-10; oxidative-damage indices; rotarod performance. |
| **21. Zainul Azlan et al., 2020** | Chlorella vulgaris | Dietary polyphenols and plant-derived extracts | Whole-algal plant-derived supplement classified according to natural-source extract identity. | Oxidative-damage control; muscle integrity, mass, and function. | Urinary $F_2$-isoprostane; plasma CK-MM; muscle MDA plus HAE; muscle mass, grip strength, and locomotor outcomes. |
| **22. Wang, H.-H. et al., 2023** | Nobiletin | Dietary polyphenols and plant-derived extracts | Citrus-derived polymethoxylated flavone classified by phytochemical identity. | Akt/mTOR protein synthesis; FOXO3a–MAFbx/MuRF1 proteolysis; inflammatory modulation. | p-Akt/Akt, p-mTOR/mTOR, and p70S6K; p-FOXO3a/FOXO3a, MAFbx, and MuRF1; LBP, TNF-α, and IL-6; muscle CSA. |
| **23. Munguia et al., 2020** | Dark-chocolate flavonoids / (−)-epicatechin | Dietary polyphenols and plant-derived extracts | Cocoa-derived flavonoids classified by their dietary polyphenol identity. | Myostatin/follistatin balance; myogenic and atrophy signaling; muscle-damage modulation. | Follistatin and myostatin; MEF2A, FOXO1A, and MuRF1; mobility and muscle-damage measures. |
| **24. Ramirez-Sanchez et al., 2026** | (+)-Epicatechin | Dietary polyphenols and plant-derived extracts | Flavanol investigated as a dietary polyphenol despite prominent mitochondrial actions. | SIRT1/PGC-1α signaling; mitochondrial biogenesis and bioenergetics; endogenous antioxidant enzymes. | $NAD^+$/NADH, SIRT1, and PGC-1α deacetylation; NRF1/TFAM; ETC complexes I/V, mitofilin, citrate synthase, ATP, SOD2, and CAT. |
| **25. Ghzaiel et al., 2022** | Milk-thistle seed oil | Dietary polyphenols and plant-derived extracts | Plant-derived oil/extract was the principal phytochemical intervention. | Peroxisomal integrity and β-oxidation; mitochondrial protection; redox control. | ROS and mitochondrial membrane potential; ultrastructure; ABCD3, PEX5/13/14, ACOX1, and MFP2; very-long-chain fatty acids. |
| **26. Kaminski et al., 2023** | Polyphenol-rich plant extract | Dietary polyphenols and plant-derived extracts | Plant extract standardized by its polyphenol-rich composition. | Direct radical-scavenging/cytoprotection; antioxidant-response gene modulation. | Cell-free antioxidant capacity; intracellular ROS and viability; PRDX1; Nrf2- and PGC-1-related transcripts (without demonstrated Nrf2 activation). |
| **27. Fang et al., 2025** | Puerarin | Dietary polyphenols and plant-derived extracts | Plant-derived isoflavone classified by phytochemical identity. | FAK and PI3K/Akt signaling; myoblast migration and myogenic differentiation. | PF-573228 and LY294002 inhibition; migration and fusion indices; MyoD, MyoG, and MyHC. |
| **28. Mohamad Ishak et al., 2024** | Pyrroloquinoline quinone (PQQ) | Mitochondria-targeted or mitochondria-modulating antioxidants | Compound was investigated primarily for mitochondrial biogenesis, quality control, and bioenergetic effects. | Mitophagy/fusion; redox and inflammatory modulation; intestinal-barrier effects. | Mitochondrial number, PGC-1α/TFAM, $NAD^+$, ATP, and mitophagy/fusion assays; ROS; cytokines and barrier markers. |
| **29. Kanazawa et al., 2026** | Edaravone-loaded human-serum-albumin nanoparticles | Mitochondria-targeted or mitochondria-modulating antioxidants | Nano-antioxidant delivery system designed to concentrate antioxidant activity in injured/atrophic muscle and restore mitochondrial function. | SPARC-mediated delivery and lysosomal release; PGC-1α signaling; atrophy-pathway modulation. | SPARC siRNA; endocytosis/lysosomal inhibitors and trafficking; PGC-1α and MDA; myostatin/atrogin; muscle mass and endurance. |
| **30. Javadov et al., 2015** | XJB-5-131 | Mitochondria-targeted or mitochondria-modulating antioxidants | Molecule was designed and tested as a mitochondria-targeted antioxidant. | Respiratory-chain redox protection; preservation of single-fiber contractile quality. | Complexes I, III, and IV activities; mitochondrial protein oxidation; single-fiber unloaded shortening velocity and power. |
| **31. Vays et al., 2014** | SkQ1 | Mitochondria-targeted or mitochondria-modulating antioxidants | Mitochondria-targeted plastoquinone derivative designed to accumulate within mitochondria. | Mitochondrial ultrastructure and reticular organization; autophagic morphology. | Electron-microscopic mitochondrial reticulum/cristae integrity; abnormal mitochondria and autophagosome occurrence. |
| **32. Dong et al., 2022** | Ginsenoside Rb1 | Nrf2 pathway activators and redox-responsive phytochemicals | Redox-responsive phytochemical investigated through ROS-dependent NF-κB and mitochondrial-stress signaling. | NF-κB inflammatory signaling; mitochondrial dysfunction/biogenesis; apoptosis. | ROS, mitochondrial membrane potential, and apoptosis; p-NF-κB/p-IκB; mitochondrial biogenesis/function markers; docking analyses. |
| **33. Sciandra et al., 2023** | Verbascoside | Nrf2 pathway activators and redox-responsive phytochemicals | Nrf2/HO-1 activation was a central mechanistic hypothesis and was directly measured. | Mitochondrial respiratory reserve; cytoprotection against oxidative injury. | Nrf2 phosphorylation/nuclear translocation; HO-1 and PGC-1α; ROS and viability; maximal respiration and spare respiratory capacity. |

| Study | Intervention | Primary analytical category | Basis for primary assignment | Secondary mechanistic actions / overlapping categories | Supporting endpoints in the included study |
|---|---|---|---|---|---|
| **34. Choi, 2020** | Platycodin D | Nrf2 pathway activators and redox-responsive phytochemicals | Nrf2/HO-1 was the central tested pathway, including loss-of-function assessment. | Mitochondrial protection; DNA-damage limitation; anti-apoptotic signaling. | Nrf2 siRNA; Nrf2 nuclear accumulation and HO-1; ROS and mitochondrial membrane potential; DNA damage and apoptosis. |
| **35. Choi, 2021** | Trans-cinnamaldehyde | Nrf2 pathway activators and redox-responsive phytochemicals | Redox-responsive phytochemical investigated primarily through suppression of oxidative-stress-induced ROS signaling. | DNA-damage limitation; mitochondrial apoptosis regulation. | Intracellular ROS; DNA-damage markers; mitochondrial membrane potential and cytochrome c; Bcl-2/Bax and caspase-3. |
| **36. Sinha-Hikim et al., 2013** | F1 cystine-based glutathione precursor | Thiol-containing and glutathione-supporting strategies | Cystine-containing precursor formulation intended to restore glutathione-dependent redox capacity. | Mitochondrial antioxidant defense; apoptosis; AMPK/JNK/Akt and Notch-related signaling. | GSH/GSSG, 4-HNE, and SOD2; TUNEL; JNK, AMPK, Akt, Delta-1, and PCNA; muscle weight and myofiber CSA. |

**Note:** Primary categories were mutually exclusive for analytical assignment and study-level counting. Secondary mechanistic actions indicate experimentally reported overlap and were not counted as additional interventions. Secondary actions were recorded only when supported by findings from the included study, rather than inferred solely from the broader literature. Publications contributing both animal and cellular evidence are presented once. This table includes the 36 preclinical publications reporting mechanistic outcomes; included studies without mechanistic measurements are not listed.

**Classification rule:** Primary assignment followed the intervention identity/formulation, the dominant mechanistic rationale stated by the original investigators, and the principal endpoints used to test that rationale. Where more than one category was biologically plausible, the stated primary hypothesis and most direct mechanistic test determined the unique primary assignment. The framework is analytical and not biologically exclusive.

**Abbreviations:** 4-HNE, 4-hydroxynonenal; ACOX1, acyl-CoA oxidase 1; BCAA, branched-chain amino acid; CAT, catalase; CSA, cross-sectional area; ETC, electron-transport chain; GPx/GPX, glutathione peroxidase; GSH, reduced glutathione; GSSG, oxidized glutathione; HAE, 4-hydroxyalkenal; HO-1, heme oxygenase-1; MDA, malondialdehyde; NAC, N-acetylcysteine; Nrf2, nuclear factor erythroid 2-related factor 2; PQQ, pyrroloquinoline quinone; ROS, reactive oxygen species; SOD, superoxide dismutase; TBARS, thiobarbituric-acid-reactive substances.
